# Crossing cultural divides: A qualitative systematic review of factors influencing the provision of healthcare related to female genital mutilation from the perspective of health professionals

**DOI:** 10.1371/journal.pone.0211829

**Published:** 2019-03-04

**Authors:** Catrin Evans, Ritah Tweheyo, Julie McGarry, Jeanette Eldridge, Juliet Albert, Valentine Nkoyo, Gina Higginbottom

**Affiliations:** 1 School of Health Sciences, University of Nottingham, Nottingham, United Kingdom; 2 Libraries Research and Learning Resources, University of Nottingham, Nottingham, United Kingdom; 3 Department of Maternity and Obstetrics, Imperial College Healthcare NHS Trust, London, United Kingdom; 4 Mojatu Foundation, Nottingham, United Kingdom; Oakland University, UNITED STATES

## Abstract

**Introduction:**

As a result of global migration, health professionals in destination countries are increasingly being called upon to provide care for women and girls who have experienced female genital mutilation/cutting (FGM/C). There is considerable evidence to suggest that their care experiences are sub-optimal. This systematic review sought to illuminate possible reasons for this by exploring the views, experiences, barriers and facilitators to providing FGM-related healthcare in high income countries, from health professionals’ perspectives.

**Methods:**

Sixteen electronic databases/resources were searched from inception to December 2017, supplemented by reference list searching and suggestions from experts. Inclusion criteria were: qualitative studies (including grey literature) of any design, any cadre of health worker, from OECD countries, of any date and any language. Two reviewers undertook screening, selection, quality appraisal and data extraction using tools from the Joanna Briggs Institute (JBI). Synthesis involved an inductive thematic approach to identify descriptive themes and interpret these into higher order analytical constructs. Confidence in the review findings was assessed using GRADE-CERQual. The review protocol was registered with PROSPERO (CRD420150300042015).

**Results:**

Thirty papers (representing 28 distinct studies) from nine different countries were included. The majority of studies focused on maternity contexts. No studies specifically examined health professionals’ role in FGM/C prevention/safeguarding. There were 20 descriptive themes summarised into six analytical themes that highlighted factors perceived to influence care: knowledge and training, communication, cultural (mis)understandings, identification of FGM/C, clinical management practices and service configuration. Together, these inter-linked themes illuminate the ways in which confidence, communication and competence at provider level and the existence and enactment of pathways, protocols and specialist support at service/system level facilitate or hinder care.

**Conclusions:**

FGM/C is a complex and culturally shaped phenomenon. In order to work effectively across cultural divides, there is a need for provider training, clear guidelines, care pathways and specialist FGM/C centres to support mainstream services.

## Introduction

Globally, it is estimated that over 200 million women and girls are survivors of female genital mutilation (or cutting)–hereafter referred to as FGM/C [1]. FGM/C is categorised by WHO [2] into four types (I-IV), of differing degrees of severity. FGM/C is practised in 30 countries across North and sub-Saharan Africa and in parts of the Middle East and Asia [1]. However, Type III FGM/C (also referred to as infibulation and considered the most severe in terms of potential health consequences), is predominantly found in communities from the Horn of Africa.

As a result of increasing global mobility and migration, health professionals in high income countries are called upon to work across cultures and to provide care to highly diverse population groups [3–6]. Countries that receive migrant or refugee populations are often referred to as ‘host’ or ‘destination’ countries [7]. Increasingly, their migrant populations include women or girls who have undergone FGM/C. For example, within Europe, over half a million women and girls are thought to have experienced FGM/C [8], and, in the UK, it is estimated that, since, 2008, women with FGM/C now make up approximately 1.5% of all maternity episodes [9].

FGM/C is associated with significant negative physical, psychological and sexual health sequelae [10–12]. In the short term, these include infection, haemorrhage, urinary retention or injury to other tissues (e.g. vaginal fistulae). In the longer term, they include psychological problems, post-traumatic stress disorder, painful intercourse and other sexual problems, relationship problems, chronic pain, chronic infections, infertility and complications in childbirth [13–16]. There is particular concern to ensure that pregnant women with type III FGM/C are identified and offered deinfibulation prior to delivery in order to avoid any obstetric or neonatal complications. The optimal timing of deinfibulation however (antepartum or intrapartum) is currently still unclear [15, 17–19]. Hence, it is essential that women and girls affected by FGM/C have access to services that can identify and meet these multiple complex health needs, and that include mental as well as physical healthcare provision [11, 12, 20, 21].

Over the last decade, many destination countries have implemented laws, policies and programmes to prevent FGM/C and to develop services to provide appropriate healthcare [22, 23]. These have involved health professional training initiatives and the development and implementation of relevant clinical guidelines [24–27]. In some countries, such as the UK, statutory requirements have been introduced for health professionals to record any cases of FGM/C that are identified in their services and to safeguard children thought to be at risk of FGM/C through processes such as mandatory reporting to the police [22, 23, 28–30].

In spite of these initiatives, there is a growing body of evidence to suggest that many migrant FGM/C survivors endure poor healthcare experiences [31–33]. These are associated with a perceived lack of clinical and cultural competence amongst healthcare providers (such as poor communication about FGM/C or poor management of clinical procedures such as deinfibulation) which in turn leads to women feeling disrespected, stigmatised and vulnerable. In order to illuminate the reasons for this, there is a need to better understand the experiences of healthcare professionals when they encounter women with FGM/C and their views on factors that may influence care quality and service provision.

Existing systematic reviews on health provider perspectives confirm a prevailing lack of knowledge, competence and understanding about FGM/C [34–36], however, we suggest there is a need to extend the reach and focus of these existing reviews for four reasons. Firstly, existing reviews have included studies from across the world rather than ‘destination’ countries only, making it hard to judge the transferability of their findings to host country health systems. Secondly, existing reviews have not included grey literature, thus potentially excluding relevant research. Thirdly, the main focus of existing reviews has been on maternity contexts, and little is known about factors that may influence access to care, identification of FGM/C or care provision in other clinical settings. Finally, existing reviews have relied heavily on quantitative evidence which, whilst highlighting trends, is unable to provide a more nuanced picture of barriers or facilitators of service provision. The need to consider wider factors in understanding health professionals’ practice is illustrated in several studies. For example, a 2013 study of FGM/C management in a large London maternity unit [37] found that, in spite of the existence of protocols, guidelines and training, clinical care for women/girls with FGM/C was sub-optimal. The maternity unit had access to a FGM/C specialist service, but 41% of women with FGM/C were not identified until they arrived in the labour ward. Hence, even though a specialist service existed, it was not being optimally used to benefit women with FGM/C, and a significant percentage of opportunities were missed to provide women with specialist care. Similar findings were reported from a study in a maternity unit in Switzerland where, in spite of staff training and the existence of clear guidelines, FGM/C was correctly identified and managed in only 34 (26.4%) of 129 cases reviewed [38]. In Australia, a study based in a large metropolitan obstetric unit found only 35% of its database records accurately recorded a patient’s FGM/C status [39]. Likewise, an audit in Lothian in Scotland (between 2010–2013), showed that of 487 women from FGM/C practising countries, only 18% had any documentation relating to FGM/C, suggesting that opportunities for detection may have been missed [40]. The reasons for this lack of adherence to protocols are unclear and warrant further investigation.

### Aim

In order to gain a greater understanding of factors that influence healthcare provision for FGM/C in high income migrant destination countries, we undertook a comprehensive systematic review of qualitative evidence. The specific aim was to explore the experiences, needs, barriers and facilitators to providing FGM-related healthcare from the perspective of health professionals.

This project was co-developed from an existing collaboration between an academic team (CE, RT, GH, JMc, JE), clinical experts (JA) and a community organisation working on FGM/C and gender rights issues (VN). By undertaking the review we hoped to illuminate the experiences of health professionals and thus provide evidence to inform training and service development initiatives.

## Methods

This qualitative systematic review is reported following the ENTREQ guidelines [41]. The review was registered in PROSPERO [42] and the methods are documented in detail in a published protocol [31].

### Search strategy

The literature search was based on an exhaustive and sensitive five-step strategy. Firstly, three electronic resources were searched using a combination of index terms and text-based queries. This was done in order to assess the search terms, to initiate the searching, to establish the scope of terminology and to formulate a consistent search strategy to be applied across subsequent individual databases. Secondly, eight electronic databases were searched from inception to a cut-off date of 31-12-17 (see S1 Table for an example search strategy for Ovid MEDLINE). Thirdly, searches for relevant grey literature were undertaken via five online resources. Fourthly, additional searches were undertaken in Google and Google Scholar and by soliciting suggestions from the project’s expert advisory group. Finally, hand-searches of the reference lists of relevant systematic reviews and all the included studies were undertaken (see S2 Table for a list of all resources/databases that were searched) [43–46]. All retrieved datasets were downloaded into an EndNote library and de-duplicated.

### Screening and selection

The inclusion criteria of studies for the review were: (i) any date, (ii) any language, (iii) empirical research, (iv) qualitative research (of any design/methodology, including qualitative findings from mixed methods studies), (v) OECD country setting (OECD is commonly considered a proxy for comparable high income ‘destination’ countries as this group of countries generally share universal health coverage systems and similar social-political values [47]), (vi) explicitly reporting views or experiences with providing healthcare or advice associated with FGM/C, (vi) any clinical setting, (viii) any cadre of health professional.

All titles and abstracts were independently assessed against the inclusion criteria by two team members working in pairs as part of a four member team (RT&CE, RT&GH and RT&JMG). Full-text versions of papers deemed to be potentially relevant were obtained and scrutinised by two team members (RT&CE). Papers that did not meet the inclusion criteria were excluded with reasons noted (see S3 Table for a full list of these). Ambiguous papers were discussed with the wider project team until agreement was reached. Non-English language papers that appeared potentially relevant (on the basis of their English abstract) were fully translated by professional translators.

### Quality assessment

Study quality was independently assessed by pairs of two reviewers working as part of a four member team (RT&CE, RT&GH and RT&JMG) using the Joanna Briggs Institute Qualitative Assessment and Review Instrument (JBI-QARI) [48, 49]. Grey literature reports and theses were similarly appraised [43, 45, 46, 50, 51]. As per recommended guidance, studies were not excluded on the grounds of quality [48, 50]. The quality assessment was used to establish a detailed understanding of the strengths and weaknesses of each study and how this might impact on its findings and on the synthesis. It was also an important part of the process of assessing confidence in the review findings (GRADE-CERQual) [52–56].

Following an approach outlined by Higginbottom et al [57, 58], each paper was individually critically appraised and given an aggregate score out of ten using the JBI QARI tool [49]. Within each two-person team, the reviewers compared their respective quality assessments and, where there were divergences, a discussion was held to reach agreement. Where ambiguities remained, the paper was shared and discussed with the whole team to achieve a final consensus rating. Papers were categorised as high quality (above seven), medium quality (between five-seven) or low quality (under five). Whilst recognising the potential problems with ‘scoring’ qualitative papers [50, 54, 55], the rating was undertaken to enable the team to achieve an overview of the relative quality of included papers and also to facilitate the application of the CERQual evaluation [52].

To overcome some of the limitations of relying upon a checklist to assess quality, each study was also assessed in terms of its relative ‘richness’ in terms of potential contribution to the synthesis. This is a methodological approach first described by Popay et al [59], and operationalized further in Noyes & Popay [60] and Higginbottom et al [57, 58]. Richness was conceptualised as *“the extent to which the study findings provide explanatory insights that are transferable to other settings”* p.230 [60]. The richness of each paper was independently assessed by a two member team (RT&CE) as ‘thick’ or ‘thin’ following criteria defined by Higginbottom et al, p.5 [57] (see Table 1). The team compared ratings and discussed each paper until consensus was reached.

**Table 1:** Assessment of study richness.

Richness	Operational Definition
Thick papers	Offer greater explanatory insights into the outcome of interest Provide a clear account of the process by which the findings were produced—including the sample, its selection and its size, with any limitations or bias noted—along with clear methods of analysis Present a developed and plausible interpretation of the analysis based on the data presented.
Thin papers	Offer only limited insights Lack a clear account of the process by which the findings were produced Present an underdeveloped and weak interpretation of the analysis based on the data presented

### Data extraction and assessment of relevance

Domains from the JBI data-extraction tool were used as a template for data extraction (undertaken in Excel and covering items such as study aim, sample, methodology, methods, country/location) [48, 61]. Study characteristics were extracted in full by one team member (RT) with CE double checking each one for accuracy. At the same time, during the initial in-depth reading, each paper was assigned a level of relevance to the review question—as high, medium or low (see S4 Table for the operational criteria of relevance). Assessment of relevance was undertaken to facilitate a better understanding of the nature of the body of evidence, and also to assist with coding (described in more detail below). The assessment of relevance was undertaken primarily by RT, who discussed any queries with CE. Full-text PDFs of all the included papers were imported into NVivo and the ‘findings/results’ and ‘discussion’ sections were coded and analysed [62].

### Data analysis and synthesis

The review followed an inductive thematic synthesis approach as described by Thomas and Harden [63]. This was an iterative process involving four inter-linked stages: (i) in-depth reading of the whole papers, (ii) line by line coding of study findings, (iii) assembling the codes on the basis of similarities in meaning into descriptive themes, and, (iv), interpreting higher order analytical themes. The analysis started with papers that had been categorised as ‘thick’ and ‘highly relevant’ to develop a code book which was then applied across the other papers and further elaborated as appropriate [64, 65].

### Rigor within the analytical process

A number of steps were taken to maximise rigor within the analytical process. Firstly, the team actively tried to identify and explain any ‘disconfirming’ cases that might challenge evolving interpretations [66] and to explore significant sub-group or contextual differences. Due to the relatively large number of included papers, these steps were facilitated by the development of a theme matrix (see S5 Table), in which each theme was mapped to its constituent studies. This indicated how common the theme was amongst the studies, what kind of contexts or populations it related to, and why it may have been prominent in some studies but not in others. Secondly, the evolution of the synthesis was undertaken as a collaborative process, involving the whole team and wider project advisory group who read selected papers and contributed to the development of key interpretations. Thirdly, a national stakeholder event was held to provide additional feedback on the review findings and to contribute to the recommendations [67].

### Assessment of confidence in the review findings (CERQual)

The CERQual approach (Confidence in Evidence from Reviews of Qualitative Research) was used to evaluate the level of confidence in the review findings [50, 52, 68–74]. Similar to GRADE, CERQual assesses ‘concerns’ within four domains applied to each individual review finding (methodological limitations, relevance, coherence and adequacy of data) [71]. The CERQual assessment was initially undertaken as a joint process by CE and RT. The assessments were then discussed and agreed with the wider team.

## Results

### Search outcome

**Fig 1:**
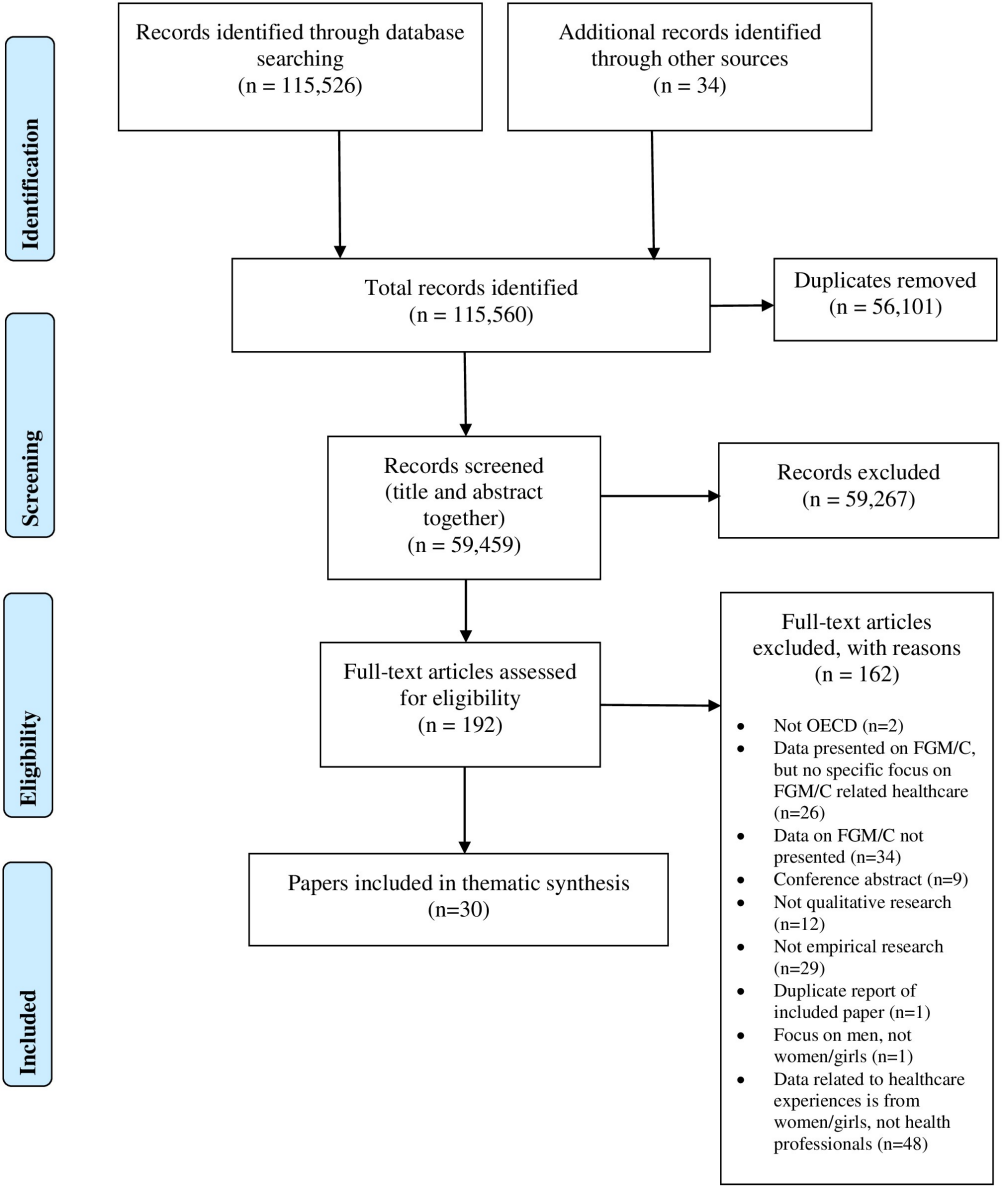
PRISMA flow diagram.

Thirty papers met the inclusion criteria, representing 28 distinct studies. Twenty four of the 30 papers were published in English; five were published in Swedish [75–79], and one in Spanish [80]. The PRISMA flow diagram (Fig 1) presents the results of the search in detail.

### Study characteristics

There were a relatively high number of studies in this review. Hence, for ease of reading, a highly summarised account of study characteristics and associated methodological assessments is presented in Table 2. Full details are provided in S6 and S7 Tables.

**Table 2:** Summary study characteristics and methodological assessments.

Study No.	Author/Date	Country	Focus or aims pertinent to the review question (sometimes only one aspect of a larger, broader study)	Eligible participants (health professionals only, not necessarily the whole sample)	Quality rating	Richness	Relevance
**1.**	Abdi, R. (2012) [96]	UK	To explore responses of Somali women within a healthcare setting.	2 gynaecologists, 1 counsellor & 1 midwife	High	Thin	Low
**2.**	Behrendt, A. (2011) [86]	Germany	To explore where and how women with FGM/C seek medical care and/or participate in preventive programmes.	13 health professionals: (6 female gynaecologists and a mix of other health personnel, including midwives, nurses, dermatologist, paediatrician)	Med	Thin	Low
**3.**	Bergqvist, H., & Svensson, J. (2016) [75]	Sweden	To highlight midwives’ experiences at youth clinics when they meet with women with FGM/C.	8 midwives	Med	Thin	High
**4.**	Bibi, N., & Rahimian, N. (2013) [76]	Sweden	To investigate nurses’ knowledge and experience of FGM/C.	11 nurses: (6 nurses employed at gynaecological departments, 1 school nurse, 1 health centre nurse and 3 nurses working in a geriatric department)	Low	Thin	High
**5.**	Brodin, E., & Mårtensson, N. (2016) [77]	Sweden	To describe the knowledge and experiences of district nurses in caring for women with FGM/C.	9 female district nurses	High	Thin	High
**6.**	Bulman, K. H., & McCourt, C. (2002) [98]	UK	To explore professionals’ perceptions of Somali women and their maternity care needs.	2 focus groups with midwives (number not stated). Individual interviews with 3 others (a Somali health-link worker, a woman on the Health and Race Working Group, and an obstetric registrar)	Med	Thin	Med
**7.**	Bulman, K., & McCourt, C. (1997) [97]	UK	To explore professionals’ perceptions of Somali women and their maternity care needs and views on service improvement.	2 focus groups with midwives (number not stated). Individual interviews with 3 others (a Somali health-link worker, a woman on the Health and Race Working Group, and an obstetric registrar)	Low	Thin	Med
**8.**	Burchill, J., & Pevalin, D. J. (2014) [99]	UK	To explore the experiences of health visitors working with refugee and asylum seeking families.	14 health visitors	High	Thick	Low
**9.**	Byrskog, U., Olsson, P., Essen, B., & Allvin, M. K. (2015) [91]	Sweden	To explore ways antenatal care midwives in Sweden work with Somali born women and the questions of exposure to violence.	17 midwives	Med	Thick	Med
**10.**	Dawson, A. J., Turkmani, S., Varol, N., Nanayakkara, S., Sullivan, E., & Homer, C. S. (2015) [85]	Australia	To provide an insight into midwives views and experiences of working with women affected by FGM.	48 midwives	Med	Thick	High
**11.**	Fawcett, L. (2014) [101]	USA	To explore the subjective and intersubjective perceptions of female circumcision.	10 health professionals: (2 medical doctors; 1 midwife, 2 nurse practitioners, and 5 labour and delivery nurses)	High	Thick	High
**12.**	Gertsson, M., & Serpan, H. (2009) [78]	Sweden	To explore professionals’ views and strategies around FGM/C.	1 midwife	Med	Thin	Low
**13.**	Holm, L., & Kammensjö, H. (2012) [79]	Sweden	To highlight school nurses’ experience of FGM in schools.	11 school nurses	Med	Thin	Med
**14.**	Hussen, M. A. (2014) [87]	New Zealand	To explore views and experiences around FGM.	3 health providers: (GP, nurse & health social worker).	Med	Thick	High
**15.**	Jatau, M. (2011) [102]	USA	To explore views and experiences of working with African refugee women.	10 female health care providers: (1 obstetrician/ gynaecologist, 2 health social workers, 2 certified nurse midwives, and 5 registered nurses)	High	Thick	Med
**16.**	Johansen, R. E. (2006) [88]	Norway	To explore experiences and management of birth care of infibulated women.	40 health workers: (25 midwives, 9 gynaecologists, 3 general practitioners & 3 nurses)	Med	Thick	High
**17.**	Johansen, R. E. (2017) [89]	Norway	To explore the factors that encourage and hinder women and girls from seeking medicalized deinfibulation.	30 health professionals: (included employees from health clinics that conducted deinfibulation, school nurses, sexual counsellors for youth, and other refugee and asylum seeker personnel)	High	Thick	Med
**18.**	Lazar, J. N., Johnson-Agbakwu, C. E., Davis, O. I., & Shipp, M. P. L. (2013) [103]	USA	To explore training needs and experiences of working with women with FGM/C.	14 health providers: (9 obstetricians and 1 family practice physician who was Somali, 3 nurse midwives, and 1 nurse practitioner)	Med	Thick	High
**19.**	León-Larios, F., & Casado-Mejía, R. (2012) [80]	Spain	To explore midwives’ views & experiences of FGM/C.	24 midwives	Med	Thick	Med
**20.**	Leval, A., Widmark, C., Tishelman, C., & Maina Ahlberg, B. (2004) [92]	Sweden	To investigate midwives’ perceptions, attitudes and experiences around FGM/C.	26 midwives	High	Thick	High
**21.**	Moore, K. (2012) [100]	UK	To elicit the opinions and experiences of midwives with regard to providing culturally competent care for women who have undergone FGM.	4 midwives	Low	Thin	High
**22.**	Ogunsiji, O. (2015) [81]	Australia	To explore the knowledge and attitude of Australian midwives caring for women living with FGM.	11 midwives.	Med	Thin	Low
**23.**	Ogunsiji, O. (2016) [82]	Australia	To report Australian midwives’ stories about how they manage obstetric care of women living with FGM.	11 midwives,	High	Thin	High
**24.**	Rubin, E. A. (2000) [104]	USA	To explore factors that influence communication and care for women with FGM/C.	10 female healthcare providers: (5 nurse-midwives, 3 paediatricians, 1 internist, and 1 nurse practitioner)	High	Thick	High
**25.**	Thierfelder, C. (2003) [95]	Switzerland	To find out what key health care providers of different professions think about FGM/C, and their readiness to provide support.	37 health providers: (17 midwives, 20 physicians; 17 gynaecologists/ obstetricians, 3 GPs)	High	Thick	High
**26.**	Vangen, S., Johansen, R. E. B., Sundby, J., Traeen, B., & Stray-Pedersen, B. (2004) [90]	Norway	To explore how perinatal care practice may influence labour outcomes among circumcised (Somali) women.	36 health care professionals: (8 gynaecologists, 22 midwives, 3 public health doctors, and 3 public health nurses).	High	Thick	High
**27.**	Vaughan, C., White, N., Keogh, L., Tobin, J., Ha, B., Ibrahim, M., & Bayly, C. (2014) [83]	Australia	To improve understanding of the impacts of FGM and to make suggestions for service development.	11 health service providers: (a senior women’s health clinician, senior clinical midwife, 2 obstetrician/ gynaecologists, a GP, community midwife and 4 community outreach workers)	High	Thick	High
**28.**	Vaughan, C., White, N., Keogh, L., Tobin, J., Murdolo, A., Quiazon, R., & Bayly, C. (2014) [84]	Australia	To build evidence as to the training, education and professional development required for service providers.	15 health service providers: (3 medical consultants, 4 GPs, 1 senior midwife, 1 sexual health practitioner, 1 midwife, 3 refugee health workers, community health worker and a community development worker)	High	Thick	High
**29.**	Widmark, C., Leval, A., Tishelman, C., & Ahlberg, B. M. (2010) [93]	Sweden	To explore obstetricians’ perspectives on caring for women with FGM.	19 obstetricians: (13 senior obstetricians & 7 senior house officers)	High	Thick	High
**30.**	Widmark, C., Tishelman, C., & Ahlberg, B. M. (2002) [94]	Sweden	To investigate Swedish midwives’ experiences of caring for infibulated women.	26 midwives	High	Thick	High

The studies represented nine different OECD countries: Australia [81–85], Germany [86], New Zealand [87], Norway [88–90], Spain [80], Sweden [75–79, 91–94], Switzerland [95], UK [96–100] and the USA [101–104]. Sweden contributed the most papers to the review (n = 9), followed by the UK (n = 5) and Australia (n = 5). The other countries contributed between one and three papers each.

The studies covered a wide range of publication dates, covering the period between 1997 and 2017. However, two thirds of the papers (n = 20) had been published since 2011, hence the majority of the papers reflected a more contemporary context.

Some studies focused on a single cadre of healthcare professional whereas others included multiple cadres. The samples were as follows: midwives (n = 9) [75, 78, 80–82, 85, 91, 92, 94, 100], health visitors (n = 1) [99], district/community nurses (n = 1) [77], mixed group of nurses (n = 1) [76], school nurses (n = 1) [79], doctors (n = 1) [93] and mixed groups of health professionals (n = 14, including doctors from a variety of specialisms, nurses and midwives) [83, 84, 86, 87, 89, 95, 96, 102, 104] [88, 90, 97, 98, 101, 103]. Within the 14 ‘mixed’ sample papers, in addition to midwives, nurses and doctors, two studies included counsellors [89, 96] and four included community health and social workers [83, 84, 89, 97, 98]. There were no studies that included students. Notably, only two studies included any kind of mental health professional perspective, however, the findings were not sufficiently differentiated to explore their views as distinct group [89, 96].

The studies had varied research aims and foci. Some focused directly on professionals’ experiences of supporting/managing women who have undergone FGM/C (e.g. [88]), whereas others were more indirect (e.g. [103]). For example, some studies focused on the care of refugee women in general, but included aspects relating to FGM/C (e.g. [99, 102]). Eleven papers specifically focused on professionals’ experiences with women from countries where infibulation is commonly practised (i.e. women who have experienced type III FGM/C) [88–91, 93, 94, 96–98, 101, 103]. However, the findings of most of the papers reflected a sense that professionals were often conflating FGM/C in general with type III specifically. Hence, it is our contention that the review findings are skewed towards healthcare professionals’ experiences of, and views on, FGM/C type III.

There were no studies that examined health professionals’ views/experiences of cervical screening for women who have experienced FGM/C. There were also no qualitative studies that explored professional views on surgical reconstruction following FGM/C. Only one study (of school nurses) focused explicitly on professionals’ views of supporting younger women or girls who had undergone FGM/C [79].

### Methodological quality

A summary of methodological assessments (quality rating, relevance, richness) is included in Table 2. Full details of all methodological assessments can be found in S7 Table. Fifteen papers were assessed as being of high quality [77, 82–84, 90, 92–96, 99, 101, 102, 104], 12 papers were assessed as medium quality [75, 78–81, 85–88, 91, 98, 103], and three papers were assessed as low quality [76, 97, 100]. Twelve papers were classified as ‘thin’ [75–79, 81, 82, 86, 96–98, 100], and 18 papers were classified as ‘thick’ [80, 83–85, 87–95, 99, 101–104]. The thicker papers tended to be studies that were informed by an anthropological theoretical approach, that had followed a clear methodology or that had moved beyond mere description in their analysis towards a more interpretive and analytical account of the phenomenon of interest.

A common methodological weakness was that many studies did not describe a philosophical standpoint (question one of the QARI tool), making it difficult to assess the congruency of the chosen methodology. In addition, many studies did not mention whether they had followed a particular methodological genre (often just stating that they had used a generic ‘qualitative approach’). This made it hard to judge the congruence of the methodology with the research question and the methods (questions two and three on the QARI tool). Finally, a common weakness was that many studies omitted any discussion of reflexivity (questions six and seven on the QARI tool). As FGM/C is a highly sensitive and politicised topic, the failure to explore the researcher’s own theoretical standpoint made it hard to judge the dependability of the findings [105]. Likewise, the researcher’s own professional background (as insiders or outsiders to a profession for example), may have influenced their viewpoint, their ability to build rapport with their participants or their interpretation of the findings, but this was rarely discussed.

### Thematic synthesis findings

The findings from 30 papers were synthesized into six analytical themes which represent a synthesis and interpretative analysis of 20 descriptive themes. These analytical and descriptive themes are presented in Table 3. In this table, each descriptive theme is presented along with references to its underpinning papers, its CERQual assessment and one or two direct quotes that illuminate its meaning more fully and demonstrate its grounding in the data. Because quotes are presented in Table 3, the narrative account of the synthesis below does not also include quotes. Rather, each analytical theme is briefly described, followed by a detailed explanation of its constituent descriptive themes. Due to the relatively high number of papers that contributed findings to each descriptive theme, for ease of reading, we have chosen not to insert multiple repetitive references to these individual studies within the text. Instead, the reader is referred back to Table 3 and also to a detailed theme matrix (S5 Table), both of which clearly show which papers have contributed findings to each theme. Hence, references are only included where particular studies illuminate a particular nuance within a theme.

**Table 3:** Themes, quotations and CERQual assessment.

Theme No.	Theme Heading	No. of Studies	CERQual Assess-ment	Indicative Quotes
**Analytical theme 1: Knowledge and training**				
1.1	Knowledge and awareness	n = 25 [75–90, 92–95, 98, 100–103]	High Confidence	*Two midwives also described having ‘‘mini panic attacks” when they saw the sticker on the women’s notes indicating that they had FGM/C, as they felt that their practice was inadequate. Midwives also stated that they did not have enough experience to identify FGM/C, saying it was not always ‘‘clear-cut” to classify. Australia, p.211* [85]
1.2	Education and training	n = 21 [76, 77, 79–81, 83–87, 90, 93–95, 97, 99, 100, 103]	High Confidence	*“You sort of get dropped into it, I think we try to talk when there’s a patient that we know is Somali whose having her 1st baby and is going to have a significant tear, I think we try to talk about how to manage that when we can.” (Female OB/GYNB, resident). USA, p.6* [103]
**Analytical Theme 2: Communication is key**				
2.1	Language barriers and interpretation challenges	n = 20 [75–77, 79, 80, 83–86, 91, 93–95, 98, 100–104]	High Confidence	*“You don’t have time with all the interpreter situations……….So the care they receive is definitely not as good, that’s for certain…..and you can miss an incredible amount because of that, and maybe miss that it’s not a normal pregnancy”. [Obstetricians], Sweden, p.556* [93]
2.2	Talking about a sensitive topic	n = 24 [75–80, 82–85, 87, 88, 90–92, 94, 95, 98–104]	High Confidence	*“The female genital mutilation is a very diffcult one. … I have had clients with that and it’s asking the question and I think again I found that the few people that I did ask quite often had had it—when asked they were very open about it. I remember, until I had the training on it, not being confident about asking and I found that really useful to do”. [Health Visitor], England, p.155* [99]
2.3	Women also find FGM/C hard to talk about	n = 13 [76, 79, 82–85, 87, 90, 91, 95, 101, 103, 104]	Moderate confidence	*“I think they do not ask a lot, because they feel their difference. They know that in Switzerland there is certain astonishment. I think that they do not ask because they feel that they are different.” [Gynaecologist], Switzerland, p.55* [95]
**Analytical Theme 3: Encountering the ‘other’ in clinical practice: negotiating cultural dissonance and achieving cultural understanding**				
3.1	Attitudes towards FGM/C: mixed emotions	n = 17 [75, 76, 78, 79, 81, 83, 85, 88, 90, 92–96, 100, 101, 103, 104]	High confidence	*“As a woman yourself, you kind of feel….a sorrow………I take care of them just like all the others, and perhaps try to be more empathic and….kinder… … It is diffcult, because you’re so angry. You get so … get enraged at the whole situation, at the whole culture … how the hell can they subject women to that ….. I become furious at men….I try not to show it”. [Midwives]. Sweden, p.117* [94]
3.2	Cultural dissonance–control and resistance in clinical encounters	n = 20 [76, 78–80, 83–85, 88–95, 98, 101–104]	Moderate confidence	*“While other patients follow orders, those from the ‘Somali culture’, they take their time. If they don’t want to get in bed they don’t get in bed. This is perceived as the patient being defiant and resisting orders……………..there is a huge control element in healthcare that is necessary to maintain order and safety and maintain their process.” [Nurse Practitioner], USA, p.233* [101]
3.3	Acknowledging the role of the family	n = 15 [76, 77, 80, 82, 85, 88, 92–95, 97, 100, 101, 103, 104]	High confidence	*One midwife described the influence of an older woman on the decision to have a de-infibulation prior to birth, citing that ‘‘their mother or aunty said no.” Australia, p.210* [85]
3.4	Gender of the provider	n = 8 [77, 80, 82, 84, 94, 95, 101, 102]	Moderate confidence	*“In this matter, female midwives are best because the women completely refuse to be seen by men. There is a complete gender-based affinity between women” [Midwife], Spain,* [80]
3.5	Crossing the cultural divide–strategies and elements of culturally sensitive care	n = 23 [75–79, 81–87, 91, 93–95, 98–104]	High confidence	*“It’s important that I understand what the girls or women have gone through and how they grew up. This is in order to get the whole story from her perspective and to try and meet halfway…. You can imagine that this must be difficult for them to talk about……You can’t always treat everyone the same…. you have to take each case as it is”. [Midwife], Sweden,* [76]
**Analytical Theme 4: Identifying FGM/C: hit and miss**				
4.1	Presentation and help seeking	n = 15 [75–78, 80, 82–87, 94–96, 104]	Moderate confidence	*“I didn’t think I needed to bring up the question with them if they didn’t themselves bring it up. I would never ask a Swedish lady about her gynaecological problems if she herself hadn’t brought it up. It’s like this, if we know she is pregnant then I know that she will meet the midwife and it will be there that she might talk about it, if she wasn’t going to bring it up herself.” [Gynaecologist], Sweden,* [78]
4.2	Practices and processes around identifying FGM/C	n = 20 [75–77, 80, 82–85, 87, 88, 90, 91, 93–95, 98, 100–103]	Moderate confidence	*Time pressures and lack of experience were seen as barriers to data collection………In one hospital, labels were placed on women’s files to indicate FGM. This was entered onto the computer under an alert function. However, one midwife admitted that these were not properly assessed by staff (FGD 3) Some midwives were aware of where they could record FGM on the database under ‘‘other surgeries” but felt that it was often missed and that FGM needed its own indicator, or a ‘‘direct question” for clarity. Participants were not supportive of additional forms, as there were too many already (FGD 4). Australia, p.212* [85]
**Analytical Theme 5: Clinical management practices: inconsistent and variable**				
5.1	Deinfibulation timing	n = 8 [82, 84, 85, 90, 93–95, 100]	Moderate confidence	*Midwives discussed the importance of including women in the management of their care. One midwife acknowledged the importance…..of “giving them options”. “Women should be involved in their plan of care right from the antenatal stage.” In discussions about defibulation women should be asked when they would like it done, midwives should discuss the feasibility of their request and should note the woman’s wishes for intervention and pain relief. UK, p.35* [100]
5.2	Deinfibulation practice	n = 21 [75–80, 82–85, 87, 88, 90, 93–95, 98, 100–103]	Moderate confidence	*Other participants were worried about undertaking clinical procedures that they were not confident with……‘‘I had to actually do an anterior episiotomy on her. I found that very unnerving to actually have to cut upwards. …. …The fear is how far is it going to extend and what it’s going to do.” Australia, p.210* [85]
5.3	Reinfibulation ambivalence	n = 11 [78, 81, 82, 85, 88, 90, 92–95, 100]	Moderate confidence	*“How should the midwife act? To sew the women up again is inconsistent with Swedish law, but at the same time the women must be respected as an individual”. Sweden,* [78]
5.4	Need for guidelines	n = 15 [75, 77, 79, 80, 82–85, 90, 93–95, 98, 100, 103]	Moderate confidence	*None of the clinical sites where study participants worked had formal protocols on the management of circumcised women. One participant mentioned that they had considered adopting a protocol to address requests for reinfibulation……however, the protocol was never created. USA, p.7* [103] *“Nobody has mentioned anything to me about a protocol”* (GD2 female midwife), Spain, p.5 [80]
5.5	Psychological issues	n = 12 [75, 88, 98] [76, 77, 82, 83, 85, 86, 90, 95, 101]	Moderate confidence	*“This lady had a small tear in the perineal muscle and I was actually stitching her up, lots of local anaesthesia. And she was crying and crying, and afterwards I said, “You know, like. I kept saying, ‘Are you okay,’” but she told me it brought back all those memories, you know, being sewn up before. So that was distressing, how she felt”. (Midwife 6), Australia, p.1162* [82]
**Analytical Theme 6: Optimal service development for FGM/C care**				
6.1	Provider’s role in prevention	n = 13 [76–79, 83, 85, 86, 89, 90, 92, 94, 95, 100]	Low confidence	*For example, one informant was a school nurse who had run numerous discussion groups on FGM/C for youth on sexuality. When asked whether sexual concerns and the motivation for FGM/C were topic for reflection and discussion in her groups, she was surprised by her own omission. She simply had not considered these topics. Her focus had been on the law and the health risks associated with FGM/C. Norway, p.8* [89]
6.2	Community engagement and education	n = 11 [86, 89] [76, 78, 79, 83–85, 87, 95, 100]	High confidence	*“Sometimes we have success stories that will tell us like, you know, ‘I used to be very strongly attached to my cultural practice, but now thank God, because I have read or because I have attended classes or because of the way how I had my children in the hospital, I don’t want this to be repeated to my daughter’..….. You know, it will change their mentality” (FARREP worker). Australia, p.27* [83]
6.3	Specialist services	n = 9 [76, 83–85, 87, 91, 94, 95, 100]	Moderate confidence	*“We need to be able to at least have a contact where we can ring or refer a patient like this to an area where there’s expertise….” [Medical consultant], Australia, p.14* [84] *“There is a huge disparity in the care available to a women with FGM in Bristol versus a women with FGM in Glasgow. Scotland’s migrant population are suffering because of an overwhelming failure to address this issue …” [Midwife], UK, p.40* [100]

#### Analytical theme 1: Knowledge and training

The review found that for health providers, feeling confident and able to deliver appropriate FGM/C-related care was strongly linked to having adequate knowledge, skills and training.

**Descriptive theme 1.1: Knowledge and awareness.** This theme was reported in 25 studies. Many studies described a lack of provider awareness around FGM/C or provider reports of having insufficient, inaccurate or partial knowledge and skills (especially around deinfibulation). This led to misconceptions, lack of awareness, fear and uncertainty about how to talk about FGM/C and how best to support women with FGM/C. In particular, lack of knowledge meant that providers may not even be aware that FGM/C was an issue that may need to be considered or addressed with a particular client, hence opportunities for care could be missed. This finding was reported amongst medical as well as nursing and midwifery staff. Several studies noted that providers may lack knowledge around differentiating between the different types of FGM/C (particularly type I and II) [76, 85, 86].

**Descriptive theme 1.2: Education and training.** Professionals in 21 studies identified a perceived need for greater education and training in all aspects (cultural, clinical, legal) associated with the management of women and girls with FGM/C, and that training would enhance provider confidence [99]. Health providers identified a lack of basic (pre-service) education (or an input that was too brief and superficial) and a need for regular CPD around FGM/C that included in-depth information, practical skill development and access to mentorship and clinical supervision where relevant.

#### Analytical theme 2: Communication is key

Providers reported multiple challenges in talking about FGM/C with women but recognised that good communication was key to providing quality care. Language barriers and challenges with interpretation were reported as a significant hindrance to effective communication. In addition, the nature of FGM/C, as a taboo, sensitive and specialised topic, was seen to add another layer of complexity. Communication problems led both providers and women to avoid the topic of FGM/C, thereby hindering the identification of FGM/C. Communication challenges also led to difficulties in forming quality trusting relationships with women and providing women with appropriate information and choice about their care.

**Descriptive theme 2.1: Language barriers and interpretation challenges.** This theme, reported in 20 studies, highlighted that language barriers were an issue that could significantly compromise care. Language was particularly an issue with women who were still relatively recent migrants. The language barrier affected providers’ ability to elicit in-depth information from women or to provide in-depth explanations and engage in shared decision making. Moreover, language barriers deterred providers from asking questions about FGM/C at all—as it was seen to be too difficult to try and address such a sensitive topic requiring specialist vocabulary in a short consultation.

Use of interpreters was variable and inconsistent [77, 79, 83–85, 93–95, 97, 98, 100, 103, 104]. Some providers preferred to rely on informal interpreters who might accompany women. However, others expressed concern about maintaining women’s confidentiality given that communities could be tight knit, and queried women’s ability to open up honestly in front of known family/community members (especially if the husband was acting as an interpreter) [79, 83, 84, 97, 98, 103, 104]. Providers also had concerns about the accuracy and quality of interpretation (both with formal and informal interpreters) given the specialist nature of FGM/C, the niche vocabulary and the sensitivity of the topic [79, 83, 84, 97, 98, 104]. Time constraints were also cited as obstacles to using an interpreter, as interpretation inevitably lengthened the consultation time, yet providers were often not given additional time. Finally, using formal interpretation services affected the provider’s ability to establish continuity of care, as a different interpreter might be available each time.

**Descriptive theme 2.2: Talking about a sensitive issue.** This theme, reported by 24 studies, refers to the sensitive and taboo nature of FGM/C due to its association with sex, sexuality and ‘private parts’. The nature of the topic compounded potential language barriers and made FGM/C hard to talk about, even when a language barrier was not present.

Due to the perception of FGM/C as a highly sensitive topic, some health providers avoided discussion about it as they did not want to offend or stigmatise women/girls or jeopardise their relationships. Hence, in order to be culturally sensitive or non-judgemental, some health providers would not ask about FGM/C at all, assuming that women would raise the issue if there was a problem. This could lead to the topic never being discussed, hence opportunities for timely interventions could be missed. Other health providers felt that it was best to ask openly and directly about FGM/C [78]. Direct communication was considered easier if it was seen to be part of a routine process within a consultation (e.g. a standard question on an assessment form) [79, 84, 91, 99, 100]. Health providers also noted that good communication relied on being able to develop a trusting relationships with clients–which in turn was facilitated by practises such as continuity of care or by having sufficient time in consultations [77, 79, 97, 98, 102]. Experience and training were noted to improve health provider’s confidence in talking about FGM/C [78, 79, 91, 99].

**Descriptive theme 2.3: Women also find FGM/C hard to talk about.** Thirteen studies reported providers’ views that women rarely proactively mentioned FGM/C in the context of a consultation. Providers attributed this to cultural taboos within women’s own societies, feeling ashamed and embarrassed or being fearful of being judged. It was perceived that since women did not talk about FGM/C in the first place, then it was even harder for providers to discuss a topic they themselves did not always fully understand.

#### Analytical theme 3: Encountering the ‘other’ in clinical practice: Negotiating cultural dissonance and achieving cultural understanding within healthcare relationships

This theme highlights the healthcare encounters between professionals and FGM/C-affected women as sites where different cultures and values met and needed to be negotiated. Cross-cultural encounters evoked emotional reactions that affected professional behaviour and interpersonal processes, as well as requiring adjustments to practical aspects of service provision (e.g. responding to women’s preferences for a female provider or interpreter). Achieving cultural understanding and mutual respect led to culturally appropriate care provision. When this was not achieved, quality of care was compromised.

**Descriptive theme 3.1: Attitudes towards FGM/C: mixed emotions.** This theme was reported by 17 studies, and describes provider views and attitudes around FGM/C. Many providers expressed strong emotions around FGM/C, including shock, disgust and horror. FGM/C was seen as an alien and negative cultural practice, with providers describing women’s bodies after FGM/C as different, not ‘normal’—mutilated. At the same time, providers (especially midwives and nurses), expressed great empathy and sympathy for affected women, and tried to be supportive [75, 76, 85]. Women were often talked about as victims of cultures that were violent, barbaric and patriarchal. Some providers mentioned a struggle to maintain their professionalism around this topic, having to hide their feelings of horror when they first encountered FGM/C. They also expressed anger towards the ‘other’ society and particularly towards the men in that society.

**Descriptive theme 3.2: Cultural dissonance–control and resistance in clinical encounters.** This theme, reported in 20 studies, refers to cultural differences between health providers and patients, and to cultural assumptions and stereotypes that may exist and that may affect care by undermining trust and communication. Of note is that in many of the papers, providers seemed to be referring to their experiences with Somali patients in particular.

In some studies, providers reflected that they, as health professionals, sometimes held stereotyped views, particularly of Somali FGM/C-affected women which may affect their care (for example, assumptions that Somali women were ‘tough’ and did not require or want pain relief during labour), hence failing to see women as individuals, especially when there was a language barrier [78, 79, 92–94, 98, 101–104]. Conversely, there was a widespread sense that Somali patients in particular did not trust the western healthcare system and held fears and assumptions about what may happen to them [103].

The studies indicated that key areas of cultural difference manifested in differences in views around pain behaviour, C-section, episiotomies/deinfibulation, vaginal examinations (e.g. pap smears) [102] and other clinical interventions. The C-section was a particular site of contestation and resistance, with providers across multiple studies reporting women’s and families’ resistance to this procedure even when medically indicated [78, 88, 92, 93, 98, 101–104]. Many studies reported examples where providers became perplexed and frustrated that patients did not follow their advice. Such encounters could become highly charged with different stakeholders (providers, women, families) all seeking to exert control over the clinical situation. The studies provided several examples of situations which were not well managed, leading to resistance, misunderstandings, miscommunication and poor clinical outcomes (such as women being denied pain relief, enduring traumatic births, refusing to have a C-section where it was indicated, or where providers imposed a C-section without proper informed consent and explanation) [88, 90, 101, 103, 104]. However, some studies also provided examples showing that, when care was taken to build trust and mutual understanding, clinical situations could become easier to manage [90, 102].

**Descriptive theme 3.3: Acknowledging the role of the family.** This theme, reported by 15 studies, refers to the significant role of the family in influencing women’s decision making and actions–in contrast to ‘western’ patients where care decisions are more often made on an individual basis. Providers recognised that women’s healthcare decisions and behaviours were strongly influenced by their husbands and wider family [77, 82, 85, 92–95, 101]. This was, at times, experienced as frustrating, but involvement of the family was seen as important for effective communication and effective care. With respect to FGM/C specifically, family involvement was seen to be particularly crucial in decision making in relation to deinfibulation timing [85, 100] and reinfibulation [85, 88, 94, 95].

**Descriptive theme 3.4: Gender of the provider.** This theme, reported in eight studies, relates to how the provider’s gender influences the care of women with FGM/C. The studies reported a strong perception that women from FGM/C affected communities preferred to be seen by a female health professional. The gender of the provider was perceived to affect women’s willingness to seek help, to talk openly to the practitioner and finally, to be examined (so that even if the problem had been disclosed, a patient may refuse further examination if the practitioner was male) [82]. Knowing patients’ discomfort with male providers could also make male professionals reluctant to raise the issue of FGM/C at all [95].

**Descriptive theme 3.5: Crossing the cultural divide–strategies and elements of culturally sensitive care.** This theme, reported in 23 studies, refers to strategies and outlooks that were adopted by providers in their endeavours to ‘cross cultural divides’. It was evident that many providers tried hard to be culturally sensitive and viewed this as an essential aspect of their professional identity and practice. Hence, many papers described strategies and approaches that providers felt were important to build good relationships with FGM/C-affected women and thus to provide appropriate care. In addition, professionals noted the importance of understanding FGM/C within the context of women’s wider non-healthcare needs and culture (i.e. being person centred as well as ‘culturally’ sensitive), and having time to build rapport [78, 79, 83, 84, 86, 95, 103, 104]. Culturally sensitive care was particularly discussed by midwives and nurses.

#### Analytical theme 4: Identifying FGM/C: Hit and miss

A key review finding was that appropriate identification of FGM/C is a ‘hit and miss’ process dependent upon individual provider behaviour as well as the existence (or not) of relevant organisational systems and processes.

**Descriptive theme 4.1: Presentation and help seeking.** In this theme, providers in 15 studies reported a range of experiences in how women presented to services and how they might identify FGM/C. Some, especially in lower prevalence areas, felt they had encountered it only rarely [75, 77, 79, 80, 83, 84, 86, 87, 93, 94], whereas others who worked in locations with high migrant populations were more familiar [76, 78, 82, 84]. There was a feeling that women primarily only sought medical help once symptomatic or for pregnancy. Even then, it was felt that women may not make a connection between their symptoms (e.g. frequent urinary tract infections) and their FGM/C [77, 86, 95, 96, 104]. As a result, given that many medical procedures and consultations do not require physical examination, providers felt that FGM/C may not be identified at all unless they specifically asked [77, 84, 86, 95, 96, 104]. Likewise, some studies noted that it could be particularly hard to identify type I or type II FGM/C during examination and that this might account for apparently ‘low’ rates of identification [84, 85, 93–95]. A few studies mentioned a view that younger women may be becoming more ‘bold’ in seeking out care to alleviate symptoms [75, 79, 95]. Hence, the studies consistently noted that it was the provider who needed to take the initiative to ask about FGM/C and that women may be reluctant to raise the subject of FGM/C themselves [93, 94, 96]. However, there were exceptions, namely when there were specialist services or experienced providers available, who women might seek out themselves via personal recommendations or upon the advice of friends/family [77, 84–86].

The majority of papers contributing to this theme focused on maternity contexts. It is unclear to what extent providers in other settings saw it as their responsibility to consider FGM/C. However, a study of school nurses in Sweden reported that in-school nurse education sessions proved to be a useful way of encouraging girls to come forward to discuss their FGM/C [79]. Likewise, a study with health visitors in England also showed that they were able to identify and support women with FGM/C through their routine interactions outside of the maternity context [99]. In contrast, GPs in an Australian study did not see discussing FGM/C as part of their role or as necessary unless there was a medical problem [84].

**Descriptive theme 4.2: Practices and processes around identifying FGM/C.** Twenty studies across a range of countries indicated that the presence of organisational and system-related mechanisms to identify FGM/C was variable, and that practices could be inconsistent and uncoordinated [100]. There was a general agreement amongst providers that, especially for pregnant women, FGM/C should be identified antenatally where possible but this did not always happen [84, 93, 94, 100]. As indicated in the previous theme, a key barrier to early and appropriate identification of FGM/C was that providers did not ask about FGM/C. However, several studies reported that organisational systems and processes were often not adequately set up to prompt them to ask or to ensure that follow up would then occur. Organisational and system-related barriers to FGM/C identification were associated with: (i) the existence (or not) of clear guidelines, procedures, referral pathways [75, 77, 80, 82, 84, 85, 90, 93, 94, 98], and record keeping processes [77, 80, 85, 90, 93, 98, 100], (ii) clarity of roles and responsibilities, and (iii) to the need for communication/coordination between organisational units and professional groups [77, 90, 93, 95, 101]. For example, some studies reported a confusion between community clinics and hospital in-patient centres over whose responsibility it was to record FGM/C and to provide counselling and care planning [90, 93]. As a result, examinations of women and care planning might not happen [93]. Other studies reported a lack of record keeping [77, 80, 85, 90, 93, 98, 100] so that FGM/C failed to be recorded on the relevant medical notes, and then failed to be discussed further during subsequent consultations [103]. For pregnant women, this could lead to situations where women arrived at the labour ward with no prior counselling regarding their FGM/C [85, 103]. However, even when procedures were in place, one study with midwives in Australia identified several reasons why they might not always be followed [85]. These related to lack of time, a feeling that additional reporting created overly burdensome bureaucracy that was hard to fit into existing work routines and a lack of understanding of how the systems worked [85].

#### Analytical theme 5: Clinical management practices: Inconsistent and variable

The review found that clinical management of FGM/C can be variable and inconsistent dependent upon individual provider knowledge and skills, as well as the existence (or not) of relevant clinical guidelines. Whilst some providers expressed confidence in managing clinical interventions around FGM/C, many of the studies identified uncertainty in terms of good practice and highlighted inconsistencies in clinical practice, including with regard to psychological care. The inconsistency was linked to a perceived lack of clinical guidelines, meaning that the care provided to women could be variable, depending on the experience, confidence and preferences of individual practitioners. Many findings contributing to this theme came from studies looking specifically at obstetric care for women with type III FGM/C and to clinical management practices around deinfibulation and reinfibulation.

**Descriptive theme 5.1: Deinfibulation timing.** This theme, reported in eight studies, relates to professionals’ views on the timing of deinfibulation for women with type III FGM/C. The majority of providers felt that women preferred deinfibulation to be done in the second stage of labour (rather than antenatally) to avoid having to be cut twice. In contrast, most providers would have preferred to undertake deinfibulation antenatally (although some felt this might be an unnecessary additional trauma for the woman), but the usual practise seemed to be deinfibulation in the second stage of labour, as the head is crowning [82, 84, 85, 90, 93–95, 100]. In studies specifically of midwives, it was emphasised that, ideally, decision making around deinfibulation should be a ‘shared’ process occurring as a result of discussions and counselling between providers and women during the antenatal period [82, 85, 100], however, this did not always happen [84, 90, 93, 94, 100]. In some studies, providers noted that women’s decisions around deinfibulation timing were influenced by family members, especially older women [85, 100].

**Descriptive theme 5.2: Deinfibulation practice.** With regards to deinfibulation, findings reported from 21 studies revealed that providers’ confidence, competence and experience around the procedure varied considerably depending upon the professional group, training and setting. Hence, the care that women received could be variable depending on whose care they happened to fall under. Several studies noted that midwives in particular could be anxious and uncertain about deinfibulation [76, 78, 82–85, 94, 98, 101]. Several studies identified inconsistencies in medical doctors’ practice regarding deinfibulation [85, 90, 93–95], and three studies reported that doctors were sometimes quick to decide to recommend a C-section, due to uncertainty or lack of experience, especially when this was compounded by difficulties in being able to undertake routine monitoring in labour [90, 93, 101].

One study highlighted how decision making around deinfibulation could also be influenced by practitioners’ assumptions about women’s preferences based on cultural stereotypes rather than discussing the issue with them [88]. In this study, conducted amongst midwives in Norway, midwives knew that infibulation was of cultural importance to the Somali community. They therefore assumed that Somali women wanted to remain infibulated. Hence, in an attempt to be culturally sensitive, some midwives reported undertaking extensive episiotomies rather than performing deinfibulation.

Five studies of midwives noted that it was important to provide women with counselling and in-depth information after deinfibulation so they could adjust to an altered body image and altered physical sensations [76, 82, 85, 90, 100].

**Descriptive theme 5.3: Reinfibulation ambivalence.** Findings from 11 studies demonstrated that professionals’ attitudes towards reinfibulation could be ambivalent, and that their practise relating to reinfibulation was variable. In general, health practitioners were clear that reinfibulation was illegal and should not be performed. However, in three studies, practitioners reported having undertaken some degree of reinfibulation [88, 90, 95] (but it should be noted that these were all old studies and may no longer reflect current practice).

Although knowing that reinfibulation was illegal, some midwives felt unsure about the detail of FGM/C and the law and were therefore unsure about how to best explain the issue in a culturally sensitive way when requested [81, 82]. Several studies reported that practitioners sometimes felt ambivalence and a moral dilemma about refusing to reinfibulate a client if she was clearly requesting it, and felt that it should be done. Refusing a patient’s request was seen to contradict the principles of person-centred care. These practitioners worried that refusing reinfibulation may cause harm in terms of causing marital problems for women and their husbands or may cause women distress in terms of adjusting to an altered body image [78, 81–83, 85, 88–90, 93–95].

Some studies noted that where there was clarity on the law, it was perceived to be a helpful way of supporting practitioners in explaining why they were refusing reinfibulation [94, 100].

Several studies highlighted the important role of husbands in decision making around reinfibulation (this varied in terms of requesting it or not wanting it for their wives) and, where appropriate, of the benefit of involving them in women’s care [85, 88, 92, 94].

**Descriptive theme 5.4: Need for guidelines around deinfibulation/reinfibulation.** Fifteen studies reported a lack of (or lack of awareness of) clinical guidelines. In several studies, practitioners noted that guidelines would be useful in establishing clarity and consistency of practice around deinfibulation timing, procedure and reinfibulation [84, 100].

**Descriptive theme 5.5: Psychological issues.** Twelve studies reported providers’ experiences that FGM/C could evoke painful and difficult psychological responses in women at any life stage, and some practitioners emphasised the importance of offering counselling and psychological support [75, 76, 82, 85]. Interestingly, these aspects were mainly mentioned by nurses/midwives (and the studies throughout the review in general gave most prominence to physical rather than psychological aspects of women’s care).

Clinical interventions in particular were recognised as potentially traumatic for women in terms of inducing flashbacks from the original procedure, heightening pain and, in the context of birth, sometimes leading to difficult deliveries. In a maternity context, several studies noted a need to be extra attentive to information giving and counselling in the antenatal period and to pain management during labour and perineal suturing to try and mitigate emotional distress [76, 82, 85, 86, 88, 90, 98]. However, dealing with these situations could also be emotionally challenging for the providers [82, 85, 88, 90].

#### Analytical theme 6: Optimal service development

This theme relates to service development and perceived service gaps related to FGM/C prevention and care. The studies identified a perceived need for service development in three inter-linked areas: (i) community engagement, (ii) prevention, and (iii) specialist service provision. The studies identified variable engagement of providers in addressing FGM/C prevention on an individual level, but there was strong support for the development of specialist holistically focused services that would cover FGM/C care as well as prevention. Providers saw community engagement as essential both for prevention as well as for raising awareness of, and trust in, services.

**Descriptive theme 6.1: Providers’ role in prevention.** Apart from school nurses whose role explicitly encompasses safeguarding and sexual health [76, 79, 89], relatively few studies reported practitioners addressing prevention as part of their FGM/C-related care, although several studies reported a view that this *should* be part of any practitioners’ role, including GPs’ [76, 78, 79, 85, 86, 90, 95]. As a result, prevention discussions appeared to take place in an *ad-hoc* way (dependent on individual providers) rather than as a routinized aspect of care. This theme was reported in 13 studies. Some key barriers to initiating prevention discussions in a clinical setting were identified in six studies (but several of these are now rather old) [78, 85, 90, 92, 94, 95]. These included: lack of time, inappropriate timing (referring to the difficulty of having a prevention discussion when a woman was in labour or had just given birth), feeling that the prevention discussion is someone else’s role, not having enough knowledge or confidence, feeling unsure if women’s responses could be trusted, lack of privacy (e.g. if family members were present) and language barriers.

School nurses did report having discussions with young people about prevention although it was described as a sensitive and difficult topic to address [76, 79, 89]. Nurses in one study reported that discussions were easier if it was possible to speak to the young people alone [79]. One study highlighted that school health practitioners were perhaps overly focused on discussing the clinical or legal aspects of FGM/C and a different approach might be to address sexual/relational concerns and impacts with young people and that this approach might engage them more [89].

**Descriptive theme 6.2: Community engagement and education.** This theme, reported by 11 studies, refers to provider views on community engagement and education. Practitioners identified a need for greater education and awareness raising amongst affected communities, both on FGM/C itself and associated services but also on prevention. They suggested this should include men/boys as well as women/girls. Providers also identified a need for better information resources for communities [76, 79, 85, 95]. Practitioners suggested that community engagement needed to be built on relationships of trust and concern for wider community needs (not just FGM/C) and some suggested that community outreach/liaison roles might be beneficial [83–85].

**Descriptive theme 6.3: Specialist services.** This theme refers to a perceived need for specialist services. Nine studies identified that providers valued and recommended having specialist centres for FGM/C management where women could be referred and where expertise, advice and training could be accessed [87]. Such specialist centres were seen as a particularly important potential link to communities, in terms of building trust and working together with community outreach workers and trained interpreters [85, 91]. The need for additional counselling services was also highlighted by some studies [76, 85, 95].

Having access to specialist centres was seen as particularly important for practitioners in low prevalence or rural areas which would also go some way to addressing equity of service provision [83, 84, 87, 100].

## Confidence in the review findings

The CERQual assessment of confidence in the review findings graded eight review findings (descriptive themes) as ‘high confidence’, 11 as ‘moderate confidence’ and one as ‘low confidence’. The main causes for downgrading of a review finding were due to concerns related to methodological limitations and coherence (e.g. in some cases not all studies reported fully on all aspects of a review finding). In addition, several findings had moderate concerns related to ‘adequacy’ in terms of having relatively ‘thin’ data contributing to a finding. However, most findings were relevant across professional group, contexts and countries. See Table 3 for the final CERQual assessment for each theme and S8 Table for the full details of the CERQual evaluation.

## Discussion and recommendations: Working across cultural divides

There has been much research documenting the challenges faced by health providers in destination countries in delivering healthcare to migrant populations (e.g. language barriers, cultural differences, lack of time to manage complex patient needs) [106–111]. This review has highlighted all of these issues, but has shown that, when delivering care related to FGM/C, these challenges can all become exacerbated. This is primarily due to lack of familiarity with FGM/C and the cultural sensitivities associated with FGM/C leading to silence, stigma and inaction. The review suggests that cultural divides manifest themselves at two distinct but inter-linked levels: (i) individual provider level (e.g. in uncertainty in how to understand, talk about or manage FGM/C) but also, (ii) at a system and service level (e.g. in models of care that do not embed FGM/C-related issues into routine practice). The discussion below considers how cultural divides are exacerbated and can be addressed within these two levels.

### Provider-level challenges and strategies for action

The reviews confirm findings from other studies that show an unmet training need for knowledge and skill development in all aspects of FGM/C-related care and in the development of cultural competence [34–36, 112–117]. This appears to apply to all cadres of health professional [118–123]. WHO has recently released a clinical management handbook that provides excellent comprehensive guidance for practitioners [27]. In addition, many countries are undertaking training initiatives for their health professionals [23]. However, it is currently unclear to what extent knowledge may have improved as a result of recent initiatives or to what extent FGM/C is now embedded within pre- or post-registration training curricula. This is an area in need of future research [124]. Although now somewhat out of date, a UK survey conducted in 2012 by the Royal College of Midwives amongst 1,756 midwives suggests that some improvement may still be needed. The results showed that the majority of participants did not know to where/whom they should refer a woman with FGM/C, and only 15.3% had attended a training session [125]. Likewise, a 2015 survey amongst a mixed sample of 157 doctors and midwives at a hospital in London with a high prevalence of FGM/C, found that 71.4% felt they would benefit from further training on the subject and only 21% of respondents stated that they would feel comfortable discussing FGM/C with patients. The majority of healthcare professionals (65.3%) had not read any guidelines relating to FGM/C [126]. Other recent studies in the UK, Spain and Australia provide further evidence that substantive training needs remain [112, 121, 127, 128].

The review findings suggest that additional training seems to be particularly required in relation to communication around FGM/C, awareness of women’s psychological needs and management of clinical interventions (particularly deinfibulation where the review suggests that competency can be highly variable). In addition, the review showed that management of FGM/C could be stressful for some health providers, especially when they feel they lack clinical competence and when cross-cultural misunderstandings arise. Similar findings have been reported in other contexts [115]. Hence, staff may also benefit from opportunities to discuss FGM/C-related issues face to face in a supportive environment with experts, including FGM/C survivors [120]. Furthermore, as with any skill, it is important for professionals who may be called upon to undertake deinfibulation, to have adequate training that extends beyond classroom or e-learning and includes a skills-based element. Recent systematic reviews show that the evidence on the most effective approaches to training around FGM/C is unclear, and would benefit from further research [119, 129]. One study by Jacoby et al [118], however, showed a clear increase in knowledge, confidence and cultural competence among midwives in the USA following a training programme that included didactic teaching, case studies, a cultural roundtable discussion, and a hands-on skills session on deinfibulation and repair.

At an inter-personal level, the review findings point to more positive care experiences when practitioners were able to build a trusting relationship with women to engage in shared decision making and to offer person-centred care. Most healthcare practitioners are trained in these latter approaches, but the review supports existing evidence showing how challenging it can be to implement these when there is limited time and poor communication [130, 131]. The reviews suggested that continuity of care could be a key strategy for helping to build trusting relationships in order to provide better quality care [132–134].

### Service and system level challenges and strategies for action

This review has also demonstrated that, in addition to provider characteristics, there are important service and system issues that influence the provision of FGM/C-related care. Given that many countries are making efforts to develop and implement FGM/C-related guidelines, in order to support effective implementation, it is imperative that these wider issues are understood. Key issues highlighted by the review relate to: (i) language barriers, (ii) care pathways and protocols, and, (iii) service configuration.

The review findings concur with a large body of literature that shows how language barriers negatively affect the care of migrant groups and calls for service innovations to address these [135]. The review showed that a common strategy to address language barriers was use of interpreters, either formal or informal, but, in line with other evidence in this area, both these strategies could also be potentially problematic [136, 137]. Problems with interpretation can potentially be addressed by increased training and availability of interpreters [138]. However, in the context of FGM/C, due to the sensitivity of the topic, one may anticipate that problems may remain. An alternative approach suggested by the health professionals in the review was to develop a community advocate or liaison role, with an expanded remit, to act not just as an interpreter, but as a cultural mediator who could raise awareness, befriend women, signpost to services and act as an advocate for their rights. Such approaches have been positively evaluated in other settings [139–143], but need further exploration with reference to FGM/C.

In terms of care pathways and protocols, the review has shown that in many cases, professionals reported a lack of knowledge of guidelines or protocols, both for identifying FGM/C and for managing FGM/C. These findings are supported by evidence from a wider global context [34, 35, 116]. In relation to identifying FGM/C, the review showed that providers often missed opportunities to discuss FGM/C–partly due to lack of knowledge and confidence, but also due to the fact that it may not have been considered to be a routine part of assessment or history taking processes. Professionals noted that guidelines would help them to ask the key questions at the right time, and subsequently to be confident about care management, especially deinfibulation and dealing with requests for reinfibulation. The review suggests therefore, that it may be helpful for questions about FGM/C to be routinized within certain settings, and for clear guidelines to be developed within different services [30]. Similar approaches have been very successfully utilised to address other health topics that communities and practitioners have traditionally felt uncomfortable with discussing–such as HIV testing [144, 145] or domestic violence [146, 147] for example.

The review also aimed to illuminate the question of why, even when guidelines did exist, they may not always be followed. The review was unable to identify a great deal of in-depth information in relation to this question, as we did not find any process evaluations of interventions. However, the evidence was able to offer limited insights of issues that may potentially affect implementation. These include lack of clarity over roles and responsibilities, increased workload burden of reporting, unclear systems of recording FGM/C and unclear follow up care pathways.

There is a wide body of evidence exploring barriers to guideline and intervention implementation [148–151] that falls broadly within the field of implementation science [152]. Future research or evaluation on FGM/C-related guideline implementation would benefit from utilising an established framework to analyse, understand and address potential barriers further. This may be particularly important in settings such as the UK, where recently introduced policies of mandatory reporting and recording have introduced a layer of complexity into an already sensitive topic area and a layer of bureaucracy into consultation processes that are already stretched for time (especially when there are language barriers to negotiate) [153].

In terms of configuration of services for FGM/C, the review highlighted two issues. One related to the availability and accessibility of specialist expertise. The second related to the nature of services provided (i.e. the model of care). Providers suggested that FGM/C-related care should be delivered by specialists or should be supported by easily accessible specialist advice. However, the review highlighted that the availability of specialist expertise in areas of low prevalence or rural areas could be challenging. The review did not identify any qualitative studies evaluating models of care. However, stakeholders at the national engagement event suggested that a hub and spoke model or mobile clinics might be a way forward in low prevalence areas.

The review findings also suggest that specialist services need to extend beyond provision of medical care to encompass a more holistic model, including counselling/psychotherapy and specialist sexual health services. These findings are supported by the wider evidence [22, 154–158]. Several such models already exist [22] (see also specific examples from England [159, 160]). All include a strong element of community engagement and partnership [161]. Further mapping and evaluation of models of care would be beneficial to understand better their differential impact on accessibility, outcomes, cost and patient satisfaction.

## Strengths and limitations

This was an extremely comprehensive review, covering all OECD countries, all languages and extensive grey literature sources. In addition, the review expanded a conventional focus on maternity care to include FGM/C-related care across all clinical contexts and all cadres of health professional. In doing so, it was able to shed particular light on the challenges in discussing and identifying FGM/C in non-maternity settings and the need for joined-up care pathways. Another key strength of the review process is that it has been informed by strong community involvement and input from a multi-disciplinary expert advisory group at every stage.

However, the limited evidence on non-maternity settings has also highlighted a need for more research on the views, experiences and practices of professionals in primary care or other non-obstetric settings (e.g. sexual health, health visiting, school nursing, general practice). Other limitations of the review are that there was very limited evidence on providers’ practices around prevention and safeguarding and, as highlighted above, no research on factors that facilitate or hinder implementation of, or adherence to, guidelines around FGM/C identification and record keeping.

## Conclusions

Due to the growing diversity of populations in migrant-destination countries, all health professionals are called upon to develop cultural awareness, cultural sensitivity and cultural competence [6, 107, 108]. This review has demonstrated some of the challenges inherent in crossing cultural divides especially when dealing with highly sensitive topics such as FGM/C. The result is silence, stigma and uncertainty. The review has also shown that, with respect to FGM/C, although health professionals may be well intentioned, significant gaps remain in their knowledge and skills which adversely affect the timely identification, recording and management of FGM/C. Finally, this review also demonstrates that there is a need for health services and systems to innovate and adapt to create environments and processes that can support professionals to deliver culturally appropriate care. The review suggests that optimal service configurations for the management of FGM/C require clear guidelines, protocols, access to specialist support, strategies to address language barriers, and strategies to engage and involve communities.

## Supporting information

S1 Table Full Ovid Medline search strategy. (DOCX)

S2 Table Resources/Databases searched. (DOCX)

S3 Table Excluded studies with reasons. (DOCX)

S4 Table Operational criteria of study relevance. (DOCX)

S5 Table Theme matrix. (DOCX)

S6 Table Full table of study characteristics. (DOCX)

S7 Table Full table of assessment of methodology. (DOCX)

S8 Table CERQUAL assessment and summary of findings. (DOCX)

S9 Table Completed PRISMA checklist. (DOC)
