# Supplementary material for: Crossing cultural divides: A qualitative systematic review of factors influencing the provision of healthcare related to female genital mutilation from the perspective of health professionals
Source: PLoS One. 2019 Mar 4;14(3):e0211829. doi: 10.1371/journal.pone.0211829 (PMC6398829; doi:10.1371/journal.pone.0211829)
Supplement: S1 Table — (DOCX) [file pone.0211829.s001.docx]

# Supplementary File 1: Medline Search Strategy

Ovid Medline In-Process & Other Non-indexed Citations and Ovid Medline 1946 to present – run on 04/08/2017

| **Search statement** | **Search terms** | **Hits** | **Notes on strategy** |
| --- | --- | --- | --- |
| 1 | exp Circumcision, Female/ or (exp Genitalia, Female/ and exp Medicine, Traditional/) | 1367 |  |
| 2 | ("female genital mutilation" or "female circumcision" or "female genital cutting" or "traditional female genital surgery" or infibulat* or reinfibulat* or defibulat* or clitoridect*).mp. | 1509 |  |
| 3 | ((((ritual* or traditional* or ceremon* or sociali#ation*) adj3 (practic* or cut or cutting or surg*)) or circumcis* or excis*) adj3 (female* or wom#n or girl* or child* or adolescen*)).mp. | 2924 |  |
| 4 | (FGM or FGC or TFGS or FGMC or "FGM/C").mp. | 1133 |  |
| 5 | (thara or tahar or khitan or khifad or khafad or megrez or absum or mekhnishab or kutairi or ibi or ugwu or sunna or bondo or sonde or gudiniin or halalays or qodiin or tahoor or bagne or gadja or ganza or "fanadu di mindjer" or niaka or kuyango or "musolula karoola").mp. not (thara or tahar or khitan or khifad or khafad or megrez or absum or mekhnishab or kutairi or ibi or ugwu or sunna or bondo or sonde or gudiniin or halalays or qodiin or tahoor or bagne or gadja or ganza or "fanadu di mindjer" or niaka or kuyango or "musolula karoola").au,in. | 1650 | Expanded terms to include country of origin terminology as described in Department of Health guidance.^121^ |
| 6 | 4 or 5 | 2779 |  |
| 7 | limit 6 to female | 1200 | In order to exclude irrelevant records from terminology from other disciplines, or male circumcision related hits |
| 8 | ((FGM or FGC or TFGS or FGMC or "FGM/C" or thara or tahar or khitan or khifad or khafad or megrez or absum or mekhnishab or kutairi or ibi or ugwu or sunna or bondo or sonde or gudiniin or halalays or qodiin or tahoor or bagne or gadja or ganza or "fanadu di mindjer" or niaka or kuyango or "musolula karoola") adj3 (female* or wom#n or girl* or child* or adolescen*)).mp. | 507 | Adjacency to “female” terms to complement “limit to female” hit set |
| 9 | (egypt* or sudan* or ethiopi* or somali* or djibouti* or eritrea* or burkin* or guinea* or sierra leone or leonean* or mauritan* or mali or malian*).ti,ab. | 151913 | Country of origin used as proxy for FGM/C terms – as FGM/C is so common, inference is that women from these countries will fit an FGM/C profile even without the term being explicitly mentioned |
| 10 | limit 9 to female | 46657 |  |
| 11 | ((egypt* or sudan* or ethiopi* or somali* or djibouti* or eritrea* or burkin* or guinea* or sierra leone or leonean* or mauritan* or mali or malian*) adj3 (female* or wom#n or girl* or child* or adolescen*)).mp. | 5441 |  |
| 12 | 1 or 2 or 3 or 7 or 8 or 10 or 11 | 51901 | All women with FGM/C |
| 13 | "Emigrants and Immigrants"/ or Refugees/ or "Transients and Migrants"/ or "Emigration and Immigration"/ | 45756 |  |
| 14 | (immigrant* or immigrat* or emigrant* or emigrat* or emigre* or expat* or (ex adj pat*) or transients or newcomer* or (new adj comer*) or alien* or incomer* or (in adj comer*)).mp. | 79630 |  |
| 15 | ((immigrant* or immigrat* or emigrant* or emigrat* or emigre* or expat* or (ex adj pat*) or transients or newcomer* or (new adj comer*) or alien* or incomer* or (in adj comer*)) adj3 (female* or wom#n or girl* or child* or adolescen*)).mp. | 4382 |  |
| 16 | (refugee* or (asylum adj seek*) or asylee* or (refused adj3 (asylum* or refugee*)) or (displaced adj person*) or exile* or (new adj arrival) or (country adj2 (birth or origin)) or transnational*).mp. | 17539 |  |
| 17 | ((refugee* or (asylum adj seek*) or asylee* or (refused adj3 (asylum* or refugee*)) or (displaced adj person*) or exile* or (new adj arrival) or (country adj2 (birth or origin)) or transnational*) adj3 (female* or wom#n or girl* or child* or adolescen*)).mp. | 1462 |  |
| 18 | (foreigner* or (foreign adj (born or citizen* or national* or origin*)) or (non adj (citizen* or native*)) or ((adoptive or naturali#ed) adj (citizen* or resident*)) or overstay* or trafficked or "spousal migrant*").mp | 11442 |  |
| 19 | ((foreigner* or (foreign adj (born or citizen* or national* or origin*)) or (non adj (citizen* or native*)) or ((adoptive or naturali#ed) adj (citizen* or resident*)) or overstay* or trafficked or "spousal migrant*") adj3 (female* or wom#n or girl* or child* or adolescen*)).mp. | 753 |  |
| 20 | (((established or long-term or "first generation*" or new* or recent* or current*) adj3 (migrant* or migrat* or immigrant* or immigrat* or emigrant* or emigrat* or emigre* or expat* or (ex adj pat*) or transient* or alien*)) or newcomer* or (new adj comer*) or incomer* or (in adj comer*) or ((international or overseas or foreign) adj2 (student* or employee* or worker*))).mp. | 18239 |  |
| 21 | ((((established or long-term or "first generation*" or new* or recent* or current*) adj3 (migrant* or migrat* or immigrant* or immigrat* or emigrant* or emigrat* or emigre* or expat* or (ex adj pat*) or transient* or alien*)) or newcomer* or (new adj comer*) or incomer* or (in adj comer*) or ((international or overseas or foreign) adj2 (student* or employee* or worker*))) adj3 (female* or wom#n or girl* or child* or adolescen*)).mp. | 559 |  |
| 22 | ("non-UK-born" or "born outside the UK" or "length of residence in the UK" or (("not lawful*" or "not legal*" or unlawful* or illegal* or unauthori#ed* or "not authori#ed" or uncertain or insecure or legal* or legitimate* or permit* or visa* or irregular* or refused or undocumented) adj3 (residen* or student* or worker* or employee* or unemployed or immigrant* or imigrat* or migrant* or migrat*))).mp. | 2887 |  |
| 23 | (("non-UK-born" or "born outside the UK" or "length of residence in the UK" or (("not lawful*" or "not legal*" or unlawful* or illegal* or unauthori#ed* or "not authori#ed" or uncertain or insecure or legal* or legitimate* or permit* or visa* or irregular* or refused or undocumented) adj3 (residen* or student* or worker* or employee* or unemployed or immigrant* or imigrat* or migrant* or migrat*))) adj3 (female* or wom#n or girl* or child* or adolescen*)).mp. | 136 |  |
| 24 | exp Vulnerable Populations/ | 8275 |  |
| 25 | ((vulnerab* or disadvantag* or minorit*) adj3 (individ* or person* or people* or population* or communit* or group*)).ti,ab. | 33112 |  |
| 26 | ((vulnerab* or disadvantag* or minorit*) adj3 (individ* or person* or people* or population* or communit* or group*) adj3 (female* or wom#n or girl* or child* or adolescen*)).mp. | 1756 |  |
| 27 | exp Ethnic Groups/ | 139042 |  |
| 28 | (ethnic* or ethno* or race or racial*).mp. | 248038 |  |
| 29 | ("Black and Minority Ethnic" or "Black & Minority ethnic" or BME or BAME or black african*).mp. | 3756 |  |
| 30 | ((ethnic* or ethno* or race or racial* or "Black and Minority Ethnic" or "Black & Minority ethnic" or BME or BAME or black african*) adj3 (female* or wom#n or girl* or child* or adolescen*)).mp. | 9504 |  |
| 31 | exp african continental ancestry group/ or exp asian continental ancestry group/ | 136483 |  |
| 32 | (africa* or "middle east*" or asia* or iran* or iraq* or israel* or oman* or "united arab emirat*" or UAE or "saudi arabia*" or palestin* or india* or indonesia* or malaysia* or pakistan*).ti,ab. | 520081 |  |
| 33 | (egypt* or yemen* or sudan* or ethiopi* or somali* or djibouti* or eritrea* or kenya* or uganda* or tanzania* or camero?n* or chad* or niger* or benin* or togo* or ghana* or burkin* or ivory coast* or cote d'ivoire or liberia* or guinea* or sierra leone or leonean* or gambia* or senegal* or mauritan* or mali or malian* or "sara subgroup" or ngama).ti,ab. | 259558 |  |
| 34 | ((africa* or "middle east*" or asia* or iran* or iraq* or israel* or oman* or "united arab emirat*" or UAE or "saudi arabia*" or palestin* or india* or indonesia* or malaysia* or pakistan* or egypt* or yemen* or sudan* or ethiopi* or somali* or djibouti* or eritrea* or kenya* or uganda* or tanzania* or camero?n* or chad* or niger* or benin* or togo* or ghana* or burkin* or ivory coast* or cote d'ivoire or liberia* or guinea* or sierra leone or leonean* or gambia* or senegal* or mauritan* or mali or malian* or "sara subgroup" or ngama) adj3 (female* or wom#n or girl* or child* or adolescen*)).mp. | 58342 |  |
| 35 | 13 or 14 or 16 or 18 or 20 or 22 or 24 or 25 or 27 or 28 or 29 or 31 or 32 or 33 | 1117829 |  |
| 36 | limit 35 to female | 491179 | Keywords for immigrant populations limited to female (Medline standard limit feature) |
| 37 | (13 or 24 or 27 or 31) and (female* or wom#n or girl* or child* or adolescen*).mp. | 177101 | MeSH Headings for immigrant populations limited by “female” keywords |
| 38 | 15 or 17 or 19 or 21 or 23 or 26 or 30 or 34 or 36 or 37 | 526145 | Terms for immigrant populations, including specific terms for countries of origin where FGM known to be practised, limited to female |
| 39 | exp Community Health Workers/ or exp Volunteers/ | 23517 | All health and allied workers (who may come into contact with women with FGM) |
| 40 | exp Occupational Groups/ | 531564 |  |
| 41 | exp Health Personnel/ | 454091 |  |
| 42 | exp Midwifery/ | 17848 |  |
| 43 | exp General Practitioners/ or exp Physicians/ | 117502 |  |
| 44 | exp Nurses/ or exp Nurses, Community Health/ | 81947 |  |
| 45 | exp Nurses' Aides/ or exp Nursing Staff, Hospital/ or exp Nursing Staff/ | 66494 |  |
| 46 | exp Social Workers/ or exp Police/ | 4605 |  |
| 47 | 39 or 40 or 41 or 42 or 43 or 44 or 45 or 46 | 562187 |  |
| 48 | exp Pregnancy/ or (pregnan* or birth* or childbirth* or matern* or gyn#e* or obstetric* or menstru* or labo#r* or vulv* or vagina* or uter*).ti,ab. | 1391519 | Physical complications or disorders as identified as health impacts of FGM in Department of Health guidance,^121^ already “female” by context of terms – so proxy for FGM. |
| 49 | exp Pregnancy Complications/ or exp Pregnancy High Risk/ | 401801 |  |
| 50 | ((pregnan* or birth* or childbirth* or matern* or gyn#e* or obstetric* or menstru* or labo#r* or vulv* or vagina* or uter*) adj3 (poor* or adverse or complicat* or difficult* or disorder* or dysfunction* or disease* or pain* or risk* or danger* or problem* or issue* or concern* or infect* or inflamm*)).ti,ab. | 137220 |  |
| 51 | exp Obstetric Labor Complications/ | 63618 |  |
| 52 | exp Female Urogenital Diseases/ | 1161123 |  |
| 53 | exp Menstruation Disturbances/ | 27153 |  |
| 54 | exp Genital Diseases, Female/ or exp Vaginal Diseases/ or exp Vulvar Diseases/ | 432325 |  |
| 55 | exp Vaginismus/ or exp Vulvodynia/ or exp Dyspareunia/ or exp Vaginitis/ or exp Vulvovaginitis/ | 14171 |  |
| 56 | exp Pelvic Inflammatory Disease/ | 10694 |  |
| 57 | exp Vaginal Fistula/ or exp Rectovaginal Fistula/ | 4659 |  |
| 58 | exp Genital Diseases, Female/ | 432325 |  |
| 59 | exp Infertility, Female/ | 27170 |  |
| 60 | ((pelvic* or back* or urolog* or urogenit* or urinat* or genit* or abdomin*) adj3 (poor* or adverse or complicat* or difficult* or disorder* or dysfunction* or disease* or pain* or risk* or danger* or problem* or issue* or concern* or infect* or inflamm*) adj3 (female* or wom#n or girl* or child* or adolescen*)).mp. | 29027 |  |
| 61 | exp Urologic Diseases/ | 698515 | Physical complications or disorders as identified as health impacts of FGM in Department of Health guidance,^121^ limited to “female” (female genital diseases, etc.) – so proxy for FGM |
| 62 | exp Pelvic Pain/ | 8163 |  |
| 63 | exp Sexual Dysfunctions, Psychological/ | 24271 |  |
| 64 | exp Pelvic Infection/ | 5409 |  |
| 65 | exp Kidney Diseases/ | 474329 |  |
| 66 | exp Urinary Fistula/ or exp Rectal Fistula/ or exp Urinary Bladder Fistula/ | 11750 |  |
| 67 | 61 or 62 or 63 or 64 or 65 or 66 | 737520 |  |
| 68 | limit 67 to female | 352622 |  |
| 69 | 67 and (female* or wom#n or girl* or child* or adolescen*).mp. | 397916 |  |
| 70 | 48 or 49 or 50 or 51 or 52 or 53 or 54 or 55 or 56 or 57 or 58 or 59 or 60 or 68 or 69 | 2386342 |  |
| 71 | exp Mental Disorders/ | 1122844 |  |
| 72 | exp Depressive Disorder, Major/ or exp Suicide/ or exp Depression/ or exp Suicidal Ideation/ or exp Depressive Disorder/ or exp Bipolar Disorder/ or exp Suicide, Attempted/ | 259964 |  |
| 73 | exp Self-Injurious Behavior/ or exp Substance-Related Disorders/ or exp Social Isolation/ | 329005 |  |
| 74 | exp Sexually Transmitted Diseases, Viral/ or exp Sexually Transmitted Diseases/ | 319070 |  |
| 75 | (anxiet* or anxious* or depress* or self-harm* or self-injur* or suicid*).mp. | 650968 |  |
| 76 | ((anxiet* or anxious* or depress* or self-harm* or self-injur* or suicid*) adj3 (female* or wom#n or girl* or child* or adolescen*)).mp. | 30927 |  |
| 77 | (((sex* or viral* or virus* or bacteria*) adj3 diseas*) or HIV or STD* or AIDS).mp. | 516543 |  |
| 78 | ((((sex* or viral* or virus* or bacteria*) adj3 diseas*) or HIV or STD* or AIDS) adj3 (female* or wom#n or girl* or child* or adolescen*)).mp. | 29597 |  |
| 79 | 71 or 72 or 73 or 74 or 75 or 77 | 2107493 |  |
| 80 | limit 79 to female | 932164 |  |
| 81 | 79 and (female* or wom#n or girl* or child* or adolescen*).mp. | 1098666 |  |
| 82 | 76 or 78 or 80 or 81 | 1098666 | Psychological or STD related impacts of FGM, as above |
| 83 | exp United Kingdom/ or ("united kingdom" or "great britain" or britain or england or scotland or wales or "northern ireland" or british or english or scottish or welsh or "northern irish" or hebrid* or "isle of man").ti,ab,gc,ia,pl,in. | 1167455 |  |
| 84 | (AUSTRALIA* or AUSTRIA* or BELGIUM* or belgian* or CANAD* or CHILE* or CZECH* or DENMARK* or danish* or ESTONIA* or FINLAND or finnish or FRANCE or french).mp. | 641227 |  |
| 85 | (GERMAN* or GREECE or greek* or HUNGAR* or ICELAND* or IRELAND or irish or ISRAEL* or ITALY or italian* or JAPAN* or KOREA* or LATVIA* or LUXEMB* or MEXIC*).mp. | 791022 |  |
| 86 | (NETHERLAND* or holland* or dutch or "low countries" or europe* or "NEW ZEALAND*" or NORWAY or norwegian* or POLAND or polish or PORTUGAL or portuguese).mp. | 597994 |  |
| 87 | (SLOVAK* or SLOVENIA* or SPAIN or spanish or SWED* or SWITZERLAND or swiss or TURK* or "UNITED KINGDOM" or "UK" or britain or british or england or english or scot* or wales or welsh or UNITED STATES or "USA" or america*).mp. | 3770430 |  |
| 88 | 83 or 84 or 85 or 86 or 87 | 5147200 | UK or other OECD countries |
| 89 | randomized controlled trial.pt. | 470445 |  |
| 90 | controlled clinical trial.pt. | 94472 |  |
| 91 | randomized.ab. | 403685 |  |
| 92 | placebo.ab. | 189129 |  |
| 93 | clinical trials as topic.sh. | 187621 |  |
| 94 | randomly.ab. | 280498 |  |
| 95 | trial.ti. | 181309 |  |
| 96 | (animals not (humans and animals)).sh. | 4412254 |  |
| 97 | 89 or 90 or 91 or 92 or 93 or 94 or 95 | 1138847 |  |
| 98 | 97 not 96 | 1049209 | Standard search hedge for randomised clinical trials in Medline – adapted from SIGN.^315^ |
| 99 | 38 and 47 and (70 or 82) | 7952 | Female immigrants AND Health Workers AND (physical or mental disorders) |
| 100 | 47 and 12 | 1144 |  |
| 101 | 12 or 99 or 100 | 59304 | Women with FGM or (Female immigrants AND Health Workers AND (physical or mental disorders)) or (Health workers AND FGM) |
| 102 | 101 and 88 | 12154 | …AND OECD countries |
| 103 | 102 not 98 | 11547 | EXCLUDE RCTs |
| 104 | 103 not 96 | 9261 | EXCLUDE animal studies |
