# Supplementary material for: Crossing cultural divides: A qualitative systematic review of factors influencing the provision of healthcare related to female genital mutilation from the perspective of health professionals
Source: PLoS One. 2019 Mar 4;14(3):e0211829. doi: 10.1371/journal.pone.0211829 (PMC6398829; doi:10.1371/journal.pone.0211829)
Supplement: S2 Table — (DOCX) [file pone.0211829.s002.docx]

**S3 Table of All Resources/Databases Searched**

**Step 1: Initial Searching and Scoping to Refine the Search Strategy**

| **Electronic Resources Searched** | **Date of Search** |
| --- | --- |
| 1. Ovid multifile search (Medline, Embase, PsycINFO) | 10/03/17 |
| 1. POPline (via <http://www.popline.org/>) 1970 to the present | 10/03/17 |
| 1. ProQuest multifile search (ASSIA, Biological Sciences, ERIC, ProQuest Dissertations & Theses A&I, Humanities Index, Index Islamicus, IBSS, MLA, NCJRS, PAIS, PILOTS, Social Services Abstracts, Sociological Abstracts) | 10/04/17 |

**Step 2: Searching Electronic Databases**

| **Electronic Databases Searched** | **Date of Search** |
| --- | --- |
| 1. Applied Social Sciences Index Abstracts (ASSIA) on ProQuest 1987–current | 26/05/17 |
| 1. Ovid MEDLINE 1948– and MEDLINE In-Process and Other Non-Indexed Citations to daily update | 26/07/17 with monthly alert thereafter (cut-off date for included results 31/12/17) |
| 1. Ovid EMBASE 1980–2017 Week 11 | 03/08/17 with monthly alert thereafter (cut-off date for included results 31/12/17) |
| 1. CINAHL Plus with Full Text/EBSCO Host to 2017 | 11/08/17 with monthly alert thereafter (cut-off date for included results 31/12/17) |
| 1. Ovid PsycINFO 1972–March Week 3 2017 | 14/08/17 with monthly alert thereafter (cut-off date for included results 31/12/17) |
| 1. MIDIRS on Ovid 1971 to April 2017 | 18/08/17 |
| 1. HMIC on Ovid 1979 to date | 18/08/17 |
| 1. Thomson Reuters Web of Science 1900–2017. Includes the following:  - Science Citation Index Expanded (SCI-EXPANDED) 1900–2017 - Social Sciences Citation Index (SSCI) 1956–2017 - Conference Proceedings Citation Index - Science (CPCI-S) 1990–2017 - Conference Proceedings Citation Index - Social Science and Humanities (CPCI-SSH) 1990–2017 - Book Citation Index - Science (BKCI-S) 2008–2017 - Book Citation Index - Social Science and Humanities (BKCI-SSH) 2008–2017 - Emerging Sources Citation Index (ESCI) - 2015–2017 | 18/08/17 |

**Step 3: Searching Grey Literature Resources**

1. British Library Ethos ([ethos.bl.uk](file:///C:\Users\jeane\AppData\Local\Packages\microsoft.windowscommunicationsapps_8wekyb3d8bbwe\LocalState\Files\S0\108\ethos.bl.uk))
2. Networked Digital Library of Theses and Dissertations (NDLTD) ([www.ndltd.org](http://www.ndltd.org))
3. NICE (<https://www.nice.org.uk>)
4. Trove – National Library of Australia ([trove.nla.gov.au](file:///C:\Users\jeane\AppData\Local\Packages\microsoft.windowscommunicationsapps_8wekyb3d8bbwe\LocalState\Files\S0\108\trove.nla.gov.au))
5. Open Grey (<http://www.opengrey.eu/>)

**Step 4: Searching and utilizing other sources**

- Google
- Google Scholar
- Experts in the field

**Step 5: Hand searching**

- Hand searching of reference lists (of included studies, of related systematic reviews, of select excluded studies)
