# Supplementary material for: Crossing cultural divides: A qualitative systematic review of factors influencing the provision of healthcare related to female genital mutilation from the perspective of health professionals
Source: PLoS One. 2019 Mar 4;14(3):e0211829. doi: 10.1371/journal.pone.0211829 (PMC6398829; doi:10.1371/journal.pone.0211829)
Supplement: S3 Table — (DOCX) [file pone.0211829.s003.docx]

**Supplementary File 2: Excluded Studies (with Reasons)**

| **No.** | **Study** | **Reason for Exclusion** |
| --- | --- | --- |
|  | Abdel Halim AM. Honorable daughters: The lived experience of circumcised Sudanese women in the United States. PhD Thesis, Ann Arbor: Ohio University, 2003 | Data presented on FGM/C, but no specific focus on FGM/C related healthcare |
|  | Abdel Magied A and Shareef S. Knowledge, perception and attitudes of a sector of female health providers towards FGM - case study: female doctors. *Ahfad Journal*. 2003; 20: 4-17. | Not OECD |
|  | Abdulcadir O, Catania L and Caselli A. Female genital mutilation (FGM): a comparison between the male and the female view. *Int J Gynaecol Obstet*. 2012; 119: S262-S3. | Conference abstract |
|  | Abdullahi, A., Copping, J., Kessel, A., Luck, M. & Bonell, C. (2009) Cervical screening: perceptions and barriers to uptake among Somali women in Camden. *Public Health,* 123(10), 680-5. | Data related to healthcare experiences is from women/girls, not health professionals |
|  | Ahlberg, B.M., Krantz, I., Lindmark, G. & Warsame, M. (2004) ‘It’s only a tradition’: making sense of eradication interventions and the persistence of female ‘circumcision’ within a Swedish context. *Crit Soc Policy,* 24(1), 50-78. | Data related to healthcare experiences is from women/girls, not health professionals |
|  | Ahmed, M. (2005) Attitudes towards FGM among Somali women living in the UK. In *Female Genital Mutilation* (Momoh, C. ed.) Radcliffe, London, pp. 95-117. | Data related to healthcare experiences is from women/girls, not health professionals |
|  | Alizadeh V, Hylander I, Kocturk T and Tornkvist L. Counselling young immigrant women worried about problems related to the protection of "family honour"-From the perspective of midwives and counsellors at youth health clinics. *Scand J Caring Sci*. 2010; 24: 32-40 | Data presented on FGM/C, but no specific focus on FGM/C related healthcare |
|  | Alizadeh V, Tornkvist L and Hylander I. Counselling teenage girls on problems related to the 'protection of family honour' from the perspective of school nurses and counsellors. *Health Soc Care Community*. 2011; 19: 476-84. | Data on FGM/C not presented |
|  | Allag F, Abboud P, Mansour G, Zanardi M and Quereux C. Female genital mutilation. Women's point of view. *Gynecologie Obstetrique Fertilite*. 2001; 29: 824-8. | Data presented on FGM/C, but no specific focus on FGM/C related healthcare |
|  | Ameresekere, M., Borg, R., Frederick, J., Vragovic, O., Saia, K. & Raj, A. (2011) Somali immigrant women's perceptions of cesarean delivery and patient-provider communication surrounding female circumcision and childbirth in the USA. *Int J Gynecol Obstet,* 115(3), 227-30. | Data related to healthcare experiences is from women/girls, not health professionals |
|  | Aquino M, Edge D and Smith DM. Pregnancy as an ideal time for intervention to address the complex needs of black and minority ethnic women: Views of British midwives. *Midwifery*. 2015; 31: 373-9. | Data on FGM/C not presented |
|  | Ariyo D, Ssali R, King-Webb L and Ikpaahindi S. Voices of the community: exploring female genital mutilation in the African community across Greater Manchester. London: AFRUCA, 2015. | Data presented on FGM/C, but no specific focus on FGM/C related healthcare |
|  | Asefaw, F. (2007) [Female Genital Mutilation: A Field Study with Special Consideration of the Background as well as the Health and Psychosexual Consquences for those Affected and their Partners in Eritrea and in Germany] Weibliche Genitalbeschneidung: Eine Feldstudie Unter besonderer Berücksichtigung der Hintergründe Sowie der Gesundheitlichen und Psychosexuellen Folgen für Betroffene und Partner in Eritrea und Deutschland. Humboldt University of Berlin, PhD Thesis, Germany. | Data related to healthcare experiences is from women/girls, not health professionals |
|  | Baldeh, F. (2013) Obstetric Care in Scotland: The Experience of Women who have Undergone Female Genital Mutilation (FGM), Queen Margaret University, MSc Thesis, Edinburgh. | Data related to healthcare experiences is from women/girls, not health professionals |
|  | Ballesteros Meseguer, C., Almansa Martinez, P., Pastor Bravo, M.d.M. & Jimenez Ruiz, I. (2014) [The voice of women subjected to female genital mutilation in the region of Murcia, Spain] La Voz de las mujeres sometidas a mutilacion genital femenina en la region de Murcia. *Gac. Sanit.,* 28(4), 287-91. | Data related to healthcare experiences is from women/girls, not health professionals |
|  | Beck-Karrer C, Levin T and Levin T. Lion women. Conversations with Somalian women and men about female circumcision. *European Journal of Women's Studies*. 1998; 5: 533-6. | Not empirical research |
|  | Beine, K., Fullerton, J., Palinkas, L. & Anders, B. (1995) Conceptions of prenatal care among Somali women in San Diego. *J Nurs Midwifery,* 40(4), 376-81. | Data related to healthcare experiences is from women/girls, not health professionals |
|  | Berggren, V., Bergstrom, S. & Edberg, A.-K. (2006) Being different and vulnerable: experiences of immigrant African women who have been circumcised and sought maternity care in Sweden. *J. Transcult. Nurs.,* 17(1), 50-7. | Data related to healthcare experiences is from women/girls, not health professionals |
|  | Betts, V. (2011) Transnational Gender, Sexuality and Identity Construction: Ambivalent Subjectivities of Somali Refugee Women in the GTA. MA Thesis, The University of Guelph, Canada. | Data related to healthcare experiences is from women/girls, not health professionals |
|  | Bravo Pastor del Mar, M. (2014) [Women Subjected to Female Genital Mutilation: Knowledge for the Nursing Discipline] La Voz de las Mujeres Sometidas a Mutilación Genital Femenina: Saberes Para la Disciplina Enfermera. University of Murcia, PhD Thesis, Spain. | Data related to healthcare experiences is from women/girls, not health professionals |
|  | Brown E, Carroll J, Fogarty C and Holt C. "They get a C-section . . . they gonna die": Somali women's fears of obstetrical interventions in the United States. *J Transcult Nurs*. 2010; 21: 220-7. | Data on FGM/C not presented |
|  | Brown E and Porter C. The tackling FGM initiative: evaluation of the second phase (2013-2016). Options UK, Report. 2016. | Not empirical research |
|  | Buckland RL. The everyday experience of Somali women in Canada: Implications for health. MSc Thesis, University of Ottawa (Canada), 1997 | Data presented on FGM/C, but no specific focus on FGM/C related healthcare |
|  | Burchill J. Safeguarding vulnerable families: work with refugees and asylum seekers. *Community Practitioner*. 2011; 84: 23-6. | Data presented on FGM/C, but no specific focus on FGM/C related healthcare |
|  | Carolan M. Antenatal care perceptions of pregnant African women attending maternity services in Melbourne, Australia. *Midwifery*. 2010; 26: 189-201. | Data on FGM/C not presented |
|  | Carroll, J., Epstein, R., Fiscella, K., Gipson, T., Volpe, E. & Jean-Pierre, P. (2007) Caring for Somali women: implications for clinician-patient communication. *Patient Educ. Couns.,* 66(3), 337-45. | Data related to healthcare experiences is from women/girls, not health professionals |
|  | Chalmers B and Hashi KO. 432 Somali women's birth experiences in Canada after earlier female genital mutilation. *Birth*. 2000; 27: 227-34. | Not qualitative research |
|  | Chalmers, B. & Omer-Hashi, K. (2002) What Somali women say about giving birth in Canada. *J. Reprod. Infant Psychol.,* 20(4), 267-282. | Data related to healthcare experiences is from women/girls, not health professionals |
|  | Christiansen CD. The lived experience of circumcision in immigrant Somali women: a Heideggerian hermeneutic analysis. MA Thesis, University of Illinois, 1995, | Data presented on FGM/C, but no specific focus on FGM/C related healthcare |
|  | Clarke ALF. The process of changed meanings: A study of the Canadian experience of Somali women in the Kitchener-Waterloo area in regard to female circumcision. PhD Thesis, Ann Arbor: Wilfrid Laurier University (Canada), 1995, | Not empirical research |
|  | Clayton-Hathway K. A Pilot Evaluation of Health Services for Communities Affected by FGM in Oxfordshire: Final Report. Oxford, UK: Healthwatch Oxfordshire, 2016. | Not qualitative research |
|  | Connor JJ, Hunt S, Finsaas M, Ciesinski A, Ahmed A and Robinson BBE. Sexual Health Care, Sexual Behaviors and Functioning, and Female Genital Cutting: Perspectives From Somali Women Living in the United States. *J Sex Res*. 2016; 53: 346-59. | Data presented on FGM/C, but no specific focus on FGM/C related healthcare |
|  | Dahlen UM. Female Genital Cutting: Phenomenological Interviews on the Ethiopian Immigrant Mothers' Experience. Masters Thesis, Regent University, 2011, | Data presented on FGM/C, but no specific focus on FGM/C related healthcare |
|  | Degni F, Suominen S, Essen B, El Ansari W and Vehvilainen-Julkunen K. Communication and cultural issues in providing reproductive health care to immigrant women: health care providers' experiences in meeting the needs of Somali women living in Finland. *Journal of Immigrant and Minority Health*. 2012; 14: 330-43. | Data on FGM/C not presented |
|  | Degni, F., Suominen, S.B., El Ansari, W., Vehviläinen-Julkunen, K. & Essen, B. (2014) Reproductive and maternity health care services in Finland: perceptions and experiences of Somali-born immigrant women. *Ethn. Health,* 19(3), 348-366. | Data related to healthcare experiences is from women/girls, not health professionals |
|  | d'Entremont, M., Smythe, L. & McAra-Couper, J. (2014) The sounds of silence: a hermeneutic interpretation of childbirth post excision. *Health Care Women Int.,* 35(3), 300-19. | Data related to healthcare experiences is from women/girls, not health professionals |
|  | Essén, B., Johnsdotter, S., Hovelius, B., Gudmundsson, S., Sjöberg, N.O., Friedman, J. & Östergren, P.O. (2000) Qualitative study of pregnancy and childbirth experiences in Somalian women resident in Sweden. *Br. J. Obstet. Gynaecol.,* 107(12), 1507-1512. | Data related to healthcare experiences is from women/girls, not health professionals |
|  | Essen B, Binder P and Johnsdotter S. An anthropological analysis of the perspectives of Somali women in the West and their obstetric care providers on caesarean birth. *Journal of Psychosomatic Obstetrics and Gynecology*. 2011; 32: 10-8. | Data on FGM/C not presented |
|  | Essen B, Johnsdotter S and Binder P. Not too far to walk but too far for reciprocity: Maternal mortality in a migration context using the 'three delays' framework. *International Journal of Gynecology and Obstetrics*. 2012; 119: S343. | Conference abstract |
|  | Feldman R. When maternity doesn't matter: Dispersing pregnant women seeking asylum. *British Journal of Midwifery*. 2014; 22: 23-8. | Data on FGM/C not presented |
|  | Finnstrom B and Soderhamn O. Conceptions of pain among Somali women. *J Adv Nurs*. 2006; 54: 418-25. | Data presented on FGM/C, but no specific focus on FGM/C related healthcare |
|  | Gali, M.A. (1997) Female Circumcision: A Transcultural Study of Attitudes, Identity and Reproductive Health of East African Immigrants. The Wright Institute, University of Berkeley, PhD Thesis, USA. | Data related to healthcare experiences is from women/girls, not health professionals |
|  | García Aguado S and Sánchez López MI. Knowledge of healthcare professionals about female genital mutilation. *Metas de Enfermería*. 2013; 16: 18-22. | Not qualitative research |
|  | Gele AA, Kumar B, Hjelde KH and Sundby J. Attitudes toward female circumcision among Somali immigrants in Oslo: a qualitative study. *International Journal of Women's Health*. 2012; 4: 7-17. | Data presented on FGM/C, but no specific focus on FGM/C related healthcare |
|  | Gele AA, Sagbakken M and Kumar B. Is female circumcision evolving or dissolving in Norway? A qualitative study on attitudes toward the practice among young Somalis in the Oslo area. *International Journal of Women's Health*. 2015; 7: 933-43. | Data presented on FGM/C, but no specific focus on FGM/C related healthcare |
|  | Gerrish K, Naisby A and Ismail M. Experiences of the diagnosis and management of tuberculosis: A focused ethnography of Somali patients and healthcare professionals in the UK. *J Adv Nurs*. 2013; 69: 2285-94. | Data on FGM/C not presented |
|  | Ghebre, R.G., Sewali, B., Osman, S., Adawe, A., Nguyen, H.T., Okuyemi, K.S. & Joseph, A. (2015) Cervical cancer: barriers to screening in the Somali community in Minnesota. *J Immigr Minor Health,* 17(3), 722-728. | Data related to healthcare experiences is from women/girls, not health professionals |
|  | Glavin K and Sæteren B. Cultural Diversity in Perinatal Care: Somali New Mothers' Experiences with Health Care in Norway. *Health Science Journal*. 2016; 10: 1-9. | Data on FGM/C not presented |
|  | Glazer, E. (2012) Embodiment, Pain and Circumcision in Somali-Canadian Women. University of Toronto, MSc Thesis, Canada. | Data related to healthcare experiences is from women/girls, not health professionals |
|  | Glazer E. Gendering chronic pain: Socio-cultural embodiment and circumcision in Somali women in Toronto. *Gend Med*. 2010; 7: 528. | Conference abstract |
|  | Glover, J., Liebling, H., Barrett, H. & Goodman, S. (2017) The psychological and social impact of female genital mutilation: a holistic conceptual framework. *J Int Stud,* 10(2), 219-238. | Data related to healthcare experiences is from women/girls, not health professionals |
|  | Goldblatt H. Caring for abused women: Impact on nurses' professional and personal life experiences. *J Adv Nurs*. 2009; 65: 1645-54. | Data on FGM/C not presented |
|  | Guerin, P.B., Allotey, P., Elmi, F.H. & Baho, S. (2006) Advocacy as a means to an end: assisting refugee women to take control of their reproductive health needs. *Women Health,* 43(4), 7-25. | Data related to healthcare experiences is from women/girls, not health professionals |
|  | Hai DNT and Ghebre R. Comparison of barriers to cervical cancer screening among Somali and Vietnamese women. *J Womens Health*. 2013; 22: 36. | Conference abstract |
|  | Henderson A. Factors influencing nurses' responses to abused women: What they say they do and why they say they do it. *Journal of Interpersonal Violence*. 2001; 16: 1284-306. | Data on FGM/C not presented |
|  | Herrel N, Olevitch L, DuBois DK, et al. Somali refugee women speak out about their needs for care during pregnancy and delivery. *Journal of Midwifery & Women's Health*. 2004; 49: 345-9. | Data on FGM/C not presented |
|  | Hill, N., Hunt, E. & Hyrkas, K. (2012) Somali immigrant women's health care experiences and beliefs regarding pregnancy and birth in the United States. *J. Transcult. Nurs.,* 23(1), 72-81. | Data related to healthcare experiences is from women/girls, not health professionals |
|  | Houston AR. (In)visible embodiment: Somali perspectives of diabetes and mental health in diaspora. MSc Thesis, Boston University, 2016, | Data presented on FGM/C, but no specific focus on FGM/C related healthcare |
|  | Hoyt MA. In her own words: An exploration of immigrant women's experiences of reproductive health care through ethnographic narrative. MA Thesis, University of New Brunswick (Canada), 2010, | Data on FGM/C not presented |
|  | Hussein, E. (2010) Women's Experiences, Perceptions and Attitudes of Female Genital Mutilation: The Bristol PEER Study. FORWARD, London. | Data related to healthcare experiences is from women/girls, not health professionals |
|  | Isman E, Ekeus C and Berggren V. Perceptions and experiences of female genital mutilation after immigration to Sweden: an explorative study. *Sexual and Reproductive Healthcare*. 2013; 4: 93-8. | Data presented on FGM/C, but no specific focus on FGM/C related healthcare |
|  | Jacoby SD, Lucarelli M, Musse F, Krishnamurthy A and Salyers V. A Mixed-Methods Study of Immigrant Somali Women's Health Literacy and Perinatal Experiences in Maine. *Journal of Midwifery and Women's Health*. 2015; 60: 593-603. | Data presented on FGM/C, but no specific focus on FGM/C related healthcare |
|  | Johansen REB. Experiencing sex in exile: can genitals change their gender? On conceptions and experiences related to female genital cutting (FGC) among Somalis in Norway. In: *Transcultural Bodies: Female Genital Cutting In Global Context Ed Ylva Hernlund & Bettina Shell-Duncan*. Rutgers University Press, 2007, p. 248-77. | Data presented on FGM/C, but no specific focus on FGM/C related healthcare |
|  | Johansen, R.E. (2017) Undoing female genital cutting: perceptions and experiences of infibulation, defibulation and virginity among Somali and Sudanese migrants in Norway. *Cult Health Sex,* 19(4), 528-542. | Data related to healthcare experiences is from women/girls, not health professionals |
|  | Johnsdotter S, Ingvarsdotter K, Ostman M and Carlbom A. Koran reading and negotiation with jinn: strategies to deal with mental ill health among Swedish Somalis. *Mental Health, Religion & Culture*. 2011; 14: 741-55. | Data on FGM/C not presented |
|  | Johnsdotter S and Essén B. It is only a tradition: making sense of Swedish Somalis' narratives of female circumcision and avoiding submission to hegemonic political discourse. *Crit Soc Policy*. 2005; 25: 577-89. | Not empirical research |
|  | Johnsdotter S and Essen B. Cultural change after migration: Circumcision of girls in Western migrant communities. *Best Practice and Research: Clinical Obstetrics and Gynaecology*. 2016; 32: 15-25. | Not empirical research |
|  | Johnsdotter S, Moussa K, Carlbom A, Aregai R and Essen B. "Never my daughters": A qualitative study regarding attitude change toward female genital cutting among Ethiopian and Eritrean families in Sweden. *Health Care Women Int*. 2009; 30: 114-33. | Data presented on FGM/C, but no specific focus on FGM/C related healthcare |
|  | Johnson-Agbakwu CE, Helm T, Killawi A and Padela AI. Perceptions of obstetrical interventions and female genital cutting: insights of men in a Somali refugee community. *Ethn Health*. 2014; 19: 440-5 | Focus on men, not women |
|  | Jones, A. (2010) Working Psychologically with Female Genital Mutilation: An Exploration of the Views and Experiences of Women who have Experienced FGM and of Clinical Psychologists. University of East London, Doctorate of Clinical Psychology, London. | Data related to healthcare experiences is from women/girls, not health professionals |
|  | Kallon I and Dundes L. The cultural context of the Sierra Leonean Mende woman as patient. *J Transcult Nurs*. 2010; 21: 228-36. | Not empirical research |
|  | Kaplan-Marcusan A, Del Rio NF, Moreno-Navarro J, et al. Female genital mutilation: perceptions of healthcare professionals and the perspective of the migrant families. *BMC Public Health*. 2010; 10: 193. | Not qualitative research |
|  | Kay M, Wijayanayaka S, Cook H and Hollingworth S. Understanding quality use of medicines in refugee communities in Australian primary care: A qualitative study. *Br J Gen Pract*. 2016; 66: e397-e409. | Data on FGM/C not presented |
|  | Khaja, K. (2004) Female Circumcision: Life Histories of Somali Women. The University of Utah, PhD Thesis, USA. | Data related to healthcare experiences is from women/girls, not health professionals |
|  | Khaja, K., Lay, K. & Boys, S. (2010) Female circumcision: toward an inclusive practice of care. *Health Care Women Int.,* 31(8), 686-99. | Data related to healthcare experiences is from women/girls, not health professionals |
|  | Koukoui S, Hassan G and Guzder J. The mothering experience of women with FGM/C raising 'uncut' daughters, in Ivory Coast and in Canada. *Reproductive Health*. 2017; 14: 1-11. | Data presented on FGM/C, but no specific focus on FGM/C related healthcare |
|  | Lazar J, Shipp M and Johnson C. Provider perceptions of sexual desire and dyspareunia among Somali women with female genital cutting. *J Sex Med*. 2010; 7: 148. | Conference abstract |
|  | Lane J and Cole G. Pregnancy, prenatal care and delivery perceptions and beliefs of resettled African refugee women in the western United States. *Contraception*. 2013; 88: 313. | Conference abstract |
|  | Leishman J. Perspectives of cultural competence in health care. *Nurs Stand*. 2004; 19: 33 | Data on FGM/C not presented |
|  | Lephard E and Haith-Cooper M. Pregnant and seeking asylum: Exploring women's experiences 'from booking to baby'. *British Journal of Midwifery*. 2016; 24: 130-6. | Data on FGM/C not presented |
|  | Lewig K, Arney F and Salveron M. Challenges to parenting in a new culture: Implications for child and family welfare. *Eval Program Plann*. 2010; 33: 324-32. | Data on FGM/C not presented |
|  | Leye E. Midwifery training needs identified when caring for women with female genital mutilation. *Evid Based Nurs*. 2016; 19: 7. | Not empirical research |
|  | Liao LM, Elliott C, Ahmed F and Creighton SM. Adult recall of childhood female genital cutting and perceptions of its effects: a pilot study for service improvement and research feasibility. *J Obstet Gynaecol*. 2013; 33: 292-5. | Not qualitative research |
|  | Lien IL and Schultz JH. Internalizing knowledge and changing attitudes to female genital cutting/mutilation. *Obstet Gynecol Int*. 2013; 2013: 467028. | Data presented on FGM/C, but no specific focus on FGM/C related healthcare |
|  | Lundberg, P.C. & Gerezgiher, A. (2008) Experiences from pregnancy and childbirth related to female genital mutilation among Eritrean immigrant women in Sweden. *Midwifery,* 24(2), 214-25. | Data related to healthcare experiences is from women/girls, not health professionals |
|  | Maier, C. (2003) [Echoes of Silence: Voices of Concern for Genital Mutilation in African Immigrant Women in Vienna: An Ethnological Study] Echo des Schweigens: Stimmen der Betroffenheit zur Genitalverstümmelung bei Afrikanischen Immigrantinnen in Wien: Ethnologische Studie. University of Vienna, PhD Thesis, Austria. | Data related to healthcare experiences is from women/girls, not health professionals |
|  | Maternity Action (2014) Women's Voices on Health: Addressing Barriers to Accessing Primary Care. Women’s Health and Equality Consortium, London. | Data related to healthcare experiences is from women/girls, not health professionals |
|  | McCarthy R and Haith-Cooper M. Evaluating the impact of befriending for pregnant asylum-seeking and refugee women. *British Journal of Midwifery*. 2013; 21: 404-9. | Data on FGM/C not presented |
|  | McLeish J. Maternity experiences of asylum seekers in England. *British Journal of Midwifery* 2005; 13: 782-5. | Data on FGM/C not presented |
|  | McNeely, S. & Christie-de Jong, F. (2016) Somali refugees' perspectives regarding FGM/C in the US. *Int J Migr Health Soc Care,* 12(3), 157-169. | Data related to healthcare experiences is from women/girls, not health professionals |
|  | Mitello L and Proietti A. Feminine genitals modification: survey of a new phenomenon for nurse today. Second Part. *Prof Inferm*. 2006; 59: 242-5. | Not qualitative research |
|  | Mitello L and Proietti A. Female genital modification: survey of new phenomenon for nurses today. First Part. *Prof Inferm*. 2006; 59: 171-81. | Not empirical research |
|  | Morris MD, Popper ST, Rodwell TC, Brodine SK and Brouwer KC. Healthcare barriers of refugees post-resettlement. *J Community Health*. 2009; 34: 529-38. | Data on FGM/C not presented |
|  | Morison L and others. How experiences and attitudes relating to female circumcision vary according to age on arrival in Britain: a study among young Somalis in London. *Ethnicity & Health*. 2004; 9: 75-100. | Data presented on FGM/C, but no specific focus on FGM/C related healthcare |
|  | Moxey, J.M. & Jones, L.L. (2016) A qualitative study exploring how Somali women exposed to female genital mutilation experience and perceive antenatal and intrapartum care in England. *BMJ Open,* 6(1), e009846. | Data related to healthcare experiences is from women/girls, not health professionals |
|  | Mwangi-Powell F. Female genital mutilation: a case study in Birmingham. FORWARD, Report, 2000. | Data presented on FGM/C, but no specific focus on FGM/C related healthcare |
|  | Murray, L., Windsor, C., Parker, E. & Tewfik, O. (2010) The experiences of African women giving birth in Brisbane, Australia. *Health Care Women Int.,* 31(5), 458-72. | Data related to healthcare experiences is from women/girls, not health professionals |
|  | Nash E and Ranka P. Female genital mutilation: Knowledge, confidence, and approach to care in clinical practice of midwives and nurses in the UK. *International Journal of Gynecology and Obstetrics*. 2015; 131: E110. | Not qualitative research |
|  | Norman, K., Gegzabher, S.B. & Otoo-Oyortey, N. (2016) "Between Two Cultures": A Rapid PEER Study Exploring Migrant Communities' Views on Female Genital Mutilation in Essex and Norfolk, UK. FORWARD & National FGM Centre Report, | Data related to healthcare experiences is from women/girls, not health professionals |
|  | Norman, K., Hemmings, J., Hussein, E. & Otoo-Oyortey, N. (2009) "FGM is Always With Us": Experiences, Perceptions and Beliefs of Women Affected by Female Genital Mutilation in London: Results from a PEER Study. Options Consultancy Services and FORWARD, London. | Data related to healthcare experiences is from women/girls, not health professionals |
|  | Nur Z. Cultural Competence in Health Care: Exploring the Experiences of Muslim Women within the Ontario Healthcare System. MSc Thesis, University of Ontario Institute of Technology (Canada), 2014, | Data on FGM/C not presented |
|  | O’Brien, O., Baldeh, F., Hassan, J. & Baillie, M. (2017) My Voice: Participatory Action Research Project with Men, Women and Young People on Female Genital Mutilation (FGM) in Scotland: (Phase 2). Queen Margaret University, Waverly Care, Edinburgh. | Data related to healthcare experiences is from women/girls, not health professionals |
|  | O’Brien, O., Baldeh, F., Sivapatham, S., Brown, E. & O’May, F. (2016) Participatory Action Research Project with Men, Women and Young People on Female Genital Mutilation (FGM) in Scotland: (Phase 1). Queen Margaret University, Waverly Care, Edinburgh. | Data related to healthcare experiences is from women/girls, not health professionals |
|  | Ogbagzy RA. The construction of Eritrean women's identity in the Canadian context. MA Thesis, University of Toronto (Canada), 1999, p. 236. | Data presented on FGM/C, but no specific focus on FGM/C related healthcare |
|  | Ogunsiji O. Understanding the dilemma of de-infibulation for women living with female genital mutilation. *Australian Nursing & Midwifery Journal*. 2015; 22: 51 | Not empirical research |
|  | Ogunsiji O and Wilkes L. Cultural explanations and procedures about female genital mutilation. *Australian Nursing & Midwifery Journal*. 2015; 22: 51 | Data presented on FGM/C, but no specific focus on FGM/C related healthcare |
|  | Ogunsiji O, Wilkes L, Peters K and Jackson D. Knowledge, attitudes and usage of cancer screening among West African migrant women. *J Clin Nurs*. 2013; 22: 1026-33. | Data on FGM/C not presented |
|  | Olsson E, Lau M, Lifvergren S and Chakhunashvili A. Community collaboration to increase foreign-born women's participation in a cervical cancer screening program in Sweden: A quality improvement project. *International Journal for Equity in Health*. 2014; 13 | Data on FGM/C not presented |
|  | Palfreyman, A., Brown, E. & Nam, S. (2011) Understanding Female Genital Mutilation in Birmingham: Findings from a PEER Study. Options Consultancy Services and Birmingham & Solihull Women’s Aid. | Data related to healthcare experiences is from women/girls, not health professionals |
|  | Pavlish, C.L., Noor, S. & Brandt, J., Somali immigrant women and the American health care system: discordant beliefs, divergent expectations, and silent worries, *Soc Sci Med.* 2010; 71:2, 353-361 | Data on FGM/C not presented |
|  | Peters K. Reasons why women choose a medical practice or a women’s health centre for routine health screening: worker and client perspectives. *J Clin Nurs*. 2010; 19: 2557-64. | Data on FGM/C not presented |
|  | Phillimore J. Migrant maternity in an era of superdiversity: new migrants' access to, and experience of, antenatal care in the West Midlands, UK. *Soc Sci Med*. 2016; 148: 152-9. | Data on FGM/C not presented |
|  | Quilliam S. Female genital mutilation: three questions for us to consider. *J Fam Plann Reprod Health Care*. 2015; 41: 235-7. | Not empirical research |
|  | Radford K. 'Unkind cuts': health policy and practice versus the health and emotional well-being of asylum-seekers and refugees in Ireland. *Journal of Ethnic and Migration Studies*. 2010; 36: 899-915. | Data on FGM/C not presented |
|  | Razzak M. Sexual medicine: Pain and pleasure-reconstruction after female genital mutilation. *Nature Reviews Urology*. 2012; 9. | Not empirical research |
|  | Recchia, N. & McGarry, J. (2017) "Don't judge me": narratives of living with FGM. *Int J Hum Rights Healthc,* 10(1), 4-13. | Data related to healthcare experiences is from women/girls, not health professionals |
|  | Reis E. Culture and cutting. *The Hastings Center Report*. 2012; 42: 3. | Not empirical research |
|  | Rimmer SN. [Theme: circumcision. Somali women talk about taboo]. *Sygeplejersken*. 1994; 94: 12-3. | Not empirical research |
|  | Roberts TL and Poblete X. Female genital mutilation: A survey of awareness, training and current practice. *Archives of Disease in Childhood: Annual Conference of the Royal College of Paediatrics and Child Health, RCPCH 2015*. Birmingham, UK: BMJ Publishing Group, 2015, p. G55. | Conference abstract |
|  | Rogers C and Earnest J. A Cross-Generational Study of Contraception and Reproductive Health Among Sudanese and Eritrean Women in Brisbane, Australia. *Health Care Women Int*. 2014; 35: 334-56. | Data on FGM/C not presented |
|  | Rosenberg LB, Gibson K and Shulman JF. When cultures collide: Female genital cutting and U.S. obstetric practice. *Obstet Gynecol*. 2009; 113: 931-4. | Not empirical research |
|  | Rosenberg E, Kirmayer LJ, Xenocostas S, Dao MD and Loignon C. GPs' strategies in intercultural clinical encounters. *Fam Pract*. 2007; 24: 145-51. | Data on FGM/C not presented |
|  | Roth K. Giving refuge: reflections on working with asylum seekers. *J Pain Symptom Manage*. 2010; 40: 149-54. | Not empirical research |
|  | Safari, F. (2013) A qualitative study of women's lived experience after deinfibulation in the UK. *Midwifery,* 29(2), 154-8. | Data related to healthcare experiences is from women/girls, not health professionals |
|  | Sala R and Manara D. Nurses and requests for female genital mutilation: cultural rights versus human rights. *Nurs Ethics*. 2001; 8: 247-58. | Not empirical research |
|  | Salad, J., Verdonk, P., de Boer, F. & Abma, T.A. (2015) "A Somali girl is Muslim and does not have premarital sex - is vaccination really necessary?" A qualitative study into the perceptions of Somali women in the Netherlands about the prevention of cervical cancer. *Int J Equity Health,* 14(1), 1-13. | Data related to healthcare experiences is from women/girls, not health professionals |
|  | Schuster S. 'Gosh': a cross-cultural encounter with a Somali woman, a male interpreter and a gynecologist on female genital cutting/mutilation. *Patient Educ Couns*. 2015; 98: 127-8. | Not empirical research |
|  | Shaw, E. (1985) Female circumcision: perceptions of clients and caregivers. *J. Am. Coll. Health,* 33(5), 193-7. | Data related to healthcare experiences is from women/girls, not health professionals |
|  | Shelp A. Women helping women: the Somali doula initiative. *International Journal of Childbirth Education*. 2004; 19: 4-7. | Not qualitative research |
|  | Shermarke, M.A.A. (1996) Understanding the Canadian Community Context of Female Circumcision. McGill University, MSc Thesis, Canada. | Data related to healthcare experiences is from women/girls, not health professionals |
|  | Shipp MPL, Francis SA, Fluegge KR and Asfaw SA. Perceived Health Issues: A perspective from East-African immigrants. *Health, Culture and Society*. 2014; 6: 13-32. | Data on FGM/C not presented |
|  | Sigurjonsson H, Jordal M and Lundgren TK. Reconstructive surgery after female genital mutilation. *International Journal of Gynecology and Obstetrics*. 2015; 131: E318-E9. | Conference abstract |
|  | Simpson J, Robinson K, Creighton SM and Hodes D. Female genital mutilation: the role of health professionals in prevention, assessment, and management. *Br Med J*. 2012; 344. | Not empirical research |
|  | Sokolik L. Somali immigrant and refugee women's experience of pregnancy and childbirth in the American healthcare system. *Commun Nurs Res*. 2007; 40: 538 | Conference abstract |
|  | Straus, L., McEwen, A. & Hussein, F.M. (2009) Somali women's experience of childbirth in the UK: perspectives from Somali health workers. *Midwifery,* 25(2), 181-6. | Data related to healthcare experiences is from women/girls, not health professionals |
|  | Sudbury H and Robinson A. Barriers to sexual and reproductive health care for refugee and asylum-seeking women. *British Journal of Midwifery*. 2016; 24: 275-81. | Not empirical research |
|  | Sundby J. Genital mutilation of women--is it a concern for Gynecologists? *Acta Obstet Gynecol Scand*. 1996; 75: 513-5. | Not empirical research |
|  | Talle A. From "complete" to "impaired" body: Female circumcision in Somalia and London. *Disability in Local and Global worlds*. Berkeley, CA: University of California Press; US, 2007, p. 56-77. | Not empirical research |
|  | Taillens F. ["I will not circumcise my daughter"]. *Krankenpflege - Soins Infirmiers*. 2012; 105: 55. | Not empirical research |
|  | Tait P. Refugee Women in Australia and Woman-Centered Midwifery Care. *Nuritinga*. 2013: 31-9. | Not empirical research |
|  | Tantet C, Aupiais C, Sorge F, Levy D, Lafon-Desmurs B and Faye A. MIG-01 - Female genital mutilations: Evaluation of the knowledge of general practitioners and physicians in travel counseling. *Med Mal Infect*. 2016; 46: 77. | Not qualitative research |
|  | Tatah EF. Female Circumcision: A Phenomenological Study of Somalian Immigrants to the United States. PhD Thesis, Walden University, 2016, | Data presented on FGM/C, but no specific focus on FGM/C related healthcare |
|  | Thierfelder, C., Tanner, M. & Bodiang, C.M.K. (2005) Female genital mutilation in the context of migration: experience of African women with the Swiss health care system. *Eur. J. Public Health,* 15(1), 86-90. | Data related to healthcare experiences is from women/girls, not health professionals |
|  | Trueland J. School nurses take lead on FGM. *Nurs Stand*. 2014; 28: 22-3. | Not empirical research |
|  | Upvall, M.J., Mohammed, K. & Dodge, P.D. (2009) Perspectives of Somali Bantu refugee women living with circumcision in the United States: a focus group approach. *Int. J. Nurs. Stud.,* 46(3), 360-8. | Data related to healthcare experiences is from women/girls, not health professionals |
|  | Vercoutere A, Temmerman M and Leye E. Female genital mutilation, a story far away from home? Ethical, legal and clinical aspects of mutilated women in western countries. *Tijdschrift voor Geneeskunde*. 2011; 67: 237-41. | Not empirical research |
|  | Villani M. From the “maturity” of a woman to surgery: Conditions for clitoris repair. *Sexologies*. 2009; 18: 259-61. | Not qualitative research |
|  | Villani M. The way of speaking about "mutilation" and performing "repairing". *J Sex Med*. 2011; 8: 242. | Not qualitative research |
|  | Villani M. Experiences with clitoris repair with counselling or counselling only in France, for effect on sexual satisfaction for women with FGM. *Int J Gynecol Obstet*. 2012; 119: S255. | Conference abstract |
|  | Villani M, Griffin J and Bodenmann P. In their own words: the health and sexuality of immigrant women with infibulation living in Switzerland. *Soc Sci*. 2016; 5: 1. | Data presented on FGM/C, but no specific focus on FGM/C related healthcare |
|  | Vissandjee B, Denetto S, Migliardi P and Proctor J. Female genital cutting (FGC) and the ethics of care: community engagement and cultural sensitivity at the interface of migration experiences. *BMC Int Health Hum Rights*. 2014; 14: 1-18. | Not empirical research |
|  | Vloeberghs E, Knipscheer J, van der Kwaak A, Naleie Z and van den Muijsenbergh M. Veiled Pain: A Study In The Netherlands On The Psychological, Social And Relational Consequences Of Female Genital Mutilation. Pharos–Dutch National Knowledge and Advisory Centre on Refugees and Migrants' Health, 2010. | Duplicate report of included paper |
|  | Vloeberghs, E., van der Kwaak, A., Knipscheer, J. & van den Muijsenbergh, M. (2012) Coping and chronic psychosocial consequences of female genital mutilation in the Netherlands. *Ethn. Health,* 17(6), 677-95. | Data related to healthcare experiences is from women/girls, not health professionals |
|  | Wade L. The politics of acculturation: Female genital cutting and the challenge of building multicultural democracies. *Soc Probl*. 2011; 58: 518-37. | Data presented on FGM/C, but no specific focus on FGM/C related healthcare |
|  | Williams KW. Somali Bantu health experience: Refugee resettlement in South Carolina. PhD Thesis, University of South Carolina, 2006, p. 185. | Data on FGM/C not presented |
|  | Williams N, Offer GJ, Williams S and Everson N. Parental attitude in children referred for circumcision. *Br J Surg*. 1997; 84: 1722-3. | Data on FGM/C not presented |
|  | Willis S. The cutting season. *Journal of Family Health Care*. 2014; 24: 9-12. | Not empirical research |
|  | Wiklund, H., Aden, A.S., Högberg, U., Wikman, M. & Dahlgren, L. (2000) Somalis giving birth in Sweden: a challenge to culture and gender specific values and behaviours. *Midwifery,* 16(2), 105-115. | Data related to healthcare experiences is from women/girls, not health professionals |
|  | Wimmer-Puchinger B, Wolf H and Engleder A. [Female migrants in the health care system. Health care utilisation, access barriers and health promotion strategies]. *Migrantinnen im Gesundheitssystem Inanspruchnahme, Zugangsbarrieren und Strategien zur Gesundheitsforderung*. 2006; 49: 884-92. | Not empirical research |
|  | Young H and McGrath K. A review of circumcision in New Zealand: 'I never liked doing them and I was pleased to give them up'. *Understanding Circumcision: A Multi-Disciplinary Approach To A Multi-Dimensional Problem*. Kluwer Academic Publishers, 2001. | Data on FGM/C not presented |
|  | Yusuf L and Negash S. Vaginal calculus following severe form of female genital mutilation: a case report. *Ethiop Med J*. 2008; 46: 185-8. | Not OECD |
|  | Zenner N, Liao LM, Richens Y and Creighton SM. Quality of obstetric and midwifery care for pregnant women who have undergone female genital mutilation. *J Obstet Gynaecol*. 2013; 33: 459-62. | Not qualitative research |
