# Supplementary material for: Crossing cultural divides: A qualitative systematic review of factors influencing the provision of healthcare related to female genital mutilation from the perspective of health professionals
Source: PLoS One. 2019 Mar 4;14(3):e0211829. doi: 10.1371/journal.pone.0211829 (PMC6398829; doi:10.1371/journal.pone.0211829)
Supplement: S4 Table — (DOCX) [file pone.0211829.s004.docx]

**S5 Table of Operational Criteria of Study Relevance**

| **Study Relevance** | **Definition** |
| --- | --- |
| High (specific) | FGM/C-specific healthcare (e.g. the study is focused on a specific aspect of care related directly to FGM/C, e.g. deinfibulation, childbirth for women who have had FGM/C, psychological care) |
| Medium (direct) | Other healthcare context (e.g. where the study focus is on the maternity care experience of a particular group more generally and where some of the findings relate to the experience of FGM/C) |
| Low (indirect) | Where the study focus is on general attitudes towards FGM/C and/or experiences and consequences of FGM - & where some FGM/C-related healthcare issues are reported, but are not the main focus of the paper |
