# Supplementary material for: Crossing cultural divides: A qualitative systematic review of factors influencing the provision of healthcare related to female genital mutilation from the perspective of health professionals
Source: PLoS One. 2019 Mar 4;14(3):e0211829. doi: 10.1371/journal.pone.0211829 (PMC6398829; doi:10.1371/journal.pone.0211829)
Supplement: S5 Table — (DOCX) [file pone.0211829.s005.docx]

**S6 Theme Matrix (Mapping Studies to Themes)**

|  | | **1.**  **Knowledge and training** | | **2. Communication is key** | | | **3.**  **Encountering the ‘other’ in clinical practice: negotiating cultural dissonance and achieving cultural understanding** | | | | | **4.**  **Identifying FGM/C** | | **5.**  **Clinical management practices: inconsistent and variable** | | | | | **6.**  **Optimal service development** | | |
| --- | --- | --- | --- | --- | --- | --- | --- | --- | --- | --- | --- | --- | --- | --- | --- | --- | --- | --- | --- | --- | --- |
| **No.** | **Study Reference** | **1.1** | **1.2** | **2.1** | **2.2** | **2.3** | **3.1** | **3.2** | **3.3** | **3.4** | **3.5** | **4.1** | **4.2** | **5.1** | **5.2** | **5.3** | **5.4** | **5.5** | **6.1** | **6.2** | **6.3** |
| **1** | Abdi, R. (2012) [1] |  |  |  |  |  | X |  |  |  |  | X |  |  |  |  |  |  |  |  |  |
| **2** | Behrendt, A. (2011) [2] | X | X | X |  |  |  |  |  |  | X | X |  |  |  |  |  | X | X | X |  |
| **3** | Bergqvist, H., & Svensson, J. (2016) [3] | X | X | X | X |  | X |  |  |  | X | X | X |  | X |  | X | X |  |  |  |
| **4** | Bibi, N., & Rahimian, N. (2013) [4] | X | X | X | X | X | X | X | X |  | X | X | X |  | X |  |  | X | X | X | X |
| **5** | Brodin, E., & Mårtensson, N. (2016) [5] | X | X | X | X |  |  |  | X | X | X | X | X |  | X |  | X | X | X |  |  |
| **6** | Bulman, K. H., & McCourt, C. (2002) [6] | X |  | X | X |  |  | X |  |  | X |  | X |  | X |  | X | X |  |  |  |
| **7** | Bulman, K., & McCourt, C. (1997) [7] |  | X |  |  |  |  |  | X |  |  |  |  |  |  |  |  |  |  |  |  |
| **8** | Burchill, J., & Pevalin, D. J. (2014) [8] |  | X | X | X |  |  |  |  |  | X |  |  |  |  |  |  |  |  |  |  |
| **9** | Byrskog, U., Olsson, P., Essen, B., & Allvin, M. K. (2015) [9] |  |  | X | X | X |  | X |  |  | X |  | X |  |  |  |  |  |  |  | X |
| **10** | Dawson, A. J., Turkmani, S., Varol, N., Nanayakkara, S., Sullivan, E., & Homer, C. S. (2015) [10] | X | X | X | X | X | X | X | X |  | X | X | X | X | X | X | X | X | X | X | X |
| **11** | Fawcett, L. (2014) [11] | X |  | X | X | X | X | X | X | X | X |  | X |  | X |  |  | X |  |  |  |
| **12** | Gertsson, M., & Serpan, H. (2009) [12] | X | X |  | X |  | X | X |  |  | X | X |  |  | X | X |  |  | X | X |  |
| **13** | Holm, L., & Kammensjö, H. (2012) [13] | X | X | X | X | X | X | X |  |  | X |  |  |  | X |  | X |  | X | X |  |
| **14** | Hussen, M. A. (2014) [14] | X | X |  | X | X |  |  |  |  | X | X | X |  | X |  |  |  |  | X | X |
| **15** | Jatau, M. (2011) [15] | X | X | X | X |  |  | X |  | X | X |  | X |  | X |  |  |  |  |  |  |
| **16** | Johansen, R. E. (2006) [16] | X |  |  | X |  | X | X | X |  |  |  | X |  | X | X |  | X |  |  |  |
| **17** | Johansen, R. E. (2017) [17] | X |  |  |  |  |  | X |  |  |  |  |  |  |  |  |  |  | X | X |  |
| **18** | Lazar, J. N., Johnson-Agbakwu, C. E., Davis, O. I., & Shipp, M. P. L. (2013) [18] | X | X | X | X | X | X | X | X |  | X |  | X |  | X |  | X |  |  |  |  |
| **19** | León-Larios, F., & Casado-Mejía, R. (2012) [19] | X | X | X | X |  |  | X | X | X |  | X | X |  | X |  | X |  |  |  |  |
| **20** | Leval, A., Widmark, C., Tishelman, C., & Maina Ahlberg, B. (2004) [20] | X |  |  | X |  | X | X | X |  |  |  |  |  |  | X |  |  | X |  |  |
| **21** | Moore, K. (2012) [21] | X | X | X | X |  | X |  | X |  | X |  | X | X | X | X | X |  | X | X | X |
| **22** | Ogunsiji, O. (2015) [22] | X | X |  |  |  | X |  |  |  | X |  |  |  |  | X |  |  |  |  |  |
| **23** | Ogunsiji, O. (2016) [23] | X |  |  | X | X |  |  | X | X | X | X | X | X | X | X | X | X |  |  |  |
| **24** | Rubin, E. A. (2000) [24] |  |  | X | X | X | X | X | X |  | X | X |  |  |  |  |  |  |  |  |  |
| **25** | Thierfelder, C. (2003) [25] | X | X | X | X | X | X | X | X | X | X | X | X | X | X | X | X | X | X | X | X |
| **26** | Vangen, S., Johansen, R. E. B., Sundby, J., Traeen, B., & Stray-Pedersen, B. (2004) [26] | X | X |  | X | X |  | X |  |  |  |  | X | X | X | X | X | X | X |  |  |
| **27** | Vaughan, C., White, N., Keogh, L., Tobin, J., Ha, B., Ibrahim, M., & Bayly, C. (2014) [27] | X | X | X | X | X | X | X |  |  | X | X | X |  | X |  | X | X | X | X | X |
| **28** | Vaughan, C., White, N., Keogh, L., Tobin, J., Murdolo, A., Quiazon, R., & Bayly, C. (2014) [28] | X | X | X | X | X |  | X |  | X | X | X | X | X | X |  | X |  |  | X | X |
| **29** | Widmark, C., Leval, A., Tishelman, C., & Ahlberg, B. M. (2010) [29] | X | X | X |  |  | X | X | X |  | X |  | X | X | X | X | X |  |  |  |  |
| **30** | Widmark, C., Tishelman, C., & Ahlberg, B. M. (2002) [30] | X | X | X | X |  | X | X | X | X | X | X | X | X | X | X | X |  | X |  | X |
|  | **Total Studies per Theme** | **25** | **21** | **20** | **24** | **13** | **17** | **20** | **15** | **8** | **23** | **15** | **20** | **8** | **21** | **11** | **15** | **12** | **13** | **11** | **9** |

**Theme Key – for Theme Matrix**

| **Descriptive Theme**  **No.** | **Theme Name** |
| --- | --- |
| **Analytical Theme 1: Knowledge and training** | |
| **1.1** | Knowledge and awareness |
| **1.2** | Education and training |
| **Analytical Theme 2: Communication is key** | |
| **2.1** | Language barriers and interpretation challenges |
| **2.2** | Talking about a sensitive topic |
| **2.3** | Women also find FGM/C hard to talk about |
| **Analytical Theme 3: Encountering the ‘other’ in clinical practice: negotiating cultural dissonance and achieving cultural understanding** | |
| **3.1** | Attitudes towards FGM/C: mixed emotions |
| **3.2** | Cultural dissonance – control and resistance in clinical encounters |
| **3.3** | Acknowledging the role of the family |
| **3.4** | Gender of the provider |
| **3.5** | Crossing the cultural divide – strategies and elements of culturally sensitive care |
| **Analytical Theme 4: Identifying FGM/C: hit and miss** | |
| **4.1** | Presentation and help seeking |
| **4.2** | Practices and processes around identifying FGM/C |
| **Analytical Theme 5: Clinical management practices: inconsistent and variable** | |
| 5.1 | Deinfibulation timing |
| 5.2 | Deinfibulation practice |
| 5.3 | Reinfibulation ambivalence |
| 5.4 | Need for guidelines |
| 5.5 | Psychological issues |
| **Analytical Theme 6: Optimal service development for FGM/C care** | |
| 6.1 | Provider’s role in prevention |
| 6.2 | Community engagement and education |
| 6.3 | Specialist services |

**References for Included Studies in Theme Matrix**

1. Abdi R. Carving culture: Creating identity through female genital cutting. Durham Anthropology Journal. 2012;18(1):115-53.

2. Behrendt A. Listening to African Voices: Female Genital Mutilation/Cutting among Immigrants in Hamburg: Knowledge, Attitudes and Practice. Hamburg, Germany: Plan; 2011.

3. Bergqvist H, Svensson J. [Midwives Experiences of Encounters wth Young Women who come from Areas Where the Practice of Genital Mutilation is Common] Barnmorskors Erfarenheter Av Möten Med Unga Kvinnor Som Kommer Från Områden Där Kvinnlig Könsstympning är Aanligt Förekommande. MSc Thesis, Sweden: University of Skövde; 2016.

4. Bibi N, Rahimian N. [Nurses’ Experience and Knowledge about Female Genital Mutilation] Sjuksköterskans Erfarenheter Och Kunskaper Om Kvinnlig Könssympning. MSc Thesis, Sweden: Sophiahemmet University; 2013.

5. Brodin E, Mårtensson N. [District Nurses' Knowledge and Experience of Female Genital Mutilation] Distriktssköterskors Kunskap Och Erfarenhet Av Kvinnlig Könsstympning. MSc Thesis, Sweden: University of Örebro; 2016.

6. Bulman KH, McCourt C. Somali refugee women's experiences of maternity care in west London: a case study. Crit Public Health. 2002;12(4):365-80.

7. Bulman K, McCourt C. Report on Somali Womens' Experiences of Maternity Services. London, UK: Centre for Midwifery Practice, Wolfson Institute of Health Sciences, Thames Valley University and Hammersmith Hospitals National Health Service Trust; 1997.

8. Burchill J, Pevalin DJ. Demonstrating cultural competence within health-visiting practice: working with refugee and asylum-seeking families. Divers Equal Health Care. 2014;11(2):151-9.

9. Byrskog U, Olsson P, Essen B, Allvin MK. Being a bridge: Swedish antenatal care midwives' encounters with Somali-born women and questions of violence - a qualitative study. BMC Pregnancy Childbirth. 2015;15(1).

10. Dawson AJ, Turkmani S, Varol N, Nanayakkara S, Sullivan E, Homer CS. Midwives' experiences of caring for women with female genital mutilation: insights and ways forward for practice in Australia. Women Birth. 2015;28(3):207-14.

11. Fawcett L. Somali Refugee Women and their U.S. Healthcare Providers: Knowledge, Perceptions and Experiences of Childbearing [Doctor of Philosophy]. PhD Thesis, USA: Arizona State University; 2014.

12. Gertsson M, Serpan H. [Meeting with the Unthinkable: Value Conflicts in Meetings with Women Vulnerable to Female Genital Mutilation] Mötet Med Det Otänkbara: Värdekonflikter I Mötet Med Kvinnor Utsatta för Kvinnlig Könsstympning. MSc Thesis, Sweden: University of Kalmar; 2009.

13. Holm L, Kammensjö H. [School Nurses' Experiences of Female Genital Mutilation among Girls] Skolsköterskors Upplevelser Kring Kvinnlig Könsstympning Bland Flickor. MSc Thesis, Sweden: University of Skövde; 2012.

14. Hussen MA. Services for Women with Female Genital Mutilation in Christchurch: Perspectives of Women and their Health Providers. MSc Thesis, New Zealand: University of Canterbury; 2014.

15. Jatau M. Living Between Two Cultures: A Reproductive Health Journey of African Refugee Women [Doctor of Philosophy]. PhD Thesis, USA: Arizona State University; 2011.

16. Johansen RE. Care for infibulated women giving birth in Norway: an anthropological analysis of health workers' management of a medically and culturally unfamiliar issue. Med Anthropol Q. 2006;20(4):516-44.

17. Johansen RE. Virility, pleasure and female genital mutilation/cutting: a qualitative study of perceptions and experiences of medicalized defibulation among Somali and Sudanese migrants in Norway. Reprod Health. 2017;14(1):25.

18. Lazar JN, Johnson-Agbakwu CE, Davis OI, Shipp MPL. Providers' perceptions of challenges in obstetrical care for Somali women. Obstet Gynecol Int. 2013;2013:149640.

19. León-Larios F, Casado-Mejía R. [Influence of gender on knowledge, perception and approach to harmful traditional practices: female genital mutilation]. Evidentia. 2012;9(40):1-7.

20. Leval A, Widmark C, Tishelman C, Maina Ahlberg B. The encounters that rupture the myth: contradictions in midwives' descriptions and explanations of circumcised women immigrants' sexuality. Health Care Women Int. 2004;25(8):743-60.

21. Moore K. Female Genital Mutilation and Cultural Competency: Moving Towards Improved Management of Obstetric Care [Masters Dissertation]. MSc Thesis, Edinburgh: Queen Margaret University; 2012.

22. Ogunsiji O. Female genital mutilation (FGM): Australian midwives’ knowledge and attitudes. Health Care Women Int. 2015;36(11):1179-93.

23. Ogunsiji O. Australian midwives' perspectives on managing obstetric care of women living with female genital circumcision/mutilation. Health Care Women Int. 2016;37(10):1156-69.

24. Rubin EA. When Cultures Collide: An Exploration of Cultural Competence and Cross-Cultural Communication between American Medical Providers and Immigrant Women who have been Circumcised. PhD Thesis, USA: University of Massachusetts; 2000.

25. Thierfelder C. Female Genital Mutilation and the Swiss Health Care System. PhD Thesis, Switzerland: University of Basel; 2003.

26. Vangen S, Johansen REB, Sundby J, Traeen B, Stray-Pedersen B. Qualitative study of perinatal care experiences among Somali women and local health care professionals in Norway. Eur J Obstet Gynecol Reprod Biol. 2004;112(1):29-35.

27. Vaughan C, White N, Keogh L, Tobin J, Ha B, Ibrahim M, et al. Listening to North Yarra Communities about Female Genital Cutting. Melbourne, Australia: The University of Melbourne; 2014. Contract No.: ISBN 978 0 9925013 0 3.

28. Vaughan C, White N, Keogh L, Tobin J, Murdolo A, Quiazon R, et al. Female Genital Mutilation/Cutting in Regional Victoria. Research to Practice. Melbourne, Australia: The University of Melbourne; 2014. Report No.: 1470-0328 Contract No.: ISBN 978 0 9925013 1 0.

29. Widmark C, Leval A, Tishelman C, Ahlberg BM. Obstetric care at the intersection of science and culture: Swedish doctors' perspectives on obstetric care of women who have undergone female genital cutting. J Obstet Gynaecol. 2010;30(6):553-8.

30. Widmark C, Tishelman C, Ahlberg BM. A study of Swedish midwives' encounters with infibulated African women in Sweden. Midwifery. 2002;18(2):113-25.
