# Supplementary material for: Crossing cultural divides: A qualitative systematic review of factors influencing the provision of healthcare related to female genital mutilation from the perspective of health professionals
Source: PLoS One. 2019 Mar 4;14(3):e0211829. doi: 10.1371/journal.pone.0211829 (PMC6398829; doi:10.1371/journal.pone.0211829)
Supplement: S6 Table — (DOCX) [file pone.0211829.s006.docx]

**S7 Detailed Characteristics of Included Studies**

| No. | Study | Country | Purpose or Aims | Region and Setting | Methodology, theory and framework | Methods and recruitment | Data Analysis | Whole sample | Relevant number |
| --- | --- | --- | --- | --- | --- | --- | --- | --- | --- |
|  | Abdi, R. (2012) [1] | UK | To establish the way in which Somali women living in London begin to perceive themselves and their identity as they carry out their everyday lives. How do they react to the questioning and probing gaze of others, for example, within a health setting? | East and Northeast regions of London.  Clinic setting | Narrative and life-history approach | Interviews were mainly ﬂowing narratives. Recruited by snowballing sampling method. | Analysis using theoretical concept of the ‘three-bodies’. | 16 Somali women and 2 men  2 gynaecologists, a counsellor and 1 midwife. | 2 gynaecologists, a counsellor & 1 midwife. |
|  | Behrendt, A. (2011) [2] | Germany | To gather ﬁrst-hand information on how the immigrant populations feel about and deal with the practice of FGM/C in the context of migration. Where and under what circumstances do women who have undergone FGM/C seek medical care? What precautions do they take during pregnancy? Do they participate in preventive care programs? | Hamburg | Participatory, cooperative approach which engages community members in a joint evaluation research process. A triangulated research design using qualitative and quantitative data from different target groups. | Mixed methods.  Qualitative and quantitative data collection from different target groups (1) key informant (health professional) interviews, (2) semi-structured individual interviews with African community members, (3) survey with community members.  Contacted gynaecologists working in neighbourhoods with a high proportion of immigrants and health personnel of African origin. | Qualitative data -  analysed and classiﬁed into themes.  Quantitative data  analysed using SPSS | Total number of 1,767 participants with African migration background were interviewed for the quantitative survey  91 semi structured interviews with African community members  13 interviews with health professionals  Age range 15-83 years | 13 health professionals: 6 female gynaecologists, other health personnel (midwives, nurses, dermatologist, paediatrician) |
|  | Bergqvist, H., & Svensson, J. (2016) [3] | Sweden | To highlight midwives’ experiences at youth clinics when they met with women who came from areas where FGM/C was common | Southwestern Sweden.  At the midwives’ workplace in a separate room. One telephone interview. | Qualitative methods and an inductive approach | Semi-structured interviews.  The head of  department/ area manager was asked to select respondents based on the criteria and invite them to participate in the study. | Content analysis | 8 midwives  Age range 32-59 years | 8 midwives |
|  | Bibi, N., & Rahimian, N. (2013) [4] | Sweden | To investigate nurses’ knowledge and experience of FGM | Stockholm –  In a private room at the department or clinic where the nurse worked and in a café. | Qualitative interview study | Semi-structured interviews.  Participants recruited from selected centres. | Thematic analysis | 11 nurses | 11 nurses  (6 nurses employed at gynaecological departments, 1 school nurse, 1 health centre nurse and 3 nurses working in a geriatric department) |
|  | Brodin, E., & Mårtensson, N. (2016) [5] | Sweden | To describe the knowledge and experiences of district nurses in caring for women with FGM/C | A region of central Sweden.  All 9 interviews took place in a private room at the participant’s workplace. | A qualitative interview study - descriptive design with an inductive approach. | Unstructured interviews | Content analysis | 9 participants  Age range 33-64 years, with a median age of 51. | 9 female district nurses |
|  | Bulman, K. H., & McCourt, C. (2002) [6] | UK | To develop an understanding of the reality faced by Somali women in their contacts with the maternity services in the UK and to explore professionals perceptions of Somali women and their maternity care needs - how they approached providing for these needs and their ideas for improving services. | West London  Conducted in participants homes or clinics | Qualitative (ethnographic context narrative approach) | 2 focus groups with midwives, (caseload and hospital midwives).  Individual interviews with a Somali health-link worker, a woman on the Health and Race Working Group, and an obstetric registrar.  6 individual interviews and two focus groups with Somali women, recruited via snowball sampling method | Thematic analysis | Health workers interviewed (exact number not specified)  12 Somali women interviewed. | 2 focus groups with midwives (number not stated)  Individual interviews with 3 others (a Somali health-link worker, a woman on the Health and Race Working Group, and an obstetric registrar) |
|  | Bulman, K., & McCourt, C. (1997) [7] | UK | Conducted as part of the evaluation of One-to-One Midwifery which was implemented by Queen Charlotte and Chelsea and Hammersmith maternity services in 1993.  To develop an understanding of the reality faced by Somali women in their contacts with the maternity services in the UK and to explore professionals perceptions of Somali women and their maternity care needs - how they approached providing for these needs and their ideas for improving services. | West London | Qualitative study | Interview approach was open and semi-structured. | Not stated | Not stated | 2 focus groups with midwives (number not stated)  Individual interviews with 3 others (a Somali health-link worker, a woman on the Health and Race Working Group, and an obstetric registrar) |
|  | Burchill, J., & Pevalin, D. J. (2014) [8] | UK | Explored the experiences of health visitors working with refugee and asylum seeking families in central London, and assessed the dimensions of their cultural competency. | A borough of London, at the various health centres in which participants worked. | Qualitative study using Quickfall’s ﬁve-step model | In-depth interviews. The health visitors who worked in the borough were approached at their main professional meeting, where a presentation provided details of the study with a request for participation. | An interpretive, thematic based analytical method using the Framework approach. | 14 health visitors | 14 health visitors |
|  | Byrskog, U., Olsson, P., Essen, B., & Allvin, M. K. (2015) [9] | Sweden | To explore ways antenatal care midwives in Sweden work with Somali born women and the questions of exposure to violence. | Middle and North of Sweden, setting not stated. | A qualitative approach | Semi-structured interviews.  A purposive sample of midwives was recruited from 11 antenatal clinics; one to two midwives from each clinic. | Thematic analysis | 17 midwives | 17 midwives |
|  | Dawson, A. J., Turkmani, S., Varol, N., Nanayakkara, S., Sullivan, E., & Homer, C. S. (2015) [10] | Australia | To provide an insight into midwives views and experiences of working with women affected by FGM. | Urban New South Wales - in meeting rooms in the hospitals in which midwives worked during time allocated for professional educational development. | A descriptive-interpretive qualitative research method. | Focus group discussions with midwives.  Clinical Nurse Midwifery Unit Manager distributed the invitation to registered midwives working in the hospital with a range of skills and experience. Four antenatal clinics and birthing units in 3 hospitals were purposively selected. | Thematic analysis | 48 midwives | 48 midwives |
|  | Fawcett, L. (2014) [11] | USA | To identify and measure patterns of cultural knowledge and variations (cross-cultural agreement and disagreement) between Somali resettled refugee women (SRRW) and healthcare providers (HCP) on childbearing models. To investigate and measure intra-cultural variations in the domain of childbearing knowledge among Somali resettled refugee women. To explore the subjective and intersubjective perceptions of female circumcision. | Arizona,  Six of the interviews were conducted in the clinical settings, two in a café, and two in private homes. | Quantitative survey and ethnographic methodologies and phenomenological approaches using open-ended semi-structured interviews.  Used the theory of ‘Cultural Consensus Model’ and concepts of embodiment. | Methods included culture consensus questionnaires, semi-structured interviews and participatory observations.  A group of Healthcare Providers (HCPs) were purposively selected for the in-depth semi-structured interviews. | Phenomenological analysis and inductive explorative approach | Questionnaires (N=174)  Interviews with  Somali resettled refugee women (SRRW, n=30) and healthcare providers (HCP, n=10)  All health professionals participants were female, with an age range of 18-70 years | 10 health professionals  (2 medical doctors; 1 midwife, 2 nurse practitioners, and 5 labour and delivery nurses) |
|  | Gertsson, M., & Serpan, H. (2009) [12] | Sweden | To explore how professionals meet with women who have been subjected to female genital mutilation – in order to gain a greater understanding of how professionals handle cultural meetings and the conflict of values that may arise when encountering vulnerable women. | A medium sized Swedish city in an area with a high population of immigrants.  Conducted at the education centre. | Qualitative method, using case study. | Semi-structured qualitative interviews with 4 people using a prepared questionnaire.  Purposive sampling from centres who come into contact with women who have experienced female genital mutilation. | Comprehensive analysis | 4 professional participants: 1 midwife, one social worker, 1 teacher, and 1 community support officer. | 1 midwife |
|  | Holm, L., & Kammensjö, H. (2012) [13] | Sweden | To highlight school nurses’ experience of female genital mutilation in schools. | Municipality in southern Sweden.  Setting was school nurses’ workplaces. | A qualitative approach. | Semi-structured interviews. School nurses were invited by email | Qualitative content analysis | 11 school nurses | 11 school nurses |
|  | Hussen, M. A. (2014) [14] | New Zealand | To understand the situation of both women with FGM and their health providers, in order to increase knowledge of FGM and ensure improved health services for women | Christchurch,  At health providers work place. | A narrative approach using qualitative descriptive research methods | Individual semi-structured interviews with service providers.  Focus groups with women from east Africa | Thematic analysis | 3 health providers  20 women in focus groups | 3 health providers (GP, nurse & health social worker). |
|  | Jatau, M. (2011) [15] | USA | Explored the reproductive health experiences and perceptions of African refugee women, and the nature of their relationship with healthcare providers in order to provide information to enhance the clinical encounters of refugees and health care providers. | Phoenix Arizona.  Healthcare providers interviewed at the Maricopa Medical Centre. | Qualitative study.  Postcolonial feminism, intersectionality, and human rights provided the  theoretical frameworks | Demographic questionnaire; in-depth semi-structured interviews and observation.  Purposive sampling | Thematic analysis | 30 interviews (20 African refugee women (between the ages of 18 and 55) and 10 health care providers. | 10 female health care providers (1 obstetrician/ gynaecologist, 2 health social workers, 2 certified nurse midwives, and 5 registered nurses) |
|  | Johansen, R. E. (2006) [16] | Norway | Explored Norwegian health care workers’ experience and management of birth care of women who have undergone inﬁbulation. | Norway - conducted in hospitals and clinics | Interviews and participant observation | In-depth interviews and case interviews on speciﬁc deliveries that were conducted immediately afterwards. Participant observation in a perinatal clinic and in a maternity ward.  Participants were recruited from 3 hospitals and 3 antenatal clinics with a high proportion of immigrant patients. | Not stated | 40 health workers | 40 health workers  (25 midwives, 9 gynaecologists, 3 general practitioners & 3 nurses) |
|  | Johansen, R. E. (2017) [17] | Norway | To explore the factors that encourage and hinder women and girls from seeking medicalized deinfibulation. | Norway | Qualitative study using interviews | In-depth interviews with women and men of Somali and Sudanese origin and health providers, recruited through formal channels based on their experience and work with FGM/ C and/or refugees. | Thematic analysis | 30 health service providers  66 women and men of Somali and Sudanese origin | 30 health professionals  (included employees from health clinics that conducted deinfibulation, school nurses, sexual counsellors for youth, and other refugee and asylum seeker personnel) |
|  | Lazar, J. N., Johnson-Agbakwu, C. E., Davis, O. I., & Shipp, M. P. L. (2013) [18] | USA | To obtain information about providers’ experiences, training, practices and attitudes surrounding the prenatal care, delivery, and management of women with FGM/C | Columbus, Ohio.  Setting was in hospitals’ cafeteria, in hospitals’ staff lounge, or in physicians’ offices. | Exploratory study | Semi -structured interviews.  Recruited via the hospital and clinics’ public websites in areas where Somalis are known to access care or/and through referral from interviewed physicians. | Content analysis | 14 obstetricians/ gynaecologists and nurse-midwives  Age range 30-70 years | 14 health providers  (9 obstetricians and 1 family practice physician who was Somali, 3 nurse midwives, and 1 nurse practitioner) |
|  | León-Larios, F., & Casado-Mejía, R. (2012) [19] | Spain | To explore the influence of midwives’ gender on knowledge, perception and approach to female genital mutilation. | Province of Seville at participants’ work centres. | Qualitative study. | Three focus group discussions.  Purposive sampling | Discourse analysis | 24 midwives from primary healthcare centres  Age range 29-57 years | 24 midwives |
|  | Leval, A., Widmark, C., Tishelman, C., & Maina Ahlberg, B. (2004) [20] | Sweden | To investigate midwives’ perceptions and attitudes toward circumcision and circumcised women and their experiences of providing care for them. | Sweden | Qualitative - using the anthropological concept of ethnocentrism. | Focus group discussions and interviews. | Thematic analysis | 26 midwives  Age range 37-53 years (all but one were born in Sweden) | 26 midwives |
|  | Moore, K. (2012) [21] | UK | To investigate how obstetric complications for women with FGM are currently managed in the UK and to elicit the opinions and experiences of midwives with regard to providing culturally competent care for women who have undergone FGM. | Across the UK | An exploratory study | Interviews conducted via Skype and telephone.  Recruited from websites and contacts | Principles of ‘Framework Analysis’ | 4 midwives  (all having experience of caring for women with FGM) | 4 midwives |
|  | Ogunsiji, O. (2015) [22] | Australia | Explored the knowledge and attitude of Australian midwives caring for women living with FGM. Part of a larger qualitative study that explored meaning of care and care-giving experiences of Australian midwives who are caring for women with FGM. | Sydney. New South Wales.  Setting was nearby university classrooms and public libraries. | Interpretive qualitative approach - as suggested by Heidegger | In-depth face to face interviews.  A generic e-mail containing the title and aim of the study was sent to expert midwives in a school of nursing and midwifery at an Australian University. | Constant comparison and identification of emerging themes. | 11 midwives  All female. Age range 25-60 years. | 11 midwives. |
|  | Ogunsiji, O. (2016) [23] | Australia | To report Australian midwives’ stories about how they manage obstetric care of women living with FGM. Part of the study that explored the experiences of Australian midwives caring for women living with FGM. | Sydney, New South Wales Australia. | Heideggerian qualitative interpretive approach. | Face-to-face, unstructured, in-depth, audio-taped interview.  Recruited from staff proﬁle of a university website in Sydney, Australia, and by snowball sampling | Analysis and identiﬁcation of emerging themes through selective or highlighting approach. | 11 midwives | 11 midwives, |
|  | Rubin, E. A. (2000) [24] | USA | Explored multiple cultural and psychological factors that influence how North American medical providers communicate with immigrant women who have been circumcised. | North America, USA | A qualitative-phenomenological research design influenced by psychodynamic and feminist theories of psychotherapy. | Face-to-face interviews using a semi-structured interview.  Purposeful, non-random sampling method. | By clustering the data from each participant into variables identified during the design of the study. | 10 semi-structured interviews with licensed medical providers. Two participants also worked in private practices  Age range 30-54 years | 10 female healthcare providers (5 nurse-midwives, 3 paediatricians, 1 internist, and 1 nurse practitioner) |
|  | Thierfelder, C. (2003) [25] | Switzerland | To let circumcised women speak for themselves about the Swiss health care system.  To find out what key health care providers of different professions think about the situation and to compare the two.  To analyse the attitudes, readiness and information needs of Swiss health care providers to treat genitally mutilated women. | Cantons of Geneva, Vaud, Zurich and Berne, Basel, Solothurn and Appenzell.  With the exception of two interviews, all in-depth-interviews with health care professionals were telephone interviews. | Qualitative design with triangulation of data sources. | Structured, in-depth interviews with different categories of health care professionals.  Purposeful sampling (snowball sampling, and sampling according to confirming/ disconfirming assumptions) were carried out. Targeted the professions most directly confronted with the phenomenon of FGM in Switzerland. | Content analysis | In-depth interviews (n=37) with Swiss health care providers  Sixty six individual interviews and focus group discussions with women (n=29) and men (n=3) of migrant communities from Somalia and Eritrea. | 37 health providers  (17 midwives, 20 physicians; 17 gynaecologists/ obstetricians, 3 GPs) |
|  | Vangen, S., Johansen, R. E. B., Sundby, J., Traeen, B., & Stray-Pedersen, B. (2004) [26] | Norway | To explore how perinatal care practice may inﬂuence labour outcomes among circumcised (Somali) women. | Oslo,  Interviewed in the conversation room at their working place. | Qualitative method drawing upon empowerment - and disempowerment theories. | Repeated in-depth interviews with the Somali immigrants and health care professionals  Recruited from the obstetric wards, the maternal and child health centres. | Analysed by the main themes and contrasting views on the various issues raised. | 59 participants (36 Norwegian health care professionals & 23 Somali immigrants) | 36 health care professionals.  (8 gynaecologists, 22 midwives, 3 public health doctors, and 3 public health nurses). |
|  | Vaughan, C., White, N., Keogh, L., Tobin, J., Ha, B., Ibrahim, M., & Bayly, C. (2014) [27] | Australia | To improve understanding of the impacts of FGC and to make suggestions for service development - by engaging with local women, communities and health providers | North Yarra area (Carlton, Collingwood and Fitzroy) in inner Melbourne Australia.  Interviewed at a time and location of their convenience. | Community-based qualitative study | Interviews with healthcare service providers  Focus group discussions, small group discussions and individual interviews with community members.  Recruitment from community consultations, community organisations and networks | Thematic analysis | 123 individuals  112 migrant men and women and 11 health providers | 11 health service providers  (a senior women’s health clinician, senior clinical midwife, 2 obstetrician/ gynaecologists, a GP, community midwife and 4 FARREP (community outreach) workers) |
|  | Vaughan, C., White, N., Keogh, L., Tobin, J., Murdolo, A., Quiazon, R., & Bayly, C. (2014) [28] | Australia | To identify the FGC-related service needs prioritised by affected communities living in Victorian regional centres, and to build evidence as to the training, education and professional development required for service providers working in regional Victoria to be able to meet these needs. | Australia Regional Victoria Ballarat, Geelong, Latrobe Valley and Shepparton, | Community based qualitative approach | Interviews with service providers  Focus group discussions with community members  Service providers were recruited from networks of the Multicultural Centre for Women’s Health and the Royal Women’s Hospital | Thematic analysis | 15 health service providers  51 migrant community members 950 women, 1 man) | 15 health service providers  (3 medical consultants, 4 GPs, 1 senior midwife, 1 sexual health practitioner, 1 midwife, 3 refugee health workers, community health worker and a community development worker) |
|  | Widmark, C., Leval, A., Tishelman, C., & Ahlberg, B. M. (2010) [29] | Sweden | To explore obstetricians’ perspectives on caring for women with FGC in Sweden: how do they describe, explain and reason about care and policies? | Sweden | A qualitative study | Open-ended, semi-structured interviews, from a purposeful sample. | Analysis was based on tenets of interpretive description | 20 participants  (1 interview excluded due to technical error) | 19 obstetricians  (13 senior obstetricians & 7 senior house ofﬁcers) |
|  | Widmark, C., Tishelman, C., & Ahlberg, B. M. (2002) [30] | Sweden | To investigate Swedish midwives’ experiences of caring for inﬁbulated women, midwives’ perceptions and attitudes towards inﬁbulation and inﬁbulated women, the education and training provided to enable midwives to care for and deliver inﬁbulated women, concerns and problems in providing care and ﬁnally, strategies employed by midwives to deal with dilemmas that may occur. | Central Sweden in quiet rooms at the midwives’ workplace, and some at the researchers’ workplace. | Qualitative. Drew upon principles from Guba and Lincoln’s ‘fourth generation evaluation’. | Three focus group discussions and 3 individual interviews.  Recruited using a multi-stage sampling procedure. | Grouping of all meaning bearing units relevant to the research, followe3d by thematic analysis | 26 midwives  Age range 37-53 years | 26 midwives |

**References of Included Studies**

1. Abdi R. Carving culture: Creating identity through female genital cutting. Durham Anthropology Journal. 2012;18(1):115-53.

2. Behrendt A. Listening to African Voices: Female Genital Mutilation/Cutting among Immigrants in Hamburg: Knowledge, Attitudes and Practice. Hamburg, Germany: Plan; 2011.

3. Bergqvist H, Svensson J. [Midwives Experiences of Encounters wth Young Women who come from Areas Where the Practice of Genital Mutilation is Common] Barnmorskors Erfarenheter Av Möten Med Unga Kvinnor Som Kommer Från Områden Där Kvinnlig Könsstympning är Aanligt Förekommande. MSc Thesis, Sweden: University of Skövde; 2016.

4. Bibi N, Rahimian N. [Nurses’ Experience and Knowledge about Female Genital Mutilation] Sjuksköterskans Erfarenheter Och Kunskaper Om Kvinnlig Könssympning. MSc Thesis, Sweden: Sophiahemmet University; 2013.

5. Brodin E, Mårtensson N. [District Nurses' Knowledge and Experience of Female Genital Mutilation] Distriktssköterskors Kunskap Och Erfarenhet Av Kvinnlig Könsstympning. MSc Thesis, Sweden: University of Örebro; 2016.

6. Bulman KH, McCourt C. Somali refugee women's experiences of maternity care in west London: a case study. Crit Public Health. 2002;12(4):365-80.

7. Bulman K, McCourt C. Report on Somali Womens' Experiences of Maternity Services. London, UK: Centre for Midwifery Practice, Wolfson Institute of Health Sciences, Thames Valley University and Hammersmith Hospitals National Health Service Trust; 1997.

8. Burchill J, Pevalin DJ. Demonstrating cultural competence within health-visiting practice: working with refugee and asylum-seeking families. Divers Equal Health Care. 2014;11(2):151-9.

9. Byrskog U, Olsson P, Essen B, Allvin MK. Being a bridge: Swedish antenatal care midwives' encounters with Somali-born women and questions of violence - a qualitative study. BMC Pregnancy Childbirth. 2015;15(1).

10. Dawson AJ, Turkmani S, Varol N, Nanayakkara S, Sullivan E, Homer CS. Midwives' experiences of caring for women with female genital mutilation: insights and ways forward for practice in Australia. Women Birth. 2015;28(3):207-14.

11. Fawcett L. Somali Refugee Women and their U.S. Healthcare Providers: Knowledge, Perceptions and Experiences of Childbearing [Doctor of Philosophy]. PhD Thesis, USA: Arizona State University; 2014.

12. Gertsson M, Serpan H. [Meeting with the Unthinkable: Value Conflicts in Meetings with Women Vulnerable to Female Genital Mutilation] Mötet Med Det Otänkbara: Värdekonflikter I Mötet Med Kvinnor Utsatta för Kvinnlig Könsstympning. MSc Thesis, Sweden: University of Kalmar; 2009.

13. Holm L, Kammensjö H. [School Nurses' Experiences of Female Genital Mutilation among Girls] Skolsköterskors Upplevelser Kring Kvinnlig Könsstympning Bland Flickor. MSc Thesis, Sweden: University of Skövde; 2012.

14. Hussen MA. Services for Women with Female Genital Mutilation in Christchurch: Perspectives of Women and their Health Providers. MSc Thesis, New Zealand: University of Canterbury; 2014.

15. Jatau M. Living Between Two Cultures: A Reproductive Health Journey of African Refugee Women [Doctor of Philosophy]. PhD Thesis, USA: Arizona State University; 2011.

16. Johansen RE. Care for infibulated women giving birth in Norway: an anthropological analysis of health workers' management of a medically and culturally unfamiliar issue. Med Anthropol Q. 2006;20(4):516-44.

17. Johansen RE. Virility, pleasure and female genital mutilation/cutting: a qualitative study of perceptions and experiences of medicalized defibulation among Somali and Sudanese migrants in Norway. Reprod Health. 2017;14(1):25.

18. Lazar JN, Johnson-Agbakwu CE, Davis OI, Shipp MPL. Providers' perceptions of challenges in obstetrical care for Somali women. Obstet Gynecol Int. 2013;2013:149640.

19. León-Larios F, Casado-Mejía R. [Influence of gender on knowledge, perception and approach to harmful traditional practices: female genital mutilation]. Evidentia. 2012;9(40):1-7.

20. Leval A, Widmark C, Tishelman C, Maina Ahlberg B. The encounters that rupture the myth: contradictions in midwives' descriptions and explanations of circumcised women immigrants' sexuality. Health Care Women Int. 2004;25(8):743-60.

21. Moore K. Female Genital Mutilation and Cultural Competency: Moving Towards Improved Management of Obstetric Care [Masters Dissertation]. MSc Thesis, Edinburgh: Queen Margaret University; 2012.

22. Ogunsiji O. Female genital mutilation (FGM): Australian midwives’ knowledge and attitudes. Health Care Women Int. 2015;36(11):1179-93.

23. Ogunsiji O. Australian midwives' perspectives on managing obstetric care of women living with female genital circumcision/mutilation. Health Care Women Int. 2016;37(10):1156-69.

24. Rubin EA. When Cultures Collide: An Exploration of Cultural Competence and Cross-Cultural Communication between American Medical Providers and Immigrant Women who have been Circumcised. PhD Thesis, USA: University of Massachusetts; 2000.

25. Thierfelder C. Female Genital Mutilation and the Swiss Health Care System. PhD Thesis, Switzerland: University of Basel; 2003.

26. Vangen S, Johansen REB, Sundby J, Traeen B, Stray-Pedersen B. Qualitative study of perinatal care experiences among Somali women and local health care professionals in Norway. Eur J Obstet Gynecol Reprod Biol. 2004;112(1):29-35.

27. Vaughan C, White N, Keogh L, Tobin J, Ha B, Ibrahim M, et al. Listening to North Yarra Communities about Female Genital Cutting. Melbourne, Australia: The University of Melbourne; 2014. Contract No.: ISBN 978 0 9925013 0 3.

28. Vaughan C, White N, Keogh L, Tobin J, Murdolo A, Quiazon R, et al. Female Genital Mutilation/Cutting in Regional Victoria. Research to Practice. Melbourne, Australia: The University of Melbourne; 2014. Report No.: 1470-0328 Contract No.: ISBN 978 0 9925013 1 0.

29. Widmark C, Leval A, Tishelman C, Ahlberg BM. Obstetric care at the intersection of science and culture: Swedish doctors' perspectives on obstetric care of women who have undergone female genital cutting. J Obstet Gynaecol. 2010;30(6):553-8.

30. Widmark C, Tishelman C, Ahlberg BM. A study of Swedish midwives' encounters with infibulated African women in Sweden. Midwifery. 2002;18(2):113-25.
