# Supplementary material for: Crossing cultural divides: A qualitative systematic review of factors influencing the provision of healthcare related to female genital mutilation from the perspective of health professionals
Source: PLoS One. 2019 Mar 4;14(3):e0211829. doi: 10.1371/journal.pone.0211829 (PMC6398829; doi:10.1371/journal.pone.0211829)
Supplement: S8 Table — (DOCX) [file pone.0211829.s008.docx]

**S9 CERQual Assessment and Summary of Findings Table**

| **Summary of review finding** | **Studies coded to finding** | **Methodological limitations** | **Coherence** | **Adequacy** | **Relevance** | **CERQual assessment** | **Explanation of CERQual score** |
| --- | --- | --- | --- | --- | --- | --- | --- |
| **Analytical theme 1: knowledge and training** | | | | | | | |
| **1.1 Knowledge and awareness**  Healthcare providers had variable knowledge around FGM/C, depending on their work setting, role, exposure to FGM/C and exposure to training. Many studies described a lack of provider awareness around FGM/C or provider reports of having insufficient, inaccurate or partial knowledge and skills, leading to misconceptions, lack of awareness, fear and uncertainty about how to talk about FGM/C and how to support women with FGM/C. | n=25  [1-25] | Moderate concerns  Some papers (n=9) have limitations related to no stated paradigm, unclear methodology, and no or partial reflexivity. Two studies have serious concerns from lacking ethics description, no reflexivity and weak methods (n=2). However, the majority of studies pose minor concerns related to no stated paradigm, and partial reflexivity (n=14). | No concerns | Minor concerns  25 papers report on this theme, The majority of studies contribute rich data to this theme (n=17). | Minor concerns  A good mix of cadres of staff and contexts. More studies among midwives only (n=9), with the majority conducted in Sweden (n=8). Only 3 studies were conducted in UK. The majority (n=19) of the studies were published in the last 10 years. | **High Confidence** | There are moderate methodological concerns, but a large number of studies consistently report this theme. There is rich data from a mixture of contexts and participants. |
| **1.2 Education and training**  Practitioners expressed a perceived need for greater education and training in all aspects associated with the management of women and girls with FGM/C. Health providers identified a lack of basic (pre-service) education (or an input that was too brief and superficial) and a need for regular in-depth CPD around FGM/C that included all aspects of FGM/C (e.g. communication, sexual and cultural issues, as well as clinical management), practical skill development and access to mentorship and clinical supervision where relevant. | n=21  [1, 3, 4, 6, 8, 9, 13, 14, 17, 18, 20-27] | Moderate concerns  Two papers pose serious concerns arising from poor methodology and unclear processes, but the majority are high quality studies (n=11). Some pose moderate concerns due to no stated paradigm, and partial or no reflexivity (n=8). | No concerns | Minor concerns  The majority of studies report this theme (n=21). Of these, 14 present rich data towards this theme. | Minor concerns  There is a mix of contexts involving 9 countries and 7 participant groups (different cadres and roles). Six studies have mixed groups of health professionals. The majority (n=17) of the studies were published in the last 10 years. | **High Confidence** | There are moderate methodological concerns, but a large number of studies consistently report this theme. There is rich data from a mixture of contexts and participants. |
| **Analytical theme 2: communication is key** | | | | | | | |
| **2.1 Language barriers and interpretation challenges**  Language barriers and problems with accessing suitable interpretation significantly compromised health provider’s ability to provide appropriate FGM/C-related care. It affected health provider’s ability to ask questions about FGM/C or to provide relevant information. This was particularly an issue with women who were still relatively recent migrants. | n=20  [1-6, 8, 10, 13, 14, 16, 17, 20, 22-25, 28, 29] | Moderate concerns  The majority of studies are of good quality (n=12), with moderate concerns due to unclear methods, no paradigm and partial reflexivity (n=6), and two studies with more serious concerns related to lack of ethics statements and very poor methodology. | No concerns | No concerns  Two thirds of papers report this theme. The majority of these present rich data to illuminate the theme (n=15). | No concerns  Well distributed studies covering several different countries and a range of professional groups. | **High Confidence** | There are moderate methodological concerns. However two thirds of studies report this theme. Most of the data is rich, representing a mixture of contexts and participants. |
| **2.2 Talking about a sensitive topic**  As a sensitive and taboo topic, health providers found it difficult to talk about FGM/C with patients. Sometimes this was a result of trying to show culturally sensitivity and not offend patients, and sometimes it was because the health providers felt awkward and embarrassed. Open communication was seen to require trust, time and experience, and some health providers felt that routinizing FGM/C-related questions would be useful. | n=24  [2-11, 13-17, 19-23, 25, 26, 28, 29] | Moderate concerns  The majority of studies contributing to this theme are high/medium quality (n=15). There are moderate concerns due to partial or no reflexivity and no stated paradigm. One paper has more serious concerns due to very poor methods. | No concerns | No concerns  24 studies report on this theme with the majority contributing rich data (n=15). | Minor concerns  One third of the studies are from Sweden (n=8). Nine papers involve midwives only or other obstetric care providers (n=4). However, there are 7 other countries and other cadres also represented. | **High Confidence** | There are moderate methodological concerns, but a large number of studies consistently report this theme. There is rich data from a mixture of contexts and participants. |
| **2.3 Women also find FGM/C hard to talk about**  Health providers perceived that women rarely proactively mentioned FGM/C in the context of a consultation. Providers attributed this to cultural taboos within women’s own societies, feeling ashamed and embarrassed or being fearful of being judged. | n=13  [3, 6, 8, 9, 13, 16, 19-23, 28, 29] | Moderate concerns  Seven studies are high quality, five are medium and one is low quality. There are moderate concerns in the latter groups due to poor reflexivity and unclear description of methodology and methods. | Moderate concerns  The data presents providers’ perceptions of this issue, hence the conclusions are largely inferred. | Moderate concerns  Thirteen studies report this theme. Only six papers present rich data – the others provide a more superficial description. | Moderate concerns  All studies are from non-UK contexts (Sweden, USA and Australia). Four of the studies focus exclusively on midwives but other groups are also represented. | **Moderate confidence** | There are moderate concerns in all the domains. |
| **Analytical theme 3: encountering the ‘other’ in clinical practice: negotiating cultural dissonance and achieving cultural understanding** | | | | | | | |
| **3.1 Attitudes towards FGM/C: mixed emotions**  FGM/C elicited strong emotional reactions in providers. Many providers expressed negative reactions to FGM/C, including shock, disgust and horror. FGM/C exemplified a profound cultural difference between host providers and migrant patients, with women’s bodies seen to be ‘different’ and mutilated. At the same time, providers expressed empathy and support for affected women and saw them as victims of a violent or patriarchal culture. Some providers who had travelled or who were more experienced expressed a more nuanced position, where FGM/C was seen as a practice that was different, but that needed to be understood in its own context. | n=17  [2, 3, 6-8, 11, 13, 15-18, 20-22, 24, 25, 29, 30] | Moderate concerns  One paper poses serious concerns due to poor methods, no reflexivity. Moderate concerns in five papers due to lack of discussion of reflexivity which may be particularly salient for this theme. | No concerns | Minor concerns  17 studies report on this theme. Data is generally rich/in-depth. Only three papers contribute thin data. | Minor concerns  The majority of studies reporting this theme are among midwives (n=7). However seven studies with mixed groups also report the theme. Six of the studies are from Sweden, but a range of other countries are also represented. Eleven studies were published in the last 10 years. | **High confidence** | There are moderate methodological concerns, but a large number of studies consistently report this theme. There is rich data from a mixture of contexts and participants. |
| **3.2 Cultural dissonance – control and resistance in clinical encounters**  Studies reported cultural stereotyping and misunderstandings between patients and providers to be common. Some health providers held stereotyped views of FGM/C affected women leading to a failure to address individual needs. Studies described how health providers become perplexed that clients sometimes did not follow their advice and sought to exert control over the clinical situation. Some patients resisted such medical dominance or challenged stereotypes, leading to misunderstandings and miscommunication (especially around caesarean sections and episiotomies) which in turn led to sub-optimal care and/or sub-optimal clinical outcomes. | n=20  [3, 5-8, 10-16, 20-25, 28, 29] | Moderate concerns  Most studies pose no major methodological concerns. There are moderate concerns in six papers due to due to lack of discussion of reflexivity and theoretical stance which may be particularly salient in this theme. | No concerns | No concerns  Two thirds of studies report this theme (n=20). The majority provide relatively rich data with only two studies providing thin data regarding this finding. | Moderate concerns  Almost one third of studies are from Swedish contexts (n=7) and the majority are related to pregnancy or childbirth as most papers are on experiences from midwives (n=6) and obstetricians (n=6). But other studies also provide data on other countries and contexts and other groups. Two thirds of the papers (n=13) were published in the last 10 years. | **Moderate confidence** | Moderate confidence due to methodological concerns and concerns about relevance with the majority of studies related to the maternity care context. |
| **3.3 Acknowledging the role of the family**  Providers reported that families and husbands played a strong role in healthcare decision making related to FGM/C (especially around caesarean section, timing of deinfibulation and preferences around reinfibulation). The need for family involvement was sometimes perceived as frustrating as it could delay decision making but was recognised as an essential part of effective care. | n=15  [3, 4, 6, 11, 13-17, 19, 20, 24, 25, 27, 29] | Moderate concerns  Most papers are high (n=8) or medium quality (n=4). There are moderate concerns about poor reporting of reflexivity in 7 studies which may be particularly salient in this theme. There are serious concerns with 3 papers which have low quality due to lack of methodological reporting, no reflexivity and poor discussion of ethics. | Minor concerns  Some of the data appears to be based on on assumptions, whereas other data is based on examples from practice. | No concerns  Half of the studies report this theme. The majority (n=14) present medium/high ‘rich’ data. | Minor concerns  One third of studies (n=5) are from Sweden, and one third are with midwives only (n=5). However, the other studies report a mix of contexts and cadres. | **High confidence** | There are moderate methodological concerns, but half of the studies report this theme and there is rich data from a mixture of contexts and participants. |
| **3.4 Gender of the provider**  Health providers reported a strong perception that women from FGM/C affected communities preferred to be seen by a female health professional and that a provider’s ability to form an open and trusting relationship with their patients was more likely if they were female. The gender of the provider was said to affect women’s willingness to seek help, to talk openly to the practitioner and to be examined. | n=8  [4, 10, 14, 16, 19, 20, 23, 25] | Minor concerns  Seven studies are classified as high quality and one is medium. Minor concerns due to lack of reflexivity, however this is considered to be of less significance for this theme. | Moderate concerns  Some of the data appears to be based on on assumptions and inference rather than due to having discussed the issue with women themselves. | Minor concerns  Eight studies report on this theme. Six studies contribute rich data. | Moderate concerns  Studies from 5 countries (all non-UK). Three studies specifically relate to the maternity care context. The other studies reflect a range of healthcare providers. | **Moderate confidence** | Moderate confidence due to moderate concerns in two domains (relevance, coherence). |
| **3.5 Crossing the cultural divide – strategies and elements of culturally sensitive care**  Many papers described strategies and approaches to build good relationships with FGM/C-affected women and thus to cross cultural divides and provide appropriate care. Key features were an understanding of FGM/C in the context of women’s wider needs and culture (i.e. being person centred as well as ‘culturally’ sensitive), and taking time to build rapport and trust. Pre-requisites were knowledge, time, communication, a non-judgemental, open attitude and family involvement. Culturally sensitive care was particularly discussed by midwives and nurses. | n=23  [1-10, 13, 16-20, 22-26, 28, 29] | Moderate concerns  The majority of studies are high (n=11) or medium quality (n=10). There are moderate concerns in six studies due to lack of discussion of reflexivity. | No concerns | Minor concerns  This theme is reported across the majority of papers in the review (n=23) and 14 studies contribute very rich data. | Minor concerns  The studies are mainly from Sweden (n=8) and Australia (n=5) with minor concerns as they are mostly among midwives (n=9). However, other cadres are also represented. | **High confidence** | There are moderate methodological concerns, but over two thirds of the studies report this theme and there is rich data from a mixture of contexts and participants. |
| **Analytical theme 4: identifying FGM/C** | | | | | | | |
| **4.1 Presentation and help seeking**  Providers reported that women primarily seek care related to FGM/C when symptomatic or for pregnancy. Women may not link their attendance reason to FGM/C and may not mention FGM/C unless the provider asks. Given that many medical procedures and consultations do not require physical examination, FGM/C may not be identified at all unless the provider specifically asks. Women were reported to be reluctant to raise the subject themselves. The exception was where specialist services or experienced providers exist in which case women may seek them out via family and social networks. The main focus of provider’s attention was on gynaecological symptoms and the maternity context. They generally did not ask about sexual problems or psychological problems and women rarely raised these issues. | n=15  [1-4, 6, 7, 9, 14, 19, 20, 22, 23, 25, 29, 30] | Moderate concerns  Out of 15 papers, 8 are high quality, 6 are medium quality and 1 is low quality. Five papers in particular present moderate concerns due to lack of reflexivity and partial/unclear reporting of participants’ voices. The other papers present minor or no concerns. | Moderate concerns  Not all papers report all aspects of this finding. | Minor concerns  Half of the papers in the review report this theme (n=15). All papers contribute rich or relatively rich data. | Moderate concerns  One third of the studies are from Sweden (n=5) with only one UK study. Over one third of papers (n=6) represent midwives’ views specifically. However other cadres of health professional are represented in other papers. This finding relates primarily to the maternity care or primary care context. The majority of the papers (n=12) were published in the last 10 years. | **Moderate confidence** | Moderate confidence due to moderate concerns regarding methodology, relevance and coherence. |
| **4.2 Practices and processes around identifying FGM/C**  Organisational and system-level mechanisms to identify FGM/C were reported to be inconsistent and uncoordinated. A key barrier to early and appropriate identification of FGM/C was that providers did not ask about FGM/C, but also, that organisational systems and processes were often not adequately set up to prompt them to ask or to ensure that follow up would occur. Organisational and system barriers to FGM/C identification were related to the existence (or not) of clear guidelines, procedures and referral pathways, record keeping processes and to the need for clarity of roles and responsibilities and communication/coordination between organisational units and professional groups. | n=20  [2-6, 9-11, 13, 14, 16, 17, 19-25, 28] | Moderate concerns  There are serious concerns regarding 1 study related to poor methods, partial ethics and no reflexivity. There are moderate concerns in 6 studies related to no paradigm, unclear analysis and partial reflexivity. The rest of the studies have minor concerns related to lack of reflexivity. | Moderate concerns  Not all papers report all aspects of this finding. | Minor concerns  20 papers describe this theme with rich data from over half of these (n=12) and moderately rich data (n=6) with only 2 papers having thin findings. | Minor concerns  Several studies conducted in Sweden (n=6) and among midwives (n=7), but with a good representation among other professionals and 7 other countries. Fifteen of the 20 papers were published in the last 10 years. | **Moderate confidence** | Moderate confidence due to moderate concerns regarding methodology and coherence. |
| **Analytical theme 5: clinical management practices: inconsistent and variable** | | | | | | | |
| **5.1 Deinfibulation timing**  The majority of providers reported that women preferred deinfibulation to be done in second stage of labour (rather than antenatally) and that these decisions were influenced by the wider community. In contrast, most providers preferred deinfibulation to be undertaken antenatally (although some felt this might be an unnecessary additional trauma for the woman), but in most cases, the usual practise was to undertake intrapartum deinfibulation. | n=8  [6, 17, 19-21, 23-25] | Moderate concerns  The majority of the papers are high (n=6) or medium quality (n=2), and one was low quality with serious concerns due to poor reporting of ethics and poor reflexivity. | No concerns | Minor concerns  This theme is reported by relatively few of the review studies (n=8). However, all present rich data. | Moderate concerns  Half of the studies reporting this theme are among midwives (n=4). Other studies include mixed cadres (n=2), nurses (n=1) and doctors (n=1). The studies represent 5 different countries. All the studies refer to deinfibulation only in the context of maternity care. | **Moderate confidence** | Moderate confidence due to moderate concerns regarding relevance as no studies report on this theme outside of the maternity context. |
| **5.2 Deinfibulation practice**  Providers in several studies (especially midwives), expressed uncertainty regarding the best way to manage deinfibulation, there was variability in practise and caesarean sections were sometimes performed too hastily (particularly in the US context) in order to avoid having to undertake a deinfibulation. Uncertainty was compounded by difficulties in being able to undertake examinations or foetal monitoring to determine progress of labour. Midwives in particular expressed fear and stress when dealing with women with type III FGM/C due to their lack of familiarity with deinfibulation and uncertainty over how best to manage it. | n=21  [2-11, 13, 14, 16, 17, 19-25] | Moderate concerns  The studies are a mix of high (n=10), medium (n=9) and low (n=2) quality. There are moderate concerns particularly in 7 studies due to poor description of methods processes, no paradigm, and no reflexivity. | Moderate concerns  Not all papers report all aspects of this finding. | Moderate  concerns  Many studies report on this theme (n=21) with rich data from over half of these studies (n=11) but the data in 10 studies is quite ‘thin. | Minor concerns  The majority of studies discuss this finding in relation to maternity care contexts. Papers from 7 different countries report this finding. Eighteen of the 21 papers were published in the last 10 years. | **Moderate confidence** | Moderate confidence due to moderate concerns regarding methodology, adequacy and coherence. |
| **5.3 Reinfibulation ambivalence**  Studies reported variable attitudes towards, and practise of, reinfibulation. Practitioners were generally aware that reinfibulation is illegal but reinfibulation was reported in three studies (all over 10 years old). Some practitioners reported moral ambivalence about refusing to reinfibulate a client if she was clearly requesting it, as refusal of a patient’s request was seen to contradict the principles of person-centred care. Practitioners felt it was important to involve husbands in discussions around reinfibulation. | n=11  [6, 7, 11, 15, 17-21, 24, 25] | Moderate concerns  Most studies relating to this theme are high (n=6) or medium quality (n=4) and one is low quality. However, there are moderate concerns in 7 studies relating to no or partial discussion of reflexivity. | Moderate concerns  Not all papers report all aspects of this finding. | Minor concerns  Nine of the 11 papers provide rich data towards this theme. | Moderate concerns  All papers relate to the maternity context. Six studies are exclusively with midwives. The studies represent five countries. However, 5 of the papers were over 10 years old, therefore it is not clear if their findings are still relevant. | **Moderate confidence** | Moderate confidence due to moderate concerns regarding methodology, coherence and relevance. |
| **5.4 Need for guidelines**  Many studies reported a lack of (or lack of awareness of clinical guidelines to direct practice around deinfibulation timing, procedure and reinfibulation. Providers reported a need for guidelines to establish clarity and consistency in clinical practice. | n=15  [2, 4-6, 8, 13, 14, 17, 19-25] | Moderate concerns  There are moderate concerns in 5 papers due to unclear methods, partial ethics and no reflexivity, and serious concerns related to one paper due to weak methodology, unclear processes and partial ethics (n=1). The rest of the studies are of minor (n=7) to very minor (n=2) concerns due to no paradigm and partial reflexivity. | No concerns | Minor concerns  15 papers report on this theme, with the majority presenting rich data (n=12). | Moderate concerns  The majority of papers relate to the maternity care context. There are studies from 7 different countries. Four of the papers are over 10 years old. | **Moderate confidence** | There are moderate concerns regarding methodological limitations and relevance. |
| **5.5 Psychological issues**  Several studies highlighted provider’s experiences that women could experience emotional difficulties in relation to their FGM/C and that clinical interventions in particular could be traumatic for women in terms of inducing flashbacks from the original procedure and heightening pain. Practitioners emphasised the importance of providing counselling and psychological support. These aspects were mainly mentioned by nurses/midwives. Although recognised as a need, in most of the studies reporting this theme, the actual availability of counselling or psychological support as a routine part of care was not clear. | n=12  [2, 5, 11] [1, 3, 4, 6, 16, 19-22] | Moderate concerns  Amongst the studies, 6 are high quality, 5 are medium quality and 1 is low quality. There are moderate concerns related to poor reporting of reflexivity and partial reporting of participants’ voices. | Moderate concerns  Not all papers report all aspects of this finding. Some of the findings appear to be inferred rather than based on in-depth descriptions of experiences with women. | Moderate concerns  Just over one third of studies report on this theme. Only six of these provide rich data. | Minor concerns  Six countries are represented and the studies reflect a range of health professionals. | **Moderate confidence** | Moderate confidence due to moderate concerns regarding methodological limitations, coherence and adequacy. |
| **Analytical theme 6: optimal service development for FGM/C care** | | | | | | | |
| **6.1 Provider’s role in prevention**  Apart from school nurses whose role explicitly encompasses safe guarding and sexual health, relatively few studies reported practitioners addressing prevention as part of their FGM/C-related care, although several studies reported that this *should* be part of any practitioners’ role, including GPs. Prevention discussions appeared to take place in an ad-hoc way (dependent on individual providers) rather than as a routinized aspect of care. Barriers to initiating prevention discussions in a clinical setting included: lack of time, inappropriate timing, feeling that the prevention discussion is someone else’s role, not having enough knowledge or confidence, feeling unsure if women’s responses could be trusted, lack of privacy and language barriers. | n=13  [1, 3, 4, 6-8, 12, 15, 17, 20-22, 25] | Moderate concerns  Studies were a mix of high (n=7), medium (n=4) and low (n=2) quality. Moderate concerns for several studies (n=6) due to lack for clarity in methods and lack of reflexivity and serious concerns in 2 studies due to lack of ethics reporting. | Moderate concerns  Not all papers report all aspects of this finding | Serious concerns  Over one third of studies contributed to this theme. However, the majority (n=8) contributed relatively thin data. | Minor concerns  Most studies are from Sweden (n=6) and in maternity settings (n=7). The other studies were from 4 other countries and among mixture of professionals. | **Low**  **confidence** | Low confidence due to moderate concerns regarding methodological limitations, coherence and serious concerns regarding adequacy. |
| **6.2 Community engagement and education**  Practitioners identified a need for greater education and awareness raising amongst affected communities, both on FGM/C itself and associated services but also on prevention. They suggested this should include men/boys as well as women/girls and identified a need for better information resources for community engagement. Practitioners also suggested that community engagement needed to be built on relationships of trust and concern for a community’s other needs (not just FGM/C) and some suggested that community outreach/liaison roles might be beneficial. | n=11  [1, 12] [3, 6-9, 17, 20, 22, 23] | Moderate concerns  Study quality was mixed (high n=4, medium n=5 and low n=2). Moderate concerns in 4 papers due to partial participant voices, no paradigm and unclear processes, while serious concerns (n=2) due to weak methods and unclear ethics processes. | No concerns | Minor concerns  Just over one third of studies in the review reported this theme. The majority provided relatively rich data. Three contributed only thin data. | Minor concerns  Studies were distributed across 7 countries, mainly Sweden (n=3) and Australia (n=3) and among several different cadres. Nine of the papers were published in the last decade. Nine of the papers focused on the maternity context. | **High confidence** | High confidence due to no/minor concerns in three domains (adequacy, relevance, coherence) and only moderate concerns regarding methodological limitations. |
| **6.3 Specialist services**  In terms of service development, providers (especially in lower prevalence areas) valued and recommended having specialist centres for holistic FGM/C management, counselling and prevention activities, with links to affected communities. Ensuring equity of access to services was a key concern. | n=9  [3, 6, 9, 17, 20, 22, 23, 25, 28] | Moderate concerns  Study quality was mixed (high n=3, medium n=4 and low n=2).  The majority of studies have minor concerns (n=7) due to no stated paradigm and partial reflexivity. Moderate concerns (n=1) and serious concerns (n=1) due to partial ethics descriptions, poor methods and no reflexivity. | Moderate concerns  Not all papers report all aspects of this finding | Moderate concerns  Due to relatively few studies reporting on this theme (9/30), and most contribute thin data. | Minor concerns  Studies reflect 5 different countries, mainly Sweden (n=3) and Australia (n=3). A good mix of different cadres, with slightly more midwives (n=4) | **Moderate confidence** | Moderate concerns regarding methodological limitations, coherence and adequacy. |

**References**

1. Behrendt A. Listening to African Voices: Female Genital Mutilation/Cutting among Immigrants in Hamburg: Knowledge, Attitudes and Practice. Hamburg, Germany: Plan; 2011.

2. Bergqvist H, Svensson J. [Midwives Experiences of Encounters wth Young Women who come from Areas Where the Practice of Genital Mutilation is Common] Barnmorskors Erfarenheter Av Möten Med Unga Kvinnor Som Kommer Från Områden Där Kvinnlig Könsstympning är Aanligt Förekommande. MSc Thesis, Sweden: University of Skövde; 2016.

3. Bibi N, Rahimian N. [Nurses’ Experience and Knowledge about Female Genital Mutilation] Sjuksköterskans Erfarenheter Och Kunskaper Om Kvinnlig Könssympning. MSc Thesis, Sweden: Sophiahemmet University; 2013.

4. Brodin E, Mårtensson N. [District Nurses' Knowledge and Experience of Female Genital Mutilation] Distriktssköterskors Kunskap Och Erfarenhet Av Kvinnlig Könsstympning. MSc Thesis, Sweden: University of Örebro; 2016.

5. Bulman KH, McCourt C. Somali refugee women's experiences of maternity care in west London: a case study. Crit Public Health. 2002;12(4):365-80.

6. Dawson AJ, Turkmani S, Varol N, Nanayakkara S, Sullivan E, Homer CS. Midwives' experiences of caring for women with female genital mutilation: insights and ways forward for practice in Australia. Women Birth. 2015;28(3):207-14.

7. Gertsson M, Serpan H. [Meeting with the Unthinkable: Value Conflicts in Meetings with Women Vulnerable to Female Genital Mutilation] Mötet Med Det Otänkbara: Värdekonflikter I Mötet Med Kvinnor Utsatta för Kvinnlig Könsstympning. MSc Thesis, Sweden: University of Kalmar; 2009.

8. Holm L, Kammensjö H. [School Nurses' Experiences of Female Genital Mutilation among Girls] Skolsköterskors Upplevelser Kring Kvinnlig Könsstympning Bland Flickor. MSc Thesis, Sweden: University of Skövde; 2012.

9. Hussen MA. Services for Women with Female Genital Mutilation in Christchurch: Perspectives of Women and their Health Providers. MSc Thesis, New Zealand: University of Canterbury; 2014.

10. Jatau M. Living Between Two Cultures: A Reproductive Health Journey of African Refugee Women [Doctor of Philosophy]. PhD Thesis, USA: Arizona State University; 2011.

11. Johansen RE. Care for infibulated women giving birth in Norway: an anthropological analysis of health workers' management of a medically and culturally unfamiliar issue. Med Anthropol Q. 2006;20(4):516-44.

12. Johansen RE. Virility, pleasure and female genital mutilation/cutting: a qualitative study of perceptions and experiences of medicalized defibulation among Somali and Sudanese migrants in Norway. Reprod Health. 2017;14(1):25.

13. Lazar JN, Johnson-Agbakwu CE, Davis OI, Shipp MPL. Providers' perceptions of challenges in obstetrical care for Somali women. Obstet Gynecol Int. 2013;2013:149640.

14. León-Larios F, Casado-Mejía R. [Influence of gender on knowledge, perception and approach to harmful traditional practices: female genital mutilation]. Evidentia. 2012;9(40):1-7.

15. Leval A, Widmark C, Tishelman C, Maina Ahlberg B. The encounters that rupture the myth: contradictions in midwives' descriptions and explanations of circumcised women immigrants' sexuality. Health Care Women Int. 2004;25(8):743-60.

16. Fawcett L. Somali Refugee Women and their U.S. Healthcare Providers: Knowledge, Perceptions and Experiences of Childbearing [Doctor of Philosophy]. PhD Thesis, USA: Arizona State University; 2014.

17. Moore K. Female Genital Mutilation and Cultural Competency: Moving Towards Improved Management of Obstetric Care [Masters Dissertation]. MSc Thesis, Edinburgh: Queen Margaret University; 2012.

18. Ogunsiji O. Female genital mutilation (FGM): Australian midwives’ knowledge and attitudes. Health Care Women Int. 2015;36(11):1179-93.

19. Ogunsiji O. Australian midwives' perspectives on managing obstetric care of women living with female genital circumcision/mutilation. Health Care Women Int. 2016;37(10):1156-69.

20. Thierfelder C. Female Genital Mutilation and the Swiss Health Care System. PhD Thesis, Switzerland: University of Basel; 2003.

21. Vangen S, Johansen REB, Sundby J, Traeen B, Stray-Pedersen B. Qualitative study of perinatal care experiences among Somali women and local health care professionals in Norway. Eur J Obstet Gynecol Reprod Biol. 2004;112(1):29-35.

22. Vaughan C, White N, Keogh L, Tobin J, Ha B, Ibrahim M, et al. Listening to North Yarra Communities about Female Genital Cutting. Melbourne, Australia: The University of Melbourne; 2014. Contract No.: ISBN 978 0 9925013 0 3.

23. Vaughan C, White N, Keogh L, Tobin J, Murdolo A, Quiazon R, et al. Female Genital Mutilation/Cutting in Regional Victoria. Research to Practice. Melbourne, Australia: The University of Melbourne; 2014. Report No.: 1470-0328 Contract No.: ISBN 978 0 9925013 1 0.

24. Widmark C, Leval A, Tishelman C, Ahlberg BM. Obstetric care at the intersection of science and culture: Swedish doctors' perspectives on obstetric care of women who have undergone female genital cutting. J Obstet Gynaecol. 2010;30(6):553-8.

25. Widmark C, Tishelman C, Ahlberg BM. A study of Swedish midwives' encounters with infibulated African women in Sweden. Midwifery. 2002;18(2):113-25.

26. Burchill J, Pevalin DJ. Demonstrating cultural competence within health-visiting practice: working with refugee and asylum-seeking families. Divers Equal Health Care. 2014;11(2):151-9.

27. Bulman K, McCourt C. Report on Somali Womens' Experiences of Maternity Services. London, UK: Centre for Midwifery Practice, Wolfson Institute of Health Sciences, Thames Valley University and Hammersmith Hospitals National Health Service Trust; 1997.

28. Byrskog U, Olsson P, Essen B, Allvin MK. Being a bridge: Swedish antenatal care midwives' encounters with Somali-born women and questions of violence - a qualitative study. BMC Pregnancy Childbirth. 2015;15(1).

29. Rubin EA. When Cultures Collide: An Exploration of Cultural Competence and Cross-Cultural Communication between American Medical Providers and Immigrant Women who have been Circumcised. PhD Thesis, USA: University of Massachusetts; 2000.

30. Abdi R. Carving culture: Creating identity through female genital cutting. Durham Anthropology Journal. 2012;18(1):115-53.
